# Unsymmetrically Substituted Dibenzo[*b,f*][1,5]-diazocine-6,12(5*H*,11*H*)dione—A Convenient Scaffold for Bioactive Molecule Design

**DOI:** 10.3390/molecules25040906

**Published:** 2020-02-18

**Authors:** Bartosz Bieszczad, Damian Garbicz, Damian Trzybiński, Damian Mielecki, Krzysztof Woźniak, Elżbieta Grzesiuk, Adam Mieczkowski

**Affiliations:** 1 Institute of Biochemistry and Biophysics, Polish Academy of Sciences, Pawińskiego 5a, 02-106 Warsaw, Poland; dgarbicz@ibb.waw.pl (D.G.); damian@ibb.waw.pl (D.M.); elag@ibb.waw.pl (E.G.); 2 Biological and Chemical Research Centre, University of Warsaw, Żwirki i Wigury 101, 02-089 Warsaw, Poland; dtrzybinski@cnbc.uw.edu.pl (D.T.); kwozniak@chem.uw.edu.pl (K.W.)

**Keywords:** unsymmetrical dibenzo[*b,f*][1,5]diazocines, isatoic anhydrides, crystallographic analysis, cytotoxicity, heterocycles

## Abstract

A novel approach for the synthesis of unsymmetrically substituted dibenzo[*b,f*][1,5]diazocine-6,12(5*H*,11*H*)diones has been developed. This facile three-step method uses variously substituted 1*H*-benzo[*d*][1,3]oxazine-2,4-diones (isatoic anhydrides) and 2-aminobenzoic acids as a starting materials. The obtained products were further transformed into *N*-alkyl-, *N*-acetyl- and dithio analogues. Developed procedures allowed the synthesis of unsymmetrical dibenzo[*b,f*][1,5]diazocine-6,12(5*H*,11*H*)diones and three novel heterocyclic scaffolds: benzo[*b*]naphtho[2,3-*f*][1,5]diazocine-6,14(5*H*,13*H*)dione, pyrido[3,2-*c*][1,5]benzodiazocine-5,11(6*H*,12*H*)-dione and pyrazino[3,2-*c*][1,5]benzodiazocine-6,12(5*H*,11*H*)dione. For 11 of the compounds crystal structures were obtained. The preliminary cytotoxic effect against two cancer (HeLa, U87) and two normal lines (HEK293, EUFA30) as well as antibacterial activity were determined. The obtained dibenzo[*b,f*][1,5]diazocine(5*H*,11*H*)6,12-dione framework could serve as a privileged structure for the drug design and development.

## 1. Introduction

**Figure 1:**
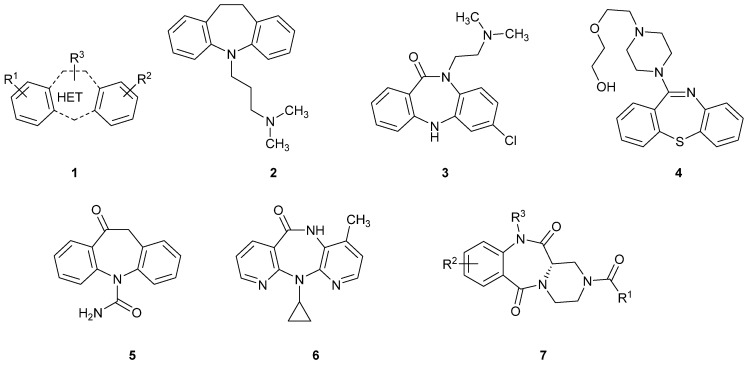
Tricyclic 7-th membered dibenzoheterocycles and their analogues.

Tricyclic dibenzodiazepines and their structural analogues (dibenzoazepines, dibenzothiazepines and others) having two non-polar, aromatic benzene or heterocyclic rings separated by a seven-membered ring containing heteroatoms such as sulfur, nitrogen or oxygen (**1**, Figure 1), are very popular, privileged structures, useful for the development of drugs, as well as compounds with various biological activities and applications (Figure 1). Modulation of biological properties is possible by skillful chemical modifications, and variation of substituents and functional groups located in the six-membered benzene rings and the seven-membered heterocyclic ring. This group of compounds includes such important drugs as imipramine (**2**; a dibenzoazepine with antidepressant activity and a serotonin and norepinephrine reuptake inhibitor) [1], clobenzepam (**3**; dibenzodiazepine; antihistaminic and anticholinergic) [2], quetiapine (**4**; dibenzothiazepine; antipsychotic activity; dopamine, serotonin, and adrenergic receptors antagonist) [3], oxcarbazepine (**5**; dibenzoazepine; anticonvulsant activity; voltage-sensitive sodium channels blocker) [4,5], and nevirapine (**6**; dipyridodiazepine; anti-HIV; non-nucleoside reverse transcriptase inhibitor) [6]. Additionally, we recently reported [7] the synthesis of structurally related, tricyclic pyrazinebenzodiazepines type **7**. Surprisingly, these compounds exhibited a promising cytotoxic effect against two cancer cell lines: LoVo (human colon cancer) and MV-4-11 (biphenotypic B myelomonocytic leukemia) [8].

**Figure 2:**
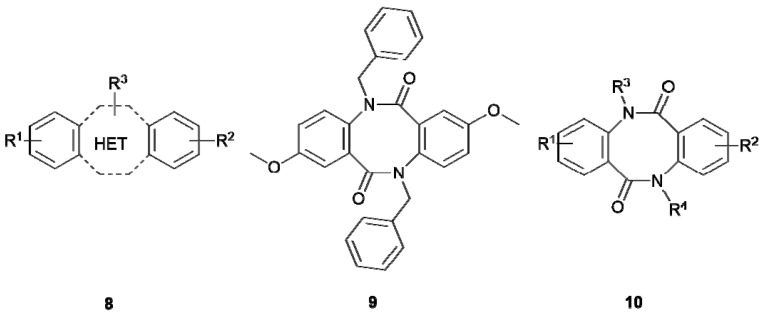
Tricyclic, 8-membered dibenzoheterocycles.

Tricyclic dibenzoheterocycles **2**–**5**, and their tricyclic analogues possessing pyridine **6** or piperidine **7** rings are widely used in the design and search for new compounds of biological importance. On the other hand, analogous tricyclic systems containing two non-polar, aromatic benzene rings separated by a larger, eight-membered heterocyclic ring—tricyclic dibenzodiazocines and their structural analogs (**8**, Figure 2) are not practically used in the design of biologically active compounds despite their high potential. This situation is caused by the lack of appropriate synthetic routes leading to this type of compounds. A rare example of the application of such heterocyclic scaffolds in the design of compounds exhibiting biological activity involves the use of compound **9** (Figure 2) as a chemosensitizer, abolishing the activity of a glycoprotein (GP 170) located in the cell membrane, and increasing the penetration of the drug into the cell [9,10].

**Scheme 1:**
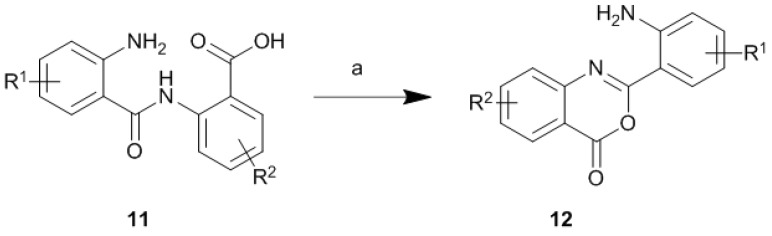
DCC-mediated cyclisation of 2-(2-aminobenzamido)benzoic acid **11**: Reagents and Conditions: (a) DCC, DMF, rt, 18 h.

In our continuous research on the development of medicinally relevant compounds possessing the dilactam structure [7,8], we recently focused on the dibenzo[*b,f*][1,5]diazocine scaffold **10** as a possible framework for the design of biologically active substances. Our literature survey revealed that symmetrical compounds type **10** (R^1^ = R^2^, R^3^ = R^4^) could be obtained in the dimerization reaction of substituted 2-aminobenzoic acids (anthranilic acids) in the presence of phosphorus oxychloride [9] or in the dimerization reaction of alkyl 2-aminobenzoates after treatment with sodium hydride [11]. These methods, based on the dimerization of two moieties of 2-aminobenzoic acids or their esters, allow for high-yield syntheses of symmetrical dibenzo[*b,f*][1,5]diazocines but are completely unsuitable for synthesis of more complex and unsymmetrical compounds type **10**, possessing various substituents in the aromatic and dilactam rings (R^1^ ≠ R^2^, R^3^ ≠ R^4^). It is obvious that by limiting the ability to modify the structure of a compound, one reduces its potential use in the design of biologically active agents. This limits the modulation of biological activity, as well as physicochemical properties, where the key to success is the skilful introduction and modification of substituents and side chains attached to the heterocyclic scaffold. Compounds possessing the dibenzo[*b,f*][1,5]diazocine structure **10**, could be also treated as small cyclic dipeptides, consisting of two aromatic β-amino acid units. Consequently, synthetic efforts have been directed toward cyclisation of 2-(2-aminobenzamido)benzoic acids **11** (Scheme 1) in the presence of classical peptide coupling reagents [11,12]. Unfortunately, as reported [12], and confirmed in our laboratory, this approach led exclusively to the formation of yellow-coloured, bicyclic products possessing the 2-(2-aminophenyl)-4*H*-benzo[*d*][1,3]oxazin-4-one structure **12**. We also observed, and proved by the single-crystal X-ray diffraction analysis, that crystallization of compound **12** (R^1^ = R^2^ = H) from water-methanol mixture, in the presence of *p*-toluenesulphonic acid (1 equivalent), led to its hydrolysis to 2-(2-aminobenzamido)benzoic acid **11** (R^1^ = R^2^ = H), crystallized as a 4-toluenesulphonate salt 11*TsOH (for the crystallographic details see the Supporting Information file). The application of some other cyclisation agents such as polyphosphate ester (PPE) [12] or thionyl chloride [13] for cyclisation of primary amides also led to the product **12**, while treatment of the *N*-methyl amide with PPE resulted in the formation of the dibenzo[*b,f*][1,5]diazocine scaffold [12]. These results suggest that the use of cyclisation agents such as carbodiimides, PPE or thionyl chloride requires substitution or protection of amide group is necessary to avoid formation of **12**.

The only attempt toward the synthesis of unsymmetrical compounds type **10** (R^1^ ≠ R^2^, R^3^ ≠ R^4^) was reported in 2004 [13], where unprotected 2-aminobenzoic acids were initially treated with thionyl chloride, and the obtained intermediates were coupled with a second molecule of unprotected, differentially *C*-substituted 2-aminobenzoic acids. That short letter was only limited to the general procedure of synthesis and physicochemical data for just one representative product containing the dibenzo[*b,f*][1,5]diazocine structure. In contrast to the high yields of syntheses reported in the article, in our hands this method led to the complex mixture of products, including benzo[*d*][1,3]oxazin-4-ones **12** and uncyclized **11**. For this reason, we focused on the search for the alternative methods for the synthesis of unsymmetrical dibenzo[*b,f*][1,5]diazocines **10** (R^1^ ≠ R^2^, R^3^ ≠ R^4^). In this paper, we present a high-yielded, three-step procedure based on the synthesis of unsymmetrical and variously substituted 2-(2-aminobenzamido)benzoic acids esters, followed by basic cyclisation in the presence of sodium hydride.

## 2. Results and Discussion

### 2.1. Chemistry

**Scheme 2:**
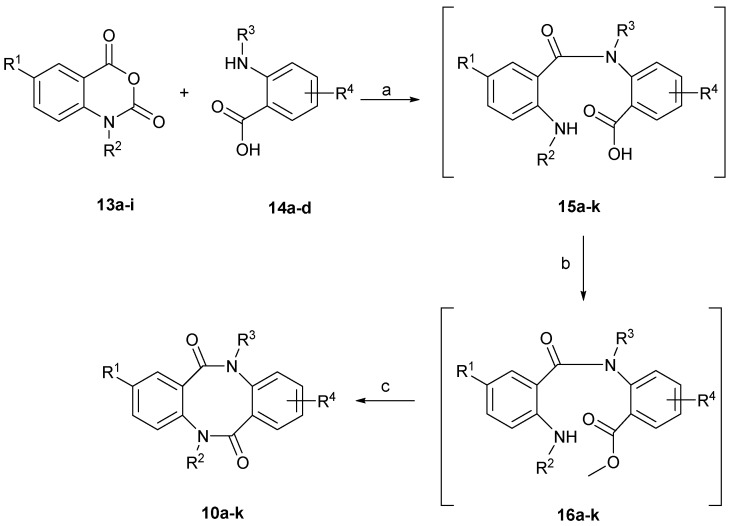
First synthetic strategy to the dibenzo[*b*,*f*][1,5]diazocine scaffold. Reagents and Conditions: (a) NaOH, H_2_O, 80 °C, 30 min; (b) H_2_SO_4_, MeOH, reflux, 72 h; (c) THF_anh._, 60% NaH, reflux, 18 h.

In the present study, we developed two different synthetic approaches to the preparation of asymmetrically substituted dibenzo[*b,f*][1,5]diazocine scaffold **10**. The first method is based on the application of variously N- and C- and N,C-disubstituted 1*H*-benzo[*d*][1,3]oxazine-2,4-diones (isatoic anhydrides) **13a–i** as coupling partners with variously N- and C-substituted, unprotected 2-aminobenzoic acids **14a**–**d** (Scheme 2).

The previous literature reported one example of the application of 1*H*-benzo[*d*]- [1,3]oxazine-2,4-diones for the synthesis of the dibenzo[*b,f*][1,5]diazocine scaffold **10**, which was limited to symmetrically substituted products [14]. We observed that unsubstituted 1*H*-benzo[*d*][1,3]oxazine-2,4-dione (**13c**), *C*-substituted: 6-chloro (**13a**), 6-bromo- (**13b**) and 6-nitro-1*H*-benzo[*d*][1,3]oxazine-2,4-dione (**13d**), *N*-substituted: 1-methyl- (**13e**), 1-benzyl- (**13g**) and 1-(4-bromobenzyl)-1*H*-benzo[*d*][1,3]oxazine-2,4-dione (**13h**), as well as *C,N*-disubstituted: 6-chloro-1-methyl- (**13f**) and 6-bromo-1-(naphthalen-1-ylmethyl)-1*H*-benzo[*d*][1,3]oxazine-2,4-dione (**13i**) could be used as coupling partners with unsubstituted 2-aminobenzoic acid (**14a**), *C*-substituted: 4,5-dimethoxy-2-aminobenzoic acid (**14b**) and 3-amino-2-naphthoic acids (**14d**) and *N*-substituted 2-(methylamino)benzoic acid (**14c**) (Table 1). The coupling step requires the presence of base, one equivalent of sodium hydroxide, and is performed in an aqueous solution at 80 °C [11]. The obtained non-symmetrically substituted 2-(2-aminobenzamido)benzoic acids **15a–k** were not isolated but directly transformed into the appropriate methyl esters **16a–k**, by refluxing **15a–k** in methanol, in the presence of concentrated sulfuric acid [11]. The final cyclisation step of crude methyl 2-(2-aminobenzamido)benzoates **16a–k** was performed with sodium hydride in refluxing anhydrous THF, resulting in dibenzo[*b,f*][1,5]diazocines **10a–k**. In the case of compounds **10a–e**, **10h** and **10j** the yield of this three-step synthesis was in the range of 30–35%, giving quite high efficiency for a single step synthesis (average yield for one synthesis step). In the case of compounds **10f**, **10g** and **10i**, we observed slightly lower efficiency, in the 25–29% range, while compound **10k** was obtained with 18% yield.

**Scheme 3:**
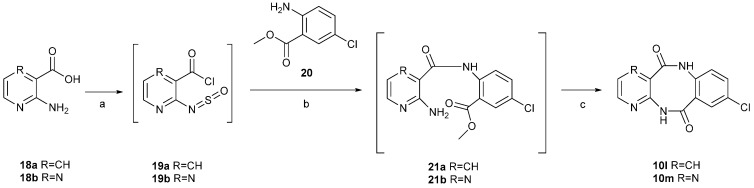
Second synthetic strategy to obtain the tricyclic diazocine-5,11(6*H*,12*H*)-dione scaffold. Reagents and Conditions: (a) SOCl_2_, toluene, reflux, 3 h; (b) toluene, rt, 48 h; (c) THF_anh._, 60% NaH, reflux, 18 h.

Unfortunately, the unsubstituted 2-aminonicotinic acid (**18a**) and 3-amino-2-pyrazinecarboxylic acid (**18b**) failed to react with 1*H*-benzo[*d*][1,3]oxazine-2,4-diones, which forced us to investigate an alternative method for coupling of **18a-b** with 2-aminobenzoic acids. In the second method, 2-aminonicotinic acid (**18a**) and 3-amino-2-pyrazinecarboxylic acid (**18b**) were transformed in the first synthesis step into 2-amino-*N*-sulfinylnicotinoyl chloride (**19a**) and 3-amino-*N*-sulfinylpyrazine-2-carbonyl chloride (**19b**) after treatment with SOCl_2_ in boiling toluene (Scheme 3) [13]. 

The resulting crude intermediates **19a,b** underwent reaction with 2-amino-5-chlorobenzoate (**20**) to form methyl 2-(2-aminonicotinamido)-5-chlorobenzoate (**21a**) and methyl 2-(2-aminopyrazine-3-carboxamido)-5-chlorobenzoate (**21b**). Intermediates **21a,b** were not isolated, but rather directly treated with sodium hydride in refluxing anhydrous THF, which led to the formation of 8-chloropyrido[3,2-*c*][1,5]benzodiazocine-5,11(6*H*,12*H*)-dione (**10l**), in 17% yield, and 8-chloropyrazino[3,2-*c*][1,5]benzodiazocine-6,12(5*H*,11*H*)-dione (**10m**), in 19% yield (total yield for the three-step synthesis).

**Scheme 4:**
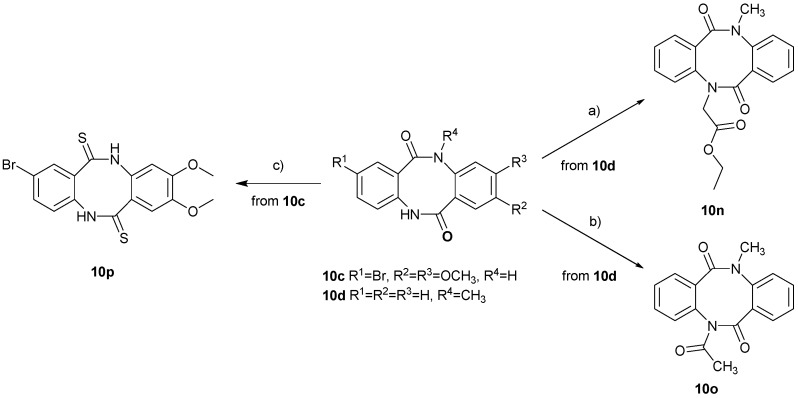
Reagents and Conditions: (a) ethyl bromoacetate, 60% NaH, DMSO, rt, 18 h; (b) Ac_2_O, reflux, 3 h; (c) *p*-tolyl Davy-reagent, THF_anh._, reflux, 18 h.

We also performed post-cyclisation modifications of the reported dibenzo[*b*,*f*][1,5]diazocines, including alkylation, acylation and thiolation of 8-membered dilactam diazocine-6,12-dione ring (Scheme 4). The treatment of 5-methyldibenzo[*b*,*f*][1,5]diazocine-6,12(5*H*,11*H*)-dione (**10d**) with ethyl bromoacetate, in the presence of 60% sodium hydride in mineral oil, led to the formation of ethyl 2-(11-methyl-6,12-dioxo-11,12-dihydrodibenzo[*b*,*f*][1,5]diazocin-5(6*H*)-yl)acetate (**10n**), in 78% yield. Heating of 5-methyldibenzo[*b*,*f*][1,5]diazocine-6,12(5*H*,11*H*)-dione (**10d**) in boiling acetic anhydride for 3 h, resulted in the *N*-acyl derivative, 5-acetyl-11-methyldibenzo[*b*,*f*][1,5]diazocine- 6,12(5*H*,11*H*)-dione (**10o**), in 89% yield. Finally, heating of 8-bromo-2,3-dimethoxy- dibenzo[*b*,*f*][1,5]diazocine-6,12(5*H*,11*H*)-dione (**10c**) with *p*-tolyl Davy-reagent in boiling anhydrous THF for 18 h, led to the formation of the corresponding dithiolactam derivative, 8-bromo-2,3-dimethoxydibenzo[*b*,*f*][1,5]diazocine-6,12(5*H*,11*H*)-dithione (**10p**), in 58% yield.

### 2.2. Crystallographic Analysis

**Figure 3:**
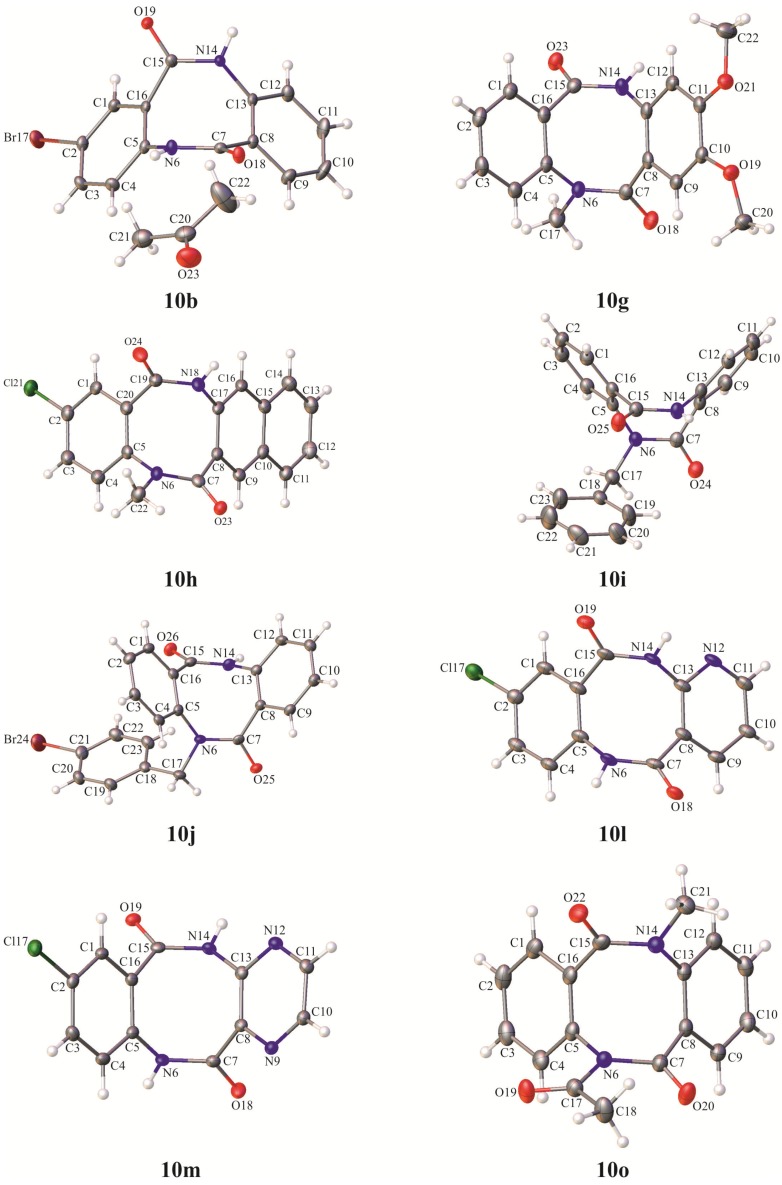
Asymmetric unit of the crystal lattice of final products **10b**, **10g**–**j**, **10l**, **10m**, **10o** with crystallographic atom numbering. Displacement ellipsoids are drawn at the 50% probability level. The H-atoms are shown as small spheres of arbitrary radius.

Attempts to obtain single-crystals suitable for X-ray diffraction measurements of all synthesized dibenzo[*b,f*][1,5]diazocine-6,12(5*H*,11*H*)-diones **10a**–**o** were undertaken. However, they were successful in only eight cases (**10b**, **10g**, **10h**, **10i**, **10j**, **10l**, **10m** and **10o**) (Figure 3). The crystal structures of three intermediates (11*TsOH, **13i** and **14c**) were also determined (Figure 1, Figure 2 and Figure 3 (ESI)). Crystals appropriate for diffractometric analysis were grown by slow evaporation from ethanol (**10h**, **10j**, **10m**), acetone (**10b**, **10l**), ethyl acetate (**10g**, **13i**), hexane:ethyl acetate (**14c**), cyclohexane:ethyl acetate (**10o**), and water:methanol (11*TsOH) at room temperature.

**Figure 4:**
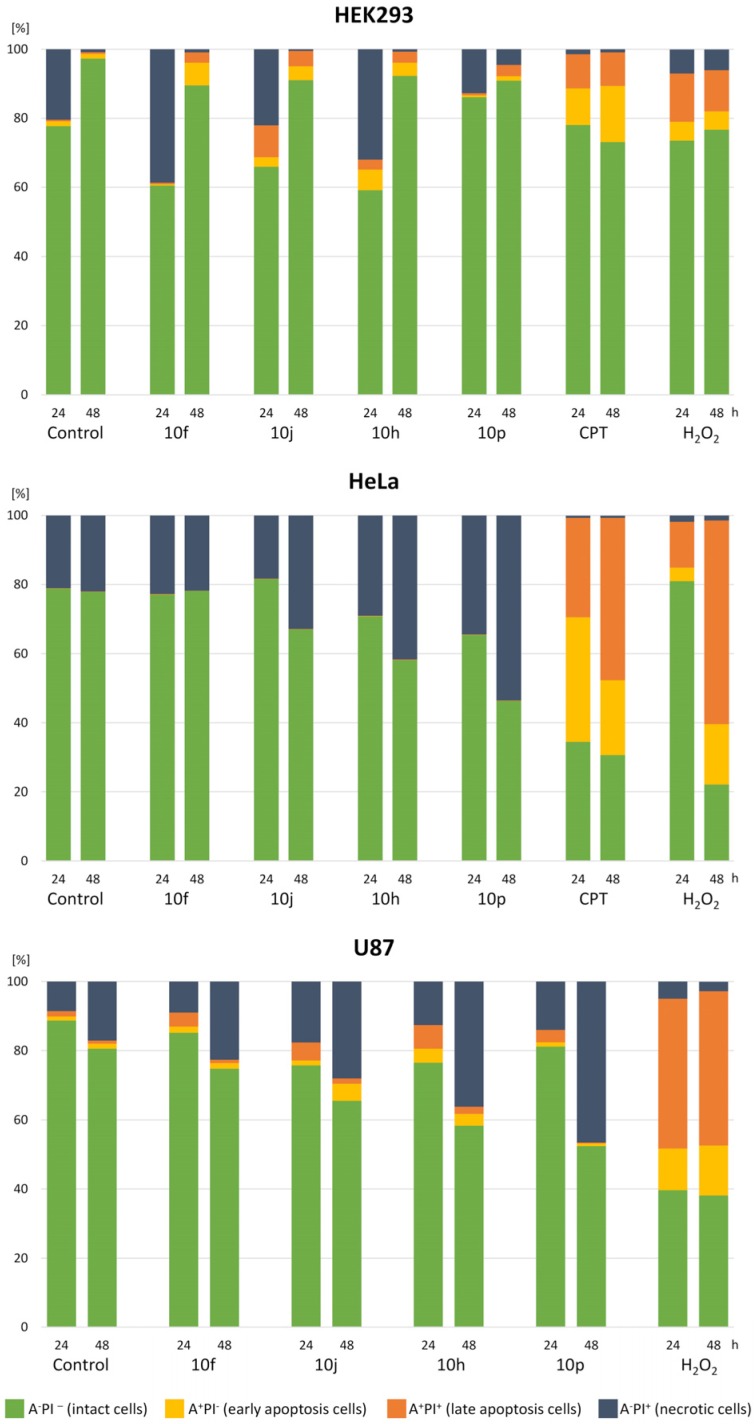
Flow cytometry analysis of HEK293, HeLa and U87 cells stained with Annexin-FITC (A) and propidium iodide (PI). Cells were treated with 200 µM compounds for 24 or 48 h. Camptothecin (10 μM) and H2O2 (100 μM) were used as experimental controls.

The investigated compounds crystallized in the monoclinic *P*2_1_/*c* (11*TsOH, **13i**, **14c**, **10b**, **10g**, **10l**, and **10m**) or *C*2/*c* (**10i**), orthorhombic *P*2_1_2_1_2_1_ (**10h**) or *Pbca* (**10o**) and triclinic *P*-1 (**10j**) space groups, with one molecule of a compound in the asymmetric unit of the crystal lattice (Figure 4, Figures S1 and S2). The asymmetric unit of **10b** contains one molecule of solvent (acetone). The conformation of **10a-o** resembles a butterfly. The refinement parameters and details of the crystallographic data are presented in Table S1 (ESI). The values of valence and torsion angles, together with bond lengths are presented in Tables S2−S4 (ESI).

### 2.3. Cytotoxic and Antibacterial Effect of dibenzo[b,f][1,5]diazocine-6,12-diones ***10a–p***

We evaluated the cytotoxic efficacy of 16 synthesized dibenzo[*b*,*f*][1,5]diazocine-6,12-diones **10a–p** on two normal (HEK293, EUFA30) and two cancerous (HeLa, U87) cell lines (concentrations tested: 1–200 µM; Table 2). Among the tested compounds, five showed a cytotoxic effect while maintaining the selectivity of action—**10b**, **10f**, **10h**, **10j** and **10p**—their IC_50_ ranged from several dozen (lowest—97.3 µM) to several hundred (higher—205.7 µM). Nevertheless, the IC_50_ for normal cell lines was higher than for cancerous ones. For the majority of the remaining compounds, the IC_50_ values were above 1 mM or could not be calculated.

Although the evaluated compounds showed a rather weak cytotoxic effect, we were able to establish some relationships between the structure of the compounds and their activity. 2-Bromodibenzo[*b*,*f*][1,5]diazocine-6,12(5*H*,11*H*)-dione (**10b**) showed a very weak cytotoxic effect with some selectivity for the HeLa tumor cell line (IC_50_ = 97.3 μM). Its *N*-methyl derivative, 2-bromo-11-methyldibenzo[*b*,*f*][1,5]diazocine-6,12(5*H*,11*H*)-dione (**10e**), showed a weaker cytotoxic effect than the parent compound (IC_50_ ˃ 200 μM for HeLa). The introduction of an additional naphthalen-1-ylmethyl group resulted in the formation of 2-bromo-11-methyl- 5-(naphthalen-1-ylmethyl)dibenzo[*b*,*f*][1,5]diazocine-6,12(5*H*,11H)-dione (**10k**) showing noticeable cytotoxicity on all cell lines tested (IC_50_ = 87.6 μM—EUFA, 119.0 μM—HEK293, 75.3 μM—HeLa, 75.4 μM—U87). We observed that the presence of an additional, large and hydrophobic substituent attached to the dilactam ring was beneficial for enhancement of the cytoxic effect. 2-Chlorodibenzo[*b*,*f*][1,5]diazocine-6,12(5*H*,11*H*)-dione (**10a**) and 8-bromo-2,3-dimethoxydibenzo- [*b*,*f*][1,5]diazocine-6,12(5*H*,11*H*)-dione (**10c**) showed no cytotoxic effect in the concentration range tested. Structural modifications of **10a**: introduction of another benzene ring into the heterocyclic scaffold and a methyl group into the dilactam ring led to 2-chloro-5-methyl- benzo[*b*]naphtho[2,3-*f*][1,5]diazocine-6,14(5*H*,13*H*)-dione (**10h**). These structural modifications resulted in the enhancement of the cytotoxicity of **10h** which exhibited cytotoxic effect for all tested cell lines (IC_50_ = 170.5 μM—EUFA, ˃ 200 μM—HEK293, 107.4 μM—HeLa, 148.6 μM—U87). Finally, the conversion of **10c** into its dithiolactam analogue: 8-bromo-2,3-dimethoxy- dibenzo[*b*,*f*][1,5]diazocine-6,12(5*H*,11*H*)-dithione (**10p**) caused the appearance of a cytotoxic effect on cancer cell lines (IC_50_ ˃ 200 μM—EUFA, ˃ 200 μM—HEK293, 115.2 μM—HeLa, 170.2 μM—U87) suggesting that such structural modification is beneficial for increasing the cytotoxic effect of these compounds.

To measure the induction of apoptosis, cells were treated with 200 µM of **10f**, **10h**, **10j**, **10k** and **10p** compound, for 24 and 48 h (Figure 4). The experiment was performed on one normal (HEK293) and two cancer (HeLa, U87) cell lines. After 24 h of treatment, the HEK293 cells showed significant increase in necrosis and late apoptosis, as follows: 20% and 0.4% in control cells, 39% and 0.5% for **10f**, 32% and 3% for **10h**, 22% and 10% for **10j**. After 48 h of treatment, a slight increase in early and late apoptosis was observed, namely, 6.6% and 3% for **10f**, 3.8% and 3.2% for **10h**, 4% and 4.4% for **10j,** in comparison to 1.3% and 0.5% in control cells.

The HeLa cells were the most sensitive to compound **10p**, with a significant increase only in necrotic phase. After 24 h, the percentage of necrotic cells was 34.5% in comparison to 21.1% in the non-treated control, and 53.4% in comparison to 22.1% in control cells after 48 h treatment. The U87 cells were sensitive to three out of five compounds tested. In the control, after 24 h, there were 1.2% of cells in early apoptosis, 1.6% in late apoptosis, and 8.6% in necrosis. For **10h** treatment, cells were at 4.1% in early apoptosis and 6.9% in late apoptosis; for **10j** at 5.2% in late apoptosis and 17.8% in necrosis; and for **10p** at 14% in necrosis. After 48 h, we observed a significant increase in necrotic phase only, as follows: in control to 17%, for **10h** to 36.2%, for **10j** to 28.1%, and for **10p** to 46.6%.

Although compound **10k** showed cytotoxicity to all tested human cell lines, we did not observe any changes in the apoptosis/necrosis phases in relation to control. It is possible that the compound shows not cytotoxic but cytostatic activity.

Many different natural products possessing a lactam ring (such as β-lactam antibiotics [15]) or dilactam ring (diketopiperazines [16]) exhibit a strong antimicrobial effect. For this reason, we evaluated compounds **10a**–**o** for their antibacterial activity. Antibacterial studies were carried out against two Gram-positive (*Bacillus megaterium*, *Staphylococcus aureus* RN4220) and six Gram-negative bacterial strains (*Escherichia coli* AB1157, *Pseudomonas putida* KT2440, *Shewanella oneidensis* MR-1, *Salmonella typhimurium* TA98, *Salmonella typhimurium* TA100, *Pseudomonas aeruginosa* PAO1). The results showed none of the synthesized compounds exhibited antibacterial properties at the concentrations of 12.5, 33.3, 50 and 100 µM. Even though the obtained products exhibited rather weak cytotoxic and no antibacterial activity in the tested range of concentrations, further research with the use of dibenzo[*b,f*][1,5]diazocine-6,12(5*H*,11*H*)diones as privileged structures useful in the design of bioactive compounds is underway in our laboratory and will be published in due course.

## 3. Materials and Methods

### 3.1. Chemistry

#### 3.1.1. General Information

Commercially available chemicals were of reagent grade and used as received. Purification of the compounds was performed by column chromatography on silica gel 60 M (0.040–0.063 mm, E. Merck, Darmstadt, Germany). Thin layer chromatography (TLC), using silica gel plates (Kieselgel 60F_254_, E. Merck), was used to monitor reaction progress. A B-540 Melting Point apparatus (Büchi, New Castle, DE, USA) was used to measure melting points. The ^1^H-NMR and ^13^C-NMR spectra, in DMSO-*d*_6_ and CDCl_3_, were recorded at the Department of Chemistry, University of Warsaw, using an AVANCE III HD 300 MHz spectrometer (Bruker, Billerica, MA, USA); shift values in parts per million are relative to the SiMe_4_ internal reference. The resonance assignments were based on peak multiplicity and peak integration of recorded spectra, Multiplets were assigned as bs (broad singlet), s (singlet), d (doublet), t (triplet) dd (doublet of doublet), ddd (doublet of doublet of doublet) and m (multiplet). An LTQ Orbitrap Velos instrument (Thermo Scientific, Waltham, MA, USA) located at the Mass Spectrometry Laboratory of the Institute of Biochemistry and Biophysics PAS (Warsaw, Poland) was used to record high resolution mass spectra. A 6200 FT/IR spectrometer (Jasco, Easton, MD, USA) at the Laboratory of Optical Spectroscopy (Institute of Organic Chemistry PAS, Warsaw, Poland) was used to record IR spectra.

#### 3.1.2. General Procedure for the Synthesis of 2-(2-aminobenzamido)benzoic acids **15a–k**

A suspension of 1*H*-benzo[*d*][1,3]oxazine-2,4-dione **13a**–**i** (1 equiv.), 2-aminobenzoic acid **14a**–**d** (1 equiv.) and sodium hydroxide (1 equiv.) in water (10 mL/mmol) was heated at 80 °C for 30 min until the evolution of carbon dioxide had ceased and a clear solution had formed. After cooling the reaction mixture, the obtained solution was diluted with water, and the crude product was precipitated by addition of glacial acetic acid, filtered and dried under vacuum. In cases of *N*-alkyl 1*H*-benzo[*d*][1,3]oxazine-2,4-diones where a gummy-like residue was formed, the crude product was extracted with ethyl acetate, the organic phase was washed with brine and dried over anhydrous magnesium sulfate. Evaporation of the solvent left a glass-like residue which was used in next step without further purification.

#### 3.1.3. General Procedure for the Synthesis of 2-(2-aminobenzamido)benzoic acids methyl esters **16a**–**k**

2-(2-Aminobenzamido)benzoic acids **15a**–**k** were dissolved in methanol (*ca*. 10 mL/mmol) then, concentrated sulfuric acid was added (0.5 mL/mmol) (caution: exothermic). The resulted solution was refluxed for 72 h. The excess of methanol was evaporated and the resulting residue was added to water. The pH was adjusted to 8 by addition of NaOH and the crude products were extracted with ethyl acetate. The combined organic layers were washed with 1 N sodium hydroxide, water and brine, and then dried over anhydrous magnesium sulfate. Evaporation of solvent yielded a dark residue of crude methyl esters **16a**–**k**, which were used in the next step without further purification.

#### 3.1.4. General Procedure for the Synthesis of dibenzo[*b*,*f*][1,5]diazocine-6,12(5*H*,11*H*)-diones **10a**–**k** from 2-(2-aminobenzamido)benzoic acids methyl esters **16a**–**k**

2-(2-Aminobenzamido)benzoic acids methyl esters **16a**–**k** were dissolved in the anhydrous THF (20 mL/mmol) then 60% sodium hydride in mineral oil (2 equiv.) was added and the resulted solution was refluxed for 18 h. The excess of THF was evaporated, the obtained residue was poured into 1 N HCl and the crude product was extracted with ethyl acetate. The combined organic layers were washed with 1 N HCl, water and brine, then dried over anhydrous magnesium sulfate. Crude products were purified by column chromatography using hexane/EtOAc 1:1 then 2:8 *v/v* as eluent.

*2-Chlorodibenzo[b,f][1,5]diazocine-6,12(5H,11H)-dione* (**10a**): Yield 33%, colourless crystals, m.p. 275–276 °C, R.f. = 0.40 (hexane:ethyl acetate 2:8 *v/v*). ^1^H-NMR (DMSO-*d*_6_) δ 10.33 (s, 1H, NH), 10.25 (s, 1H, NH), 7.45–7.21 (m, 5H, H_Ar_), 7.11 (d, 1H, *J* = 2.4 Hz, H_Ar_), 7.09 (d, 1H, *J* = 1.8 Hz, H_Ar_); ^13^C-NMR (DMSO-*d*_6_) δ 169.1, 167.6, 135.3, 134.4, 133.8, 133.3, 131.4, 130.7, 130.4, 128.2, 127.7, 127.6, 127.5, 125.9; IR (KBr): cm^−1^ 3183, 3055, 2900, 1658, 1601, 1578, 1484, 1415, 1366, 1261, 1229, 1145, 1111; HRMS (ESI): *m/z* [M+H]^+^ calcd for C_14_H_10_ClN_2_O_2_: 273.04253, 275.03958, found: 273.04202, 275.03904;

*2-Bromodibenzo[b,f][1,5]diazocine-6,12(5H,11H)-dione* (**10b**): Yield 35%, colourless crystals, m.p. 283–284 °C, R.f. = 0.43 (hexane:ethyl acetate 2:8 *v/v*). ^1^H-NMR (DMSO-*d*_6_) δ 10.33 (s, 1H, NH), 10.24 (s, 1H, NH), 7.53 (dd, 1H, *J* = 2.4, 8.4 Hz, H_Ar_), 7.49 (d, 1H, *J* = 2.4 Hz, H_Ar_), 7.42–7.21 (m, 3H, H_Ar_), 7.10 (d, 1H, *J* = 7.8 Hz, H_Ar_), 7.03 (d, 1H, *J* = 8.4 Hz, H_Ar_); ^13^C-NMR (DMSO-*d*_6_) δ 169.0, 167.5, 135.5, 134.4, 134.2, 133.3, 133.2, 130.7, 130.6, 128.2, 127.8, 127.5, 125.9, 119.6; IR (KBr): cm^−1^ 3182, 3056, 2900, 1657, 1602, 1480, 1411, 1362, 1260, 1229, 1142, 1100; HRMS (ESI): *m/z* [M+H]^+^ calcd for C_14_H_10_BrN_2_O_2_: 316.99202, 318.98997, found: 316.99140, 318.98930;

*8-Bromo-2,3-dimethoxydibenzo[b,f][1,5]diazocine-6,12(5H,11H)-dione* (**10c**): Yield 33%, colourless crystals, m.p. 195–196 °C, R.f. = 0.28 (ethyl acetate). ^1^H-NMR (DMSO-*d*_6_) δ 10.099 (s, 1H, NH), 10.057 (s, 1H, NH), 7.54 (dd, 1H, *J* = 2.4, 8.4 Hz, H_Ar_), 7.46 (d, 1H, *J* = 2.4 Hz, H_Ar_), 7.01 (d, 1H, *J* = 8.4 Hz, H_Ar_), 6.81 (s, 1H, H_Ar_), 6.65 (s, 1H, H_Ar_), 3.72 (s, 3H, OCH_3_), 3.70 (s, 3H, OCH_3_); ^13^C-NMR (DMSO-*d*_6_) δ 169.0, 167.6, 150.0, 147.7, 135.6, 134.6, 133.2, 130.6, 127.85, 127.77, 124.9, 119.4, 110.4, 109.1, 55.70, 55.66; IR (KBr): cm^−1^ 3499, 3185, 3064, 2934, 2848, 1678, 1646, 1607, 1517, 1470, 1401, 1342, 1264, 1224, 1176, 1108, 1078, 1027; HRMS (ESI): *m/z* [M+H]^+^ calcd for C_16_H_14_BrN_2_O_4_: 377.01315, 379.01110, found: 377.01246, 379.01037;

*5-Methyldibenzo[b,f][1,5]diazocine-6,12(5H,11H)-dione* (**10d**): Yield 31%, colourless crystals, m.p. 258–259 °C, R.f. = 0.36 (hexane:ethyl acetate 2:8 *v/v*). ^1^H-NMR (DMSO-*d*_6_) δ 10.25 (s, 1H, NH), 7.45–7.15 (m, 7H, H_Ar_), 7.07–6.98 (m, 1H, H_Ar_), 3.33 (s, 1H, CH_3_, partially overlapped with H_2_O signal); ^13^C-NMR (DMSO-*d*_6_) δ 168.9, 167.4, 140.1, 134.7, 134.4, 133.3, 130.9, 130.2, 128.2, 127.9, 127.5, 127.1, 125.7, 125.4, 36.5; IR (KBr): cm^−1^ 3257, 3067, 2979, 2934, 1991, 1938, 1836, 1673, 1623, 1599, 1575, 1470, 1415, 1386, 1351, 1302, 1260, 1226, 1185, 1161, 1141, 1082, 1034; HRMS (ESI): *m/z* [M+H]^+^ calcd for C_15_H_13_N_2_O_2_: 253.09715, found: 253.09682;

*2-Bromo-11-methyldibenzo[b,f][1,5]diazocine-6,12(5H,11H)-dione* (**10e**): Yield 30%, colourless crystals, m.p. 247–248 °C, R.f. = 0.46 (hexane:ethyl acetate 2:8 *v/v*). ^1^H-NMR (DMSO-*d*_6_) δ 10.30 (s, 1H, NH), 7.53–7.25 (m, 6H, H_Ar_), 7.00 (d, 1H, *J* = 8.4 Hz, H_Ar_), 3.32 (s, 1H, CH_3_, partially overlapped with H_2_O signal); ^13^C-NMR (DMSO-*d*_6_) δ 186.7, 165.8, 139.7, 136.3, 134.2, 133.1, 132.9, 131.2, 130.3, 128.5, 127.6. 127.5, 125.8, 119.4, 36.5; IR (KBr): cm^−1^ 3172, 3087, 2881, 1681, 1635, 1597, 1473, 1427, 1399, 1370, 1345, 1300, 1274, 1240, 1180, 1135, 1079, 1038, 1014; HRMS (ESI): *m/z* [M+H]^+^ calcd for C_15_H_12_BrN_2_O_2_: 331.00767, 333.00562, found: 331.00718, 333.00503;

*11-Methyl-2-nitrodibenzo[b,f][1,5]diazocine-6,12(5H,11H)-dione* (**10f**): Yield 25%, colourless crystals, m.p. 225–226 °C, R.f. = 0.46 (hexane:ethyl acetate 2:8 *v/v*). ^1^H-NMR (DMSO-*d*_6_) δ 10.74 (s, 1H, NH), 8.18-8.09 (m, 2H, H_Ar_), 7.50–7.41 (m, 2H, H_Ar_), 7.36–7.26 (m, 3H, H_Ar_), 3.37 (s, 1H, CH_3_, overlapped with H_2_O signal); ^13^C-NMR (DMSO-*d*_6_) δ 168.6, 165.3, 145.5, 140.5, 139.6, 134.9, 132.4, 131.4, 128.7, 127.8, 126.4, 125.9, 125.3, 123.6, 36.6; IR (KBr): cm^−1^ 3177, 3056, 2907, 1946, 1712, 1676, 1628, 1528, 1485, 1335, 1260, 1239, 1178, 1137, 1089; HRMS (ESI): *m/z* [M+H]^+^ calcd for C_15_H_12_N_3_O_4_: 298.08223, found: 298.08160;

*2,3-Dimethoxy-11-methyldibenzo[b,f][1,5]diazocine-6,12(5H,11H)-dione* (**10g**): Yield 29%, colourless crystals, m.p. 271–272 °C, R.f. = 0.23 (ethyl acetate). ^1^H-NMR (, DMSO-*d*_6_) δ 9.97 (s, 1H, NH), 7.46–7.37 (m, 2H, H_Ar_), 7.37–7.27 (m, 3H, H_Ar_), 6.77 (s, 1H, H_Ar_), 6.59 (s, 1H, H_Ar_), 3.68 (s, 3H, OCH_3_), 3.67 (s, 3H, OCH_3_), 3.30 (s, 1H, CH_3_); ^13^C-NMR (DMSO-*d*_6_) δ 168.9, 167.3, 149.6, 147.4, 140.5, 133.3, 130.9, 128.1, 129.0, 127.6, 126.1, 125.6, 110.2, 108.7, 55.65, 55.62, 36.6; IR (KBr): cm^−1^ 3193, 3000, 2936, 2841, 1674, 1626, 1513, 1478, 1454, 1431, 1398, 1364, 1302, 1258, 1223, 1156, 1133, 1105, 1082, 1036, 1021; HRMS (ESI): *m/z* [M+H]^+^ calcd for C_17_H_17_N_2_O_4_: 313.11828, found: 313.11760;

*2-Chloro-5-methylbenzo[b]naphtho[2,3-f][1,5]diazocine-6,14(5H,13H)-dione* (**10h**): Yield 34%, beige crystals, m.p. 270–271 °C, R.f. = 0.56 (hexane:ethyl acetate 2:8 *v/v*). ^1^H-NMR (DMSO-*d*_6_) δ 10.59 (s, 1H, NH), 7.98–7.82 (m, 3H, H_Ar_), 7.62 (s, 1H, H_Ar_), 7.58–7.36 (m, 5H, H_Ar_), 3.37 (s, 1H, CH_3_); ^13^C-NMR (DMSO-*d*_6_) δ 167.6, 167.3, 138.6, 135.6, 133.4, 133.0, 132.6, 131.8, 131.1, 131.0, 128.1, 128.0, 127.78, 127.76, 127.5, 127.1, 126.9, 123.6, 36.5; IR (KBr): cm^−1^ 3157, 3117, 3052, 2965, 2924, 2887, 1939, 1913, 1839, 1768, 1670, 1630, 1594, 1475, 1420, 1351, 1302, 1274, 1228, 1164, 1129, 1106; HRMS (ESI): *m/z* [M+H]^+^ calcd for C_19_H_14_ClN_2_O_2_: 337.07383, 339.07088, found: 337.07313, 339.07024;

*5-Benzyldibenzo[b,f][1,5]diazocine-6,12(5H,11H)-dione* (**10i**): Yield 29%, beige crystals, m.p. 212–213 °C, R.f. = 0.60 (hexane:ethyl acetate). ^1^H-NMR (DMSO-*d*_6_) δ 10.26 (s, 1H, NH), 7.37–7.18 (m, 12H, H_Ar_), 7.05–7.00 (m, 1H, H_Ar_), 5.32 (d, 1H, *J* = 15.0 Hz, CH_2_), 4.81 (d, 1H, *J* = 15.0 Hz, CH_2_); ^13^C-NMR (DMSO-*d*_6_) δ 168.9, 167.8, 138.9, 136.7, 134.8, 134.3, 133.9, 130.8, 130.3, 128.3, 128.2, 127.9, 127.84, 127.76, 127.3, 127.1, 125.9, 125.4, 52.4; IR (KBr): cm^−1^ 3166, 3037, 2952, 2898, 1969, 1829, 1735, 1664, 1638, 1598, 1486, 1454, 1402, 1379, 1301, 1240, 1153, 1104, 1076, 1042; HRMS (ESI): *m/z* [M+H]^+^ calcd for C_21_H_17_N_2_O_2_: 329.12845, found: 329.12792;

*5-(4-Bromobenzyl)dibenzo[b,f][1,5]diazocine-6,12(5H,11H)-dione* (**10j**): Yield 30%, colourless crystals, m.p. 222–223 °C, R.f. = 0.66 (hexane:ethyl acetate). ^1^H-NMR (DMSO-*d*_6_) δ 10.24 (s, 1H, NH), 7.51–7.15 (m, 12H, H_Ar_), 7.06–6.98 (m, 1H, H_Ar_), 5.35 (d, 1H, *J* = 15.0 Hz, CH_2_), 4.72 (d, 1H, *J* = 15.0 Hz, CH_2_); ^13^C- NMR (DMSO-*d*_6_) δ 168.6, 167.7, 138.6, 136.1, 134.8, 134.2, 133.9, 131.2, 130.9, 130.35, 130.32, 128.4, 127.8, 127.7, 127.1, 126.0, 125.4, 120.5, 51.7; IR (KBr): cm^−1^ 3219, 3059, 2939, 1901, 1672, 1633, 1598, 1488, 1467, 1395, 1353, 1307, 1285, 1258, 1217, 1158, 1103, 1068, 1024, 1009; HRMS (ESI): *m/z* [M+H]^+^ calcd for C_21_H_16_BrN_2_O_2_: 407.03897, 409.03692, found: 407.03841, 409.03626;

*2-Bromo-11-methyl-5-(naphthalen-1-ylmethyl)dibenzo[b,f][1,5]diazocine-6,12(5H,11H)-dione* (**10k**): Yield 18%, colourless crystals, m.p. 255–256 °C, R.f. = 0.40 (hexane:ethyl acetate 7:3 *v/v*). ^1^H-NMR (DMSO-*d*_6_) δ 8.18–8.06 (m, 1H, H_Ar_), 7.98–7.88 (m, 1H, H_Ar_), 7.82 (d, 1H, *J* = 8.1 Hz, H_Ar_), 7.60–7.22 (m, 9H, H_Ar_), 7.16–7.07 (m, 2H, H_Ar_), 6.16 (d, 1H, *J* = 15.0 Hz, CH_2_), 4.98 (d, 1H, *J* = 15.0 Hz, CH_2_), 3.05 (s, 1H, CH_3_); ^13^C-NMR (DMSO-*d*_6_) δ 166.8, 164.5, 139.8, 137.3, 136.6, 133.7, 133.4, 133.3, 131.4, 131.3, 131.0, 130.0, 128.6, 128.5, 128.4, 128.3, 127.6, 127.4, 126.3, 125.8, 125.7, 125.0, 123.3, 120.5, 48.8, 35.8; IR (KBr): cm^−1^ 3039, 3037, 3006, 2978, 2930, 2904, 2875, 1731, 1663, 1641, 1597, 1510, 1469, 1452, 1422, 1409, 1355, 1285, 1259, 1209, 1175, 1155, 1123, 1082, 1042, 1016; HRMS (ESI): *m/z* [M+H]^+^ calcd for C_26_H_20_BrN_2_O_2_: 471.07027, 473.06819, found: 471.07027, 473.06822;

#### 3.1.5. General Procedure for the Synthesis of 8-chloropyrido[3,2-*c*][1,5] benzodiazocine-5,11- (6*H*,12*H*)-dione (**10l**) and 8-chloropyrazino[3,2-*c*][1,5]benzodiazocine- 6,12(5*H*,11*H*)-dione (**10m**)

Synthesis of 2-amino-*N*-sulfinylnicotinoyl chloride (**19a**) and 3-amino-*N*-sulfinyl-pyrazine-2-carbonyl chloride (**19b**).

2-Aminonicotinic acid (**18a**) or 3-amino-2-pyrazinecarboxylic acid (**18b**) was suspended in dry toluene (10 mL/mmol), then thionyl chloride (5 equiv.) was added dropwise. The obtained slurry was refluxed for 3 h, until clear solution was formed. The excess of solvent was evaporated, and the residue was co-evaporated with toluene to remove traces of thionyl chloride. Crude products **19a,b** were used in the next step without further purification.

Synthesis of methyl 2-(2-aminonicotinamido)-5-chlorobenzoate (**21a**) and methyl 2-(2-aminopyrazine-3-carboxamido)-5-chlorobenzoate (**21b**).

2-Amino-*N*-sulfinylnicotinoyl chloride (**19a**) or 3-amino-*N*-sulfinylpyrazine-2-carbonyl chloride (**19b**) (1 equiv.) was dissolved in dry toluene (10 mL/mmol), then the solution of methyl 2-amino-5-chlorobenzoate (**20**) (1 equiv.) in toluene (10 mL/mmol) was added dropwise. The mixture was stirred at ambient temperature for 48 h, then the solvent was evaporated and the obtained residue was dissolved in ethyl acetate. The organic phase was washed two times with 1 N NaOH, water and brine, then dried over anhydrous magnesium sulfate. Evaporation of the solvent resulted in crude methyl esters **21a-b** which were used in next step without further purification.

Cyclisation of methyl 2-(2-aminonicotinamido)-5-chlorobenzoate (**21a**) and methyl 2-(2-aminopyrazine-3-carboxamido)-5-chlorobenzoate (**21b**).

Methyl 2-(2-aminonicotinamido)-5-chlorobenzoate (**21a**, 1 equiv.) and methyl 2-(2-aminopyrazine-3-carboxamido)-5-chlorobenzoate (**21b**, 1 equiv.) were dissolved in anhydrous THF (20 mL/mmol) then 60% sodium hydride in mineral oil (2 equiv.) was added. The resulting solution was refluxed for 18 h. The excess of THF was evaporated, residue was poured into 1 N HCl and the crude product was extracted with ethyl acetate. The combined organic layers were washed with 1 N HCl, water and brine, then dried with anhydrous magnesium sulfate. Crude products **10l,m** were purified by column chromatography using hexane/EtOAc 1:1 then 2:8 *v/v* as eluent.

*8-Chloropyrido[3,2-c][1,5]benzodiazocine-5,11(6H,12H)-dione* (**10l**): Yield 17%, colourless crystals, m.p. 308-309 °C, R.f. = 0.26 (hexane:ethyl acetate). ^1^H-NMR (DMSO-*d*_6_) δ 10.84 (s, 1H, NH), 10.46 (s, 1H, NH), 8.46 (dd, 1H, *J* = 1.8, 4.8 Hz, H_Ar_), 7.84 (dd, 1H, *J* = 1.8, 7.5 Hz, H_Ar_), 7.49–7.40 (m, 2H, H_Ar_), 7.48 (dd, 1H, *J* = 4.8, 7.8 Hz, H_Ar_), 7.18–7.11 (m, 1H, H_Ar_); ^13^C-NMR (DMSO-*d*_6_) δ 167.7, 167.3, 150.6, 147.0, 138.1, 134.6, 133.6, 131.7, 130.8, 128.0, 127.90, 127.64, 123.1; IR (KBr): cm^−1^ 3182, 3066, 2937, 2902, 1924, 1675, 1596, 1486, 1459, 1433, 1410, 1328, 1281, 1254, 1226, 1150, 1110; HRMS (ESI): *m/z* [M+H]^+^ calcd for C_13_H_9_ClN_3_O_2_: 274.03778, 276.03483, found: 274.0372, 276.03436;

*8-Chloropyrazino[3,2-c][1,5]benzodiazocine-6,12(5H,11H)-dione* (**10m**): Yield 19%, colourless crystals, m.p. 346–347 °C, R.f. = 0.35 (hexane:ethyl acetate). ^1^H-NMR (DMSO-*d*_6_) δ 11.24 (s, 1H, NH), 10.77 (s, 1H, NH), 8.65–8.50 (m, 2H, H_Ar_), 7.55–7.40 (m, 2H, H_Ar_), 7.30–7.18 (m, 2H, H_Ar_); ^13^C-NMR (DMSO-*d*_6_) δ 167.1, 165.5, 144.9, 144.5, 144.1, 143.7, 134.1, 132.8, 132.2, 131.1, 128.2, 127.8; IR (KBr): cm^−1^ 3249, 3164, 3057, 2961, 2891, 1928, 1685, 1571, 1537, 1484, 1458, 1406, 1343, 1276, 1236, 1211, 1150, 1133, 1108, 1064; HRMS (ESI): *m/z* [M+H]^+^ calcd for C_12_H_8_ClN_4_O_2_: 275.03303, 277.03008, found: 275.03247, 277.02957;

#### 3.1.6. Synthesis of ethyl 2-(11-methyl-6,12-dioxo-11,12-dihydrodibenzo[*b*,*f*][1,5]diazocin- 5(6*H*)-yl)acetate (**10n**)

5-Methyldibenzo[*b,f*][1,5]diazocine-6,12(5*H*,11*H*)-dione (**10d**, 252 mg, 1 mmol, 1 equiv.) was dissolved in anhydrous DMSO (5 mL), then 60% NaH dispersed in mineral oil (48 mg, 1.2 mmol, 1.2 equiv.) was added. The resulted suspension was stirred at room temperature for 30 min. until evolution of gas ceased. Then, ethyl bromoacetate (133 μL, 1.2 mmol, 1.2 equiv.) was added dropwise, and the resulting solution was stirred for 18 h at room temperature. The next day, the reaction mixture was poured into water (20 mL) and extracted with ethyl acetate (3 × 20 mL). Combined organic layers were washed with brine (1 × 20 mL) and dried over anhydrous magnesium sulfate, followed by evaporation under reduced pressure. Crude product was purified by column chromatography using hexane/EtOAc 1:1 then 2:8 *v/v* as eluent. Yield 78% (264 mg), colourless crystals, m.p. 125–126 °C, R.f. = 0.66 (hexane:ethyl acetate 2:8 *v/v*). ^1^H-NMR (DMSO-*d*_6_) δ 7.45-7.15 (m, 8H, H_Ar_), 4.62 (d, 1H, *J* = 17.1 Hz, CH_2_), 4.47 (d, 1H, *J* = 17.1 Hz, CH_2_), 4.23–4.10 (m, 2H, CH_2_), 1.22 (t, 3H, *J* = 7.2 Hz, CH_3_); ^13^C-NMR (DMSO-*d*_6_) δ 168.3, 167.6, 166.9, 140.1, 139.0, 134.3, 133.2, 131.0, 130.8, 128.2, 128.1, 127.6, 127.2, 125.6, 125.2, 60.9, 51.1, 36.1, 14.0; IR (KBr): cm^−1^ 3458, 3300, 3058, 2981, 2936, 1953, 1840, 1739, 1656, 1599, 1456, 1411, 1411, 1375, 1324, 1308, 1281, 1260, 1217, 1120, 1088, 1056, 1023; HRMS (ESI): *m/z* [M+H]^+^ calcd for C_19_H_19_N_2_O_4_: 339.13393, found: 339.13393;

#### 3.1.7. Synthesis of 5-acetyl-11-methyldibenzo[*b*,*f*][1,5]diazocine-6,12(5*H*,11*H*)-dione (**10o**)

The dispersion of 5-methyldibenzo[*b,f*][1,5]diazocine-6,12(5*H*,11*H*)-dione (**10d**) (504 mg, 2 mmol, 1 equiv.) in acetic anhydride (10 mL) was refluxed for 3 h. The excess anhydride was evaporated under reduced pressure and the residue was crystallized from a mixture of cyclohexane and ethyl acetate (9:1) to give pure product. Yield 89% (524 mg), colourless crystals, m.p. 193–194 °C, R.f. = 0.74 (hexane:ethyl acetate 2:8 *v/v*). ^1^H-NMR (DMSO-*d*_6_) δ 7.46-7.19 (m, 8H, H_Ar_), 3.34 (s, 1H, CH_3_), 2.61 (s, 1H, CH_3_); ^13^C-NMR (DMSO-*d*_6_) δ 171.5, 168.6, 166.7, 139.7, 135.4, 134.3, 133.8, 132.1, 130.3, 129.4, 129.0, 128.3, 127.8, 127.0, 126.2, 36.0, 26.7; IR (KBr): cm^−1^ 3412, 3379, 3296, 3061, 2938, 1975, 1941, 1718, 1703, 1658, 1599, 1485, 1453, 1420, 1371, 1304, 1251, 1206, 1142, 1084, 1038, 1013; HRMS (ESI): *m/z* [M+H]^+^ calcd for C_17_H_15_N_2_O_3_: 295.10772, found: 295.10735;

#### 3.1.8. Synthesis of 8-bromo-2,3-dimethoxydibenzo[*b*,*f*][1,5]diazocine-6,12(5*H*,11*H*)-dithione (**10p**)

Bromo-2,3-dimethoxydibenzo[*b,f*][1,5]diazocine-6,12(5*H*,11*H*)-dione (**10c**, 376 mg, 1 mmol, 1 equiv.) was dissolved in dry THF and then *p*-tolyl Davy-reagent (873 mg, 2 mmol, 2 equiv.) was added. The resulting suspension was refluxed for 18 h, then the reaction mixture was cooled to room temperature. The obtained clear, yellow solution was evaporated with small amount of chromatographic silica gel. The crude product was purified by column chromatography using hexane/EtOAc 9:1 then 7:3 *v/v* as eluent. Yield 58% (237 mg), yellow solid, m.p. 186–187 °C (decomposition), R.f. = 0.27 (hexane:ethyl acetate 7:3 *v*/*v*). ^1^H-NMR (DMSO-*d*_6_) δ 12.30 (s, 1H, NH), 12.28 (s, 1H, NH), 7.56–7.49 (m, 2H, H_Ar_), 6.99 (d, 1H, *J* = 5.1 Hz, H_Ar_), 6.88 (s, 1H, H_Ar_), 6.64 (s, 1H, H_Ar_), 3.73 (s, 3H, OMe), 3.70 (s, 3H, OMe); ^13^C-NMR (DMSO-*d*_6_) δ 200.2, 197.5, 149.8, 148.0, 141.2, 133.1, 132.9, 131.2, 131.1, 126.9, 126.6, 120.4, 111.4, 107.7, 55.9, 55.7; IR (KBr): cm^−1^ 3439, 3248, 2925, 2644, 1602, 1506, 1403, 1345, 1265, 1227,1200, 1141, 1079, 1030; HRMS (ESI): *m/z* [M+H]^+^ calcd for C_16_H_14_BrN_2_O_2_S_2_: 408.96746, 410.96541, found: 408.96725, 410.96512;

#### 3.1.9. General Procedure for the Synthesis of 1-Substituted 1*H*-benzo[*d*][1,3]oxazine-2,4-diones **13f–i**

To a stirred solution of 6-chloro-1*H*-benzo[*d*][1,3]oxazine-2,4-dione (**13a**), 6-bromo-1*H*- benzo[*d*][1,3]oxazine-2,4-dione (**13b**) or 1*H*-benzo[*d*][1,3]oxazine-2,4-dione (**13c**) (1 equiv.) in DMSO (10 mL/mmol), 60% sodium hydride (1.5 equiv.) was added and the resulting suspension was stirred for 15 min. at room temperature, until the evolution of gas ceased. Then, the appropriate halide (1.5 equiv.): methyl iodide for **13a**, 1-(bromomethyl)naphthalene for **13b** or benzyl bromide and 4-bromobenzyl bromide for **13c**, was added and the resulting mixture was stirred at room temperature for 18 h. Next day, the reaction mixture was poured into water (100 mL/10 mL DMSO) and extracted with ethyl acetate (3 × 100 mL/100 mL H_2_O). Combined organic layers were washed with brine (100 mL/300 mL EtOAc) and dried over anhydrous magnesium sulfate. Crude products were crystallized from mixture of ethyl acetate and hexane.

*6-Chloro-1-methyl-1H-benzo[d][1,3]oxazine-2,4-dione* (**13f**): Yield 62%, yellow crystals, m.p. 201–202 °C, R.f. = 0.33 (hexane:ethyl acetate 7:3). ^1^H-NMR (DMSO-*d*_6_) δ 7.94 (d, 1H, *J* = 2.4 Hz, H_Ar_), 7.89 (dd, 1H, *J* = 2.4, 9.0 Hz, H_Ar_), 7.48 (d, 1H, *J* = 9.0 Hz, H_Ar_), 3.46 (s, 1H, Me); ^13^C-NMR (DMSO-*d*_6_) δ 158.0, 147.4, 141.1, 136.6, 127.9, 127.6, 117.1, 113.3, 31.9; HRMS (ESI): *m/z* [M+H]^+^ calcd for C_9_H_7_ClNO_3_: 212.01090, 214.00795, found: 212.01049, 214.00754;

*1-Benzyl-1H-benzo[d][1,3]oxazine-2,4-dione* (**13g**): Yield 57%, beige crystals, m.p. 141–142 °C, R.f. = 0.54 (hexane:ethyl acetate 7:3). ^1^H-NMR (DMSO-*d*_6_) δ 8.04 (dd, 1H, *J* = 1.5, 7.8 Hz, H_Ar_), 7.73 (ddd, 1H, *J* = 1.5, 7.2, 8.7 Hz, H_Ar_), 7.46–7.20 (m, 7H, H_Ar_), 5.30 (s, 2H, CH_2_); ^13^C-NMR (DMSO-*d*_6_) δ 158.9, 148.3, 141.3, 137.0, 135.3, 129.5, 128.6, 127.4, 126.6, 123.7, 115.1, 112.1, 47.6; HRMS (ESI): *m/z* [M+H]^+^ calcd for C_15_H_12_NO_3_: 254.08116, found: 254.08104;

*1-(4-Bromobenzyl)-1H-benzo[d][1,3]oxazine-2,4-dione* (**13h**): Yield 62%, beige crystals, m.p. 182–183 °C, R.f. = 0.50 (hexane:ethyl acetate 7:3). ^1^H-NMR (DMSO-*d*_6_) δ 8.04 (dd, 1H, *J* = 1.5, 7.8 Hz, H_Ar_), 7.74 (ddd, 1H, *J* = 1.5, 7.2, 8.7 Hz, H_Ar_), 7.85–7.49 (m, 2H, H_Ar_), 7.44–7.36 (m, 2H, H_Ar_), 7.35–7.26 (m, 1H, H_Ar_), 7.22 (dd, 1H, *J* = 8.4 Hz, H_Ar_), 5.27 (s, 2H, CH_2_); ^13^C-NMR (DMSO-*d*_6_) δ 158.8, 148.3, 141.2, 137.0, 134.8, 131.5, 129.5, 129.0, 123.8, 120.5, 115.0, 112.2, 47.0; HRMS (ESI): *m/z* [M+H]^+^ calcd for C_15_H_11_BrNO_3_: 331.99168, 333.98964, found: 331.99138, 333.98930;

*6-Bromo-1-(naphthalen-1-ylmethyl)-1H-benzo[d][1,3]oxazine-2,4-dione* (**13i**): Yield 51%, white solid, m.p. 230–231 °C, R.f. = 0.74 (hexane:ethyl acetate 7:3). ^1^H-NMR (DMSO-*d*_6_) δ 8.26–8.16 (m, 1H, H_Ar_), 8.15 (d, 1H, *J* = 2.4 Hz, H_Ar_), 8.04–7.97 (m, 1H, H_Ar_), 7.92–7.78 (m, 2H, H_Ar_), 7.72–7.56 (m, 2H, H_Ar_), 7.42–7.33 (m, 2H, H_Ar_), 7.02 (d, 1H, *J* = 9.0 Hz, H_Ar_), 5.73 (s, 2H, CH_2_); ^13^C-NMR (DMSO-*d*_6_) δ 157.9, 147.8, 140.8, 139.2, 133.3, 131.0, 129.9, 129.5, 128.7, 127.7, 126.5, 126.2, 125.4, 123.0, 122.2, 117.7, 115.3, 114.52, 114.48, 46.4; HRMS (ESI): *m/z* [M+H]^+^ calcd for C_19_H_13_BrNO_3_: 382.0073, 384.00529 found: 382.0645, 384.00443;

#### 3.1.10. DCC-mediated Synthesis of 2-(2-aminophenyl)-4*H*-benzo[*d*][1,3]oxazin-4-one (**12**, R^1^ = R^2^ = H)

A suspension of 1*H*-benzo[*d*][1,3]oxazine-2,4-dione (**13c**, 489 mg, 3 mmol, 1 equiv.), 2-aminobenzoic acid (**14a**, 411 mg, 3 mmol, 1 equiv.), sodium hydroxide (120 mg, 3 mmol, 1 equiv.) in water (30 mL) was heated at 80 °C for 30 min. until the evolution of carbon dioxide ceased and clear solution was formed. After cooling, the obtained solution was diluted with water, crude product was precipitated by addition of glacial acetic acid and the resulting precipitate was dried under reduced pressure. The crude 2-(2-aminobenzamido)benzoic acid (**11**) was dissolved in 50 mL of DMF, then *N*,*N*’-dicyclohexylcarbodiimide (DCC) (681 mg, 3.3 mmol, 1.1 equiv.) was added and the reaction mixture was stirred at room temperature for 18 h. The next day, the obtained solution was poured into water (100 mL) and the product was extracted with ethyl acetate (3 × 100 mL). Combined organic layers were washed with brine (1 × 50 mL), dried over anhydrous magnesium sulfate followed by evaporation of volatiles under reduced pressure. Crude product was purified by column chromatography using hexane: ethyl acetate 9:1 *v/v* as eluent. yield 84% (600 mg), yellow solid, m.p. 171–172 °C, R.f. = 0.69 (hexane:ethyl acetate 7:3). ^1^H-NMR (CDCl_3_) δ 8.19 (d, 1H, *J* = 4.8 Hz, H_Ar_), 8.08 (d, 1H, *J* = 4.8 Hz, H_Ar_), 7.76 (t, 1H, *J* = 4.7 Hz, H_Ar_), 7.56 (d, 1H, *J* = 5.1 Hz, H_Ar_), 7.44 (t, 1H, *J* = 4.5 Hz, H_Ar_), 7.26 (t, 1H, *J* = 4.7 Hz, H_Ar_), 6.78–6.68 (m, 2H, H_Ar_), 6.47 (bs, 2H, NH_2_); ^13^C-NMR (CDCl_3_) δ 159.4, 158.0, 149.8, 146.7, 136.5, 133.7, 129.7, 128.7, 127.8, 126.4, 116.9, 116.8, 116.6, 110.1; IR (KBr): cm^−1^ 3448, 3311, 1743, 1625, 1592, 1550, 1489, 1471, 1447, 1356, 1331, 1310, 1235, 1220, 1167, 1054, 1013; HRMS (ESI): *m/z* [M+H]^+^ calcd for C_14_H_11_N_2_O_2_: 239.08150, found: 239.08128;

#### 3.1.11. The Synthesis of methyl 2-amino-5-chlorobenzoate (**20**)

To stirred slurry of 6-chloro-1*H*-benzo[*d*][1,3]oxazine-2,4-dione (**13a**, 591 mg, 3 mmol, 1 equiv.) in methanol (30 mL), sodium methoxide (486 mg, 9 mmol, 3 equiv.) was added, and the reaction mixture was refluxed for 3 h. After cooling down, the excess of solvent was evaporated under reduced pressure; the obtained residue was treated with water (100 mL) and extracted with ethyl acetate (3 × 50 mL). Combined organic layers were washed with water (1 × 100 mL), brine (1 × 50 mL) and dried over anhydrous magnesium sulfate. Evaporation of solvent gave pure product as slight yellow oil which solidified upon storage. Yield 97% (538 mg), m.p. 70–71 °C, R.f. = 0.80 (hexane:ethyl acetate 7:3). ^1^H-NMR (CDCl_3_) δ 7.81 (d, 1H, *J* = 2.4 Hz, H_Ar_), 7.19 (dd, 1H, *J* = 2.4, 9.0 Hz, H_Ar_), 6.59 (d, 1H, *J* = 9.0 Hz, H_Ar_), 5.73 (bs, 2H, NH_2_), 3.86 (s, 3H, Me); ^13^C-NMR (CDCl_3_) δ 167.7, 149.1, 134.2, 130.5, 120.7, 118.1, 111.6, 51.9; HRMS (ESI): *m/z* [M+H]^+^ calcd for C_8_H_9_ClNO_2_: 186.03163, 188.02868 found: 186.03171, 188.02870.

### 3.2. X-ray Data Collection and Data Refinement

Good quality single-crystals of **10b**, **10g**, **10h**, **10i**, **10j**, **10l**, **10m**, 10o 11*TsOH, **13i** and **14c**, were selected for the X-ray diffraction experiments at *T* = 100(2) K. Diffraction data were collected on a SuperNova Dual Source diffractometer (Agilent Technologies, Yarnton, Oxfordshire, UK) with Cu*K*α radiation (*λ* = 1.54184 Å) (**10g**, **10h**, **10i**, **10j**, **10l**, **10m**, 10o 11*TsOH, **13i** and **14c**) or an Agilent Technologies SuperNova Single Source diffractometer with Mo*K*α radiation (*λ* = 0.71073 Å) (**10b**), using CrysAlis RED software [17]. The analytical numeric absorption correction using a multifaceted crystal model based on expressions derived by Clark and Reid [18] (**10b**, **10l**, 10j 10h, 11*TsOH and **13i**) and multi-scan empirical absorption correction using spherical harmonics (**10i**, **10g**, **10m**, **10o** and **14c**), implemented in SCALE3 ABSPACK scaling algorithm, were applied [17]. The structural determination procedure was carried out using the SHELX package [19]. The structures were solved with direct methods and then successive least-square refinement was carried out based on the full-matrix least-squares method on *F*^2^ using the SHELXL programme [19]. All H-atoms linked to the N and O-atoms were located on a Fourier difference map and refined as riding with *U*_iso_(H) = x*U*_eq_(N,O), where x = 1.2 for the amine and 1.5 for the hydroxyl H-atoms, respectively. In all the cases, the N−H bonds were subject to the DFIX 0.87 restraint. In the case of **14c,** length of the O−H bond was restrained to 0.82 Å. Other H-atoms were positioned geometrically, with C–H equal to 0.93, 0.97 and 0.98 Å for the aromatic, methylene and methine H-atoms, respectively, and constrained to ride on their parent atoms with U_iso_(H) = x*U*_eq_(C), where x = 1.2 for the aromatic, methylene and methine H-atoms. In the case of **10i**, a few distinct peaks on the difference Fourier map indicated the presence of a disordered solvent molecule. However, all of the attempts to model disordered solvents used for crystallization failed. Therefore, the solvent contribution was removed by applying the appropriate MASK procedure in the Olex2 programme [20]. Calculated total solvent accessible volume was 982.1 Å^3^ occupied by 286.6 electrons per unit cell. The figures for this publication were prepared using Olex2 programme [20].

### 3.3. Cell Culturing

Following cell lines: HeLa, U87, HEK293, EUFA30 were cultured in DMEM medium (Life Technology, Carlsbad, CA, USA) with 10% fetal bovine serum (Thermo Fisher Scientific, Waltham, MA, USA) and 0.1% antibiotics (penicillin, streptomycin, Life Technology, Carlsbad, CA, USA). Cells were grown in atmosphere of 5 and 95% CO_2_ and air, respectively, at 37 °C.

### 3.4. Cytotoxicity Assay

Exponentially growing cells at the density of 2 × 10^3^ cells/well were seeded onto a 96-well plate, cultured for 18 h, and treated with newly synthesized compounds at concentrations 1–200 µM, or with DMSO as a control, for 24 or 48 h. Alamar Blue (Thermo Fisher Scientific, Waltham, MA, USA) was added accordingly to manufacturer protocol. After 4 h, light emission at 590 nm was measured with excitation at 560 nm using a scanning multiwell spectrophotometer (DTX 880, Beckman Coulter, Brea, CA, USA). The experiments were repeated at least three times with three replicates for each inhibitor concentration. After background subtraction, inhibition rates, IC_50_, were calculated as the component concentration inhibiting cell growth by 50%. All the calculations were performed using Origin 9.0 software.

### 3.5. Flow Cytometry

The apoptosis detection kit (Annexin V-FITC BD Biosciences, Franklin Lakes, NJ, USA) was used to detect apoptosis by flow cytometry. Cells were seeded in 6-well plates at concentration of 5 × 105 cells/well, cultured for 18 h, and tested compound was applied for indicated time. Afterwards, cells were washed with PBS, resuspended in binding buffer at concentration of 2 × 106 cells/mL. The anti-Annexin V FITC-conjugated antibody and propidium iodide were added to 100 µl aliquots. Then, mixtures were incubated for 15 min at room temperature, supplemented with binding buffer to 500 µl and processed by BD FACSCalibur (BD Biosciences, Franklin Lakes, NJ, USA). Data were analyzed in Flowing Software version 2.5.1 (Flowing Software, http://www.uskonaskel.fi/flowingsoftware).

### 3.6. Disk Diffusion Test

Bacterial inoculum was spread on LB plates solidified with 1.5% agar. Standard 6 mm paper discs were placed on the surface of the plates and 4 µL of tested compound at the desired concentration plotted on the discs.

## 4. Conclusions

In this article we have presented that asymmetrically substituted dibenzo[*b*,*f*][1,5]- diazocine-6,12(5*H*,11*H*)diones, which may be treated as convenient privileged structures useful in the design of biologically active compounds, can be obtained according to two, general methods based on reactions of: (a) 1*H*-benzo[*d*][1,3]oxazine-2,4-diones and unprotected 2-aminobenzoic acids, and (b) unprotected 2-aminobenzoic acid analogues, activated with thionyl chloride, and 2-aminobenzoic acid esters. The procedures described allow a wide range of modifications of all structural elements of the dibenzo[*b*,*f*][1,5]diazocine-6,12(5*H*,11*H*)dione framework: the introduction of various substituents and functional groups into benzene rings as well as diverse substituents and side chains attached to the eight-membered diazocine ring. We have shown that these modifications can be introduced both, through the selection of appropriately modified building blocks as well as further chemical modifications of the obtained dibenzo[*b*,*f*][1,5]diazocine-6,12(5*H*,11*H*)dione scaffold. The wide range of synthetic methods has also allowed to obtain three new heterocyclic frameworks: benzo[*b*]naphtho[2,3-*f*][1,5]diazocine-6,14(5*H*,13*H*)-dione **10h**, pyrido[3,2-*c*][1,5]benzo- diazocine-5,11(6*H*,12*H*)-dione **10l**, and pyrazino[3,2-*c*][1,5]benzodiazocine-6,12(5*H*,11*H*)-dione **10m**. The usefulness of the synthetic methods has been confirmed by obtaining a representative library of 16 compounds, evaluated as possible cytotoxic and antimicrobial agents.

## Figures and Tables

**Table 1:** Synthesized compounds.

Substrate 1	Substrate 2	Product	Substrate 1	Substrate 2	Product
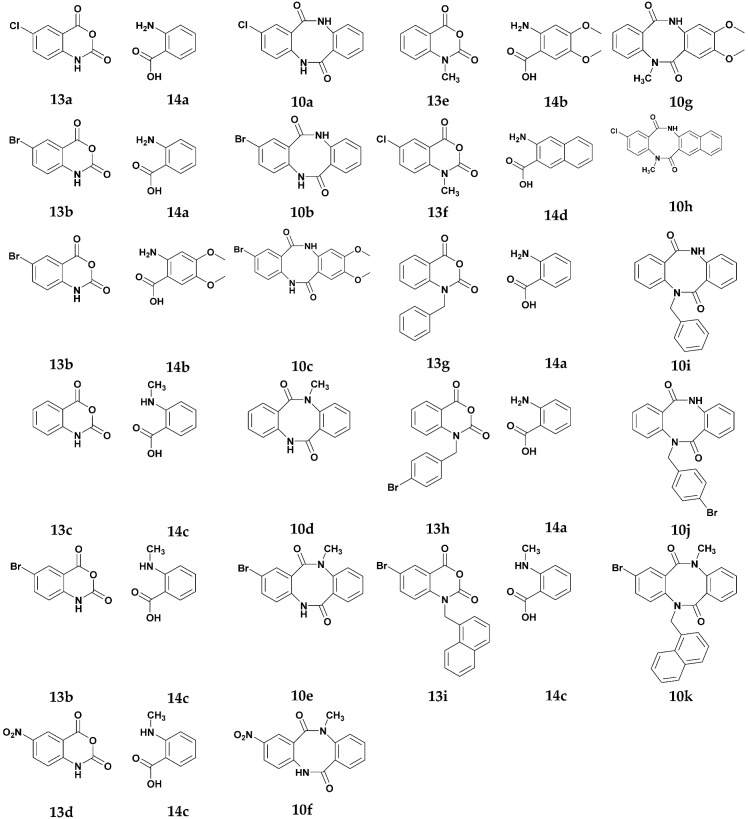					

**Table 2:** IC_50_ (μM) of dibenzo[*b*,*f*][1,5]diazocine-6,12-diones **10a–p** based on the survival of non-cancerous (HEK293, EUFA30) and cancerous (U87, HeLa) cells after 24 and 48 h of treatment. “*”-Proliferation inhibition at 200 µM, the highest concentration used.

Compound	Cell Types							
	Eufa30		HEK293		HeLa		U87	
	24 h	48 h	24 h	48 h	24 h	48 h	24 h	48 h
**10a**	[20.7]* ± 2.4	[17.7]* ± 11.2	[19.2]* ± 2.5	[9.7]* ± 5.2	[12.2]* ± 0.1	[0.7]* ± 0.1	[18.9]* ± 9.1	[7.2]* ± 0.6
**10b**	[27.0]* ± 15.7	[21.8]* ± 2.3	[18.5]* ± 7.2	[18.2]* ± 4.5	**97.3** ± 13.7	[12.7]* ± 3.6	[27.0]* ± 7.7	[15.9]* ± 4.5
**10c**	[41.1]* ± 8.3	[0.0]*	[14.9]* ± 5.6	[9.3]* ± 5.5	[31.4]* ± 10.0	[0.8]* ± 2.5	[20.8]* ± 4.2	[0.0]*
**10d**	[17.0]* ± 27.7	[16.5]* ± 3.0	[12.9]* ± 8.2	[0.0]*	[34.3]* ± 8.3	[9.2]* ± 4.8	[11.5]* ± 3.4	[2.5]* ± 1.4
**10e**	[9.8]* ± 6.2	[12.2]* ± 2.7	[24.3]* ± 0.4	[11.2]* ± 1.7	[30.7]* ± 9.0	[0.9]* ± 2.4	[24.4]* ± 3.1	[9.4]* ± 2.4
**10f**	[7.7]* ± 6.4	[13.2]* ± 4.1	[4.7]* ± 3.1	[0.7]* ± 4.1	**205.7** ± 12.8	[5.2]* ± 1.9	[30.9]* ± 0.3	[3.6]* ± 2.9
**10g**	[41.5]* ± 1.4	[5.4]* ± 1.3	[9.7]* ± 3.3	[7.2]* ± 4.2	[21.0]* ± 4.4	[6.1]* ± 2.0	[22.6]* ± 3.1	[0.0]*
**10h**	**170.5** ± 21.1	**199.8** ± 13.9	[37.0]* ± 18.9	[33.7]* ± 10.0	**107.4** ± 10.0	**107.6** ± 8.6	**148.6** ± 9.0	**181.9** ± 5.6
**10i**	[20.3]* ± 1.8	[20.1]* ± 5.5	[24.7]* ± 5.8	[25.2]* ± 2.2	[31.8]* ± 5.2	[5.3]* ± 2.3	[27.3]* ± 6.4	[5.5]* ± 0.8
**10j**	[36.4]* ± 11.4	[38.2]* ± 2.7	[30.8] ± 5.4	**192.0** ± 12.2	**137.5** ± 14.1	**202.6** ± 4.0	[41.8]* ± 10.8	[42.4]* ± 5.7
**10k**	**87.6** ± 8.1	**74.4** ± 6.7	**119.0** ± 7.1	**138.3** ± 12.0	**75.3** ± 1.6	**123.9** ± 5.4	**75.4** ± 8.7	**197.4** ± 9.4
**10l**	[12.8]* ± 3.5	[9.9]* ± 2.5	[12.9]* ± 2.1	[5.9]* ± 2.4	[23.2]* ± 10.3	[0.0]*	[24.2]* ± 2.8	[4.9]* ± 2.2
**10m**	[24.0]* ± 10.1	[6.3]* ± 4.8	[7.9]* ± 4.0	[5.6]* ± 2.0	[39.1]* ± 5.2	[5.0]* ± 2.3	[20.5]* ± 4.2	[9.9]* ± 2.9
**10n**	[8.2] ± 1.2	[0.0]*	[0.0]*	[0.0]*	[3.1]* ± 2.2	[0.0]*	[9.9]* ± 0.8	[0.0]*
**10o**	[23.3]* ± 9.5	[25.2]* ± 5.7	[18.3]* ± 7.3	[12.9]* ± 7.3	[32.9]* ± 3.3	[5.9]* ± 1.5	[23.9]* ± 4.8	[0.0]*
**10p**	[33.4]* ± 2.1	**157.6** ± 9.6	**202** ± 7.9	**144.7** ± 8.5	**115.2** ± 4.5	**203.9** ± 6.5	**172.0** ± 10.0	[3.8]* ± 0.6

## Supplementary Materials

^1^H-NMR, ^13^C-NMR, IR, HRMS (ESI) and X-Ray analysis data are available online. CCDC 1956772−19567781 and 1976509 contain the supplementary crystallographic data for this paper. These data can be obtained freely via http://www.ccdc.cam.ac.uk/data_request/cif, by e-mailing data_request@ccdc.cam.ac.uk, or by contacting directly the Cambridge Crystallographic Data Centre (12 Union Road, Cambridge CB2 1EZ, UK. Fax: +44-1223-336033).
