# Supplementary material for: Unsymmetrically Substituted Dibenzo[b,f][1,5]-diazocine-6,12(5H,11H)dione—A Convenient Scaffold for Bioactive Molecule Design
Source: Molecules. 2020 Feb 18;25(4):906. doi: 10.3390/molecules25040906 (PMC7070320; doi:10.3390/molecules25040906)
Supplement: Supplementary file 1 [file molecules-25-00906-s001.pdf]

Unsymmetrically Substituted Dibenzo[*b*,*f*][1,5]diazocine-6,12(5*H*,11*H*)dione—A Convenient Scaffold for Bioactive Molecule Design

Bartosz Bieszczad [1,*], Damian Garbicz [1], Damian Trzybiński [2], Damian Mielecki [1], Krzysztof Woźniak [2], Elżbieta Grzesiuk [1] and Adam Mieczkowski [1,*]

1 Institute of Biochemistry and Biophysics, Polish Academy of Sciences, Pawińskiego 5a, 02-106 Warsaw, Poland; dgarbicz@ibb.waw.pl (D.G.); damian@ibb.waw.pl (D.M.); elag@ibb.waw.pl (E.G.)

2 Biological and Chemical Research Centre, University of Warsaw, Żwirki i Wigury 101, 02-089 Warsaw, Poland; dtrzybinski@cnbc.uw.edu.pl (D.T.); kwozniak@chem.uw.edu.pl (K.W.)

* Correspondence: b.bieszczad@ibb.waw.pl (B.B.); amiecz@ibb.waw.pl (A.M.)

SUPPORTING INFORMATION

crystallographic data, 1H NMR, 13C NMR, IR, HRMS spectra

Table 1S. Crystal data and structure refinement for investigated compounds.

Identification code	**11*TsOH**	**14c**	**10b**	**10i**	**10g**	**10l**	**10j**	**10m**	**10h**	**10o**
Empirical formula	$C_{21}H_{20}N_2O_6S$	$C_8H_9NO_2$	$C_{17}H_{15}BrN_2O_3$	$C_{21}H_{16}N_2O_2$	$C_{17}H_{16}N_2O_4$	$C_{13}H_8ClN_3O_2$	$C_{21}H_{15}BrN_2O_2$	$C_{12}H_7ClN_4O_2$	$C_{19}H_{13}ClN_2O_2$	$C_{17}H_{14}N_2O_3$
Formula weight	428.45	151.16	375.22	328.36	312.32	273.67	407.26	274.67	336.76	294.30
Temperature/K	100(2)	100(2)	100(2)	100(2)	100(2)	100(2)	100(2)	100(2)	100(2)	100(2)
Crystal system	monoclinic	monoclinic	monoclinic	monoclinic	monoclinic	monoclinic	triclinic	monoclinic	orthorhombic	orthorhombic
Space group	$P2_1/c$	$P2_1$/c	$P2_1$/c	$C2/c$	$P2_1/c$	$P2_1/c$	P-1	$P2_1/c$	$P2_12_12_1$	$Pbca$
a/Å	12.9091(10)	7.7035(2)	16.8608(2)	21.8373(11)	9.4656(2)	10.1324(17)	7.1046(4)	9.4425(3)	8.9824(4)	13.0878(3)
b/Å	5.7241(4)	14.8365(3)	8.86324(15)	11.0973(3)	8.66921(19)	9.0970(11)	8.6754(6)	10.1523(3)	9.3332(4)	13.2936(5)
c/Å	26.3339(19)	6.9063(2)	11.27530(18)	20.8549(10)	19.1381(5)	12.894(2)	15.3501(9)	11.8341(3)	18.6008(11)	16.1239(5)
α/°	90	90	90	90	90	90	90.207(5)	90	90	90
β/°	97.997(7)	112.862(4)	100.2887(15)	127.977(8)	100.184(2)	109.542(18)	100.295(5)	90.452(3)	90	90
γ/°	90	90	90	90	90	90	109.826(6)	90	90	90
Volume/Å^3	1927.0(2)	727.33(4)	1657.91(5)	3983.7(4)	1545.72(6)	1120.1(3)	873.59(10)	1134.41(6)	1559.40(13)	2805.31(15)
Z	4	4	4	8	4	4	2	4	4	8
ρ_{calc}g/cm^3	1.477	1.380	1.503	1.095	1.342	1.623	1.548	1.608	1.434	1.394
μ/mm^{-1}	1.877	0.828	2.495	0.573	0.802	3.050	3.354	3.041	2.286	0.797
F(000)	896.0	320.0	760.0	1376.0	656.0	560.0	412.0	560.0	696.0	1232.0
Crystal size/mm^3	0.33 × 0.10 × 0.05	0.57 × 0.28 × 0.18	0.37 × 0.19 × 0.13	0.43 × 0.17 × 0.10	0.33 × 0.18 × 0.16	0.17 × 0.13 × 0.03	0.26 × 0.09 × 0.04	0.39 × 0.24 × 0.06	0.15 × 0.12 × 0.05	0.35 × 0.22 × 0.09
Radiation	Cu$K\alpha$ (λ = 1.54184)	Cu$K\alpha$ (λ = 1.54184)	Mo$K\alpha$ (λ = 0.71073)	Cu$K\alpha$ (λ = 1.54184)	Cu$K\alpha$ (λ = 1.54184)	Cu$K\alpha$ (λ = 1.54184)	Cu$K\alpha$ (λ = 1.54184)	Cu$K\alpha$ (λ = 1.54184)	Cu$K\alpha$ (λ = 1.54184)	Cu$K\alpha$ (λ = 1.54184)
2Θ range for data collection/°	6.78 to 134.108	11.93 to 134.12	4.91 to 52.738	9.21 to 134.142	9.39 to 134.146	9.262 to 134.108	5.866 to 134.152	9.366 to 134.146	9.51 to 134.118	10.96 to 134.102
Index ranges	$-14 \le h \le 15$, $-6 \le k \le 4$, $-31 \le l \le 30$	$-9 \le h \le 9$, $-17 \le k \le 17$, $-8 \le l \le 8$	$-21 \le h \le 21$, $-11 \le k \le 11$, $-14 \le l \le 14$	$-26 \le h \le 24$, $-13 \le k \le 13$, $-24 \le l \le 21$	$-11 \le h \le 11$, $-10 \le k \le 10$, $-22 \le l \le 22$	$-12 \le h \le 11$, $-10 \le k \le 5$, $-14 \le l \le 15$	$-8 \le h \le 7$, $-10 \le k \le 9$, $-18 \le l \le 18$	$-10 \le h \le 11$, $-12 \le k \le 6$, $-14 \le l \le 14$	$-10 \le h \le 10$, $-8 \le k \le 11$, $-22 \le l \le 21$	$-11 \le h \le 15$, $-15 \le k \le 15$, $-19 \le l \le 19$
Reflections collected	6178	4115	41718	13274	22410	3695	6882	3759	5735	18308
Independent reflections	3437 [R_{int} = 0.0345, R_{sigma} = 0.0500]	1301 [R_{int} = 0.0150, R_{sigma} = 0.0128]	3378 [R_{int} = 0.0338, R_{sigma} = 0.0133]	3565 [R_{int} = 0.0270, R_{sigma} = 0.0234]	2767 [R_{int} = 0.0302, R_{sigma} = 0.0131]	1989 [R_{int} = 0.0395, R_{sigma} = 0.0670]	3115 [R_{int} = 0.0416, R_{sigma} = 0.0499]	2023 [R_{int} = 0.0168, R_{sigma} = 0.0219]	2772 [R_{int} = 0.0343, R_{sigma} = 0.0510]	2500 [R_{int} = 0.0309, R_{sigma} = 0.0152]
Data/restraints/parameters	3437/8/288	1301/2/107	3378/2/216	3565/2/229	2767/1/214	1989/2/178	3115/1/238	2023/2/178	2772/1/221	2500/0/201
Goodness-of-fit on F^2	1.031	1.046	1.049	1.071	1.046	1.023	1.040	1.057	1.061	1.058
Final R indexes [I>=2σ (I)]	R_1 = 0.0468, wR_2 = 0.1104	R_1 = 0.0310, wR_2 = 0.0857	R_1 = 0.0232, wR_2 = 0.0575	R_1 = 0.0384, wR_2 = 0.1013	R_1 = 0.0378, wR_2 = 0.0984	R_1 = 0.0467, wR_2 = 0.1144	R_1 = 0.0390, wR_2 = 0.0873	R_1 = 0.0306, wR_2 = 0.0808	R_1 = 0.0433, wR_2 = 0.1039	R_1 = 0.0358, wR_2 = 0.0907
Final R indexes [all data]	R_1 = 0.0662, wR_2 = 0.1238	R_1 = 0.0325, wR_2 = 0.0872	R_1 = 0.0266, wR_2 = 0.0592	R_1 = 0.0443, wR_2 = 0.1055	R_1 = 0.0416, wR_2 = 0.1025	R_1 = 0.0650, wR_2 = 0.1289	R_1 = 0.0518, wR_2 = 0.0973	R_1 = 0.0328, wR_2 = 0.0829	R_1 = 0.0494, wR_2 = 0.1072	R_1 = 0.0400, wR_2 = 0.0954
Largest diff. peak/hole / e Å^{-3}	0.38/-0.28	0.22/-0.20	0.43/-0.46	0.16/-0.25	0.21/-0.32	0.36/-0.30	0.37/-0.63	0.24/-0.24	0.28/-0.23	0.17/-0.21
Flack parameter	–	–	–	–	–	–	–	–	0.445(14)	–
CCDC number	1956777	1956781	1956778	1956773	1956779	1956775	1956772	1956774	1956776	1956790

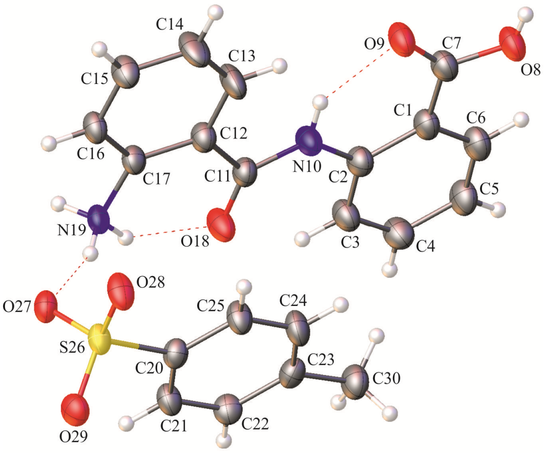

Figure 1S. Asymmetric unit of the crystal lattice of **11*TsOH** with crystallographic atom numbering. Displacement ellipsoids are drawn at the 50% probability level. The H-atoms are shown as small spheres of arbitrary radius. The intermolecular N−H···O hydrogen bonds are represented by a red dashed lines.

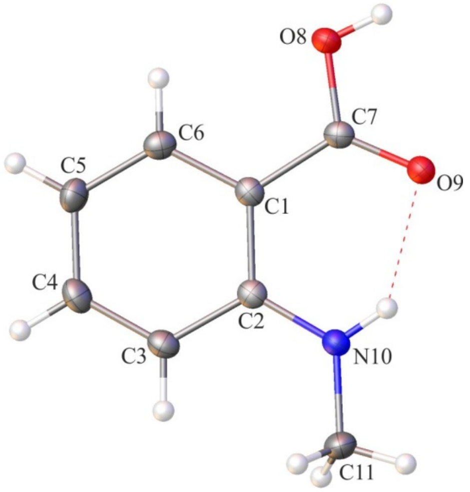

Figure 2S. Asymmetric unit of the crystal lattice of **14c** with crystallographic atom numbering. Displacement ellipsoids are drawn at the 50% probability level. The H-atoms are shown as small spheres of arbitrary radius. The intramolecular N−H···O hydrogen bond is represented by a red dashed lines.

Table 2S. Bond lengths for **11*TsOH**.

Atom	Atom	Length/Å	Atom	Atom	Length/Å
C(1)	C(2)	1.414(4)	C(14)	C(15)	1.380(4)
C(1)	C(6)	1.397(4)	C(15)	C(16)	1.381(4)
C(1)	C(7)	1.488(4)	C(16)	C(17)	1.383(4)
C(2)	C(3)	1.395(4)	C(17)	N(19)	1.468(3)
C(2)	N(10)	1.409(4)	C(20)	C(21)	1.391(4)
C(3)	C(4)	1.393(4)	C(20)	C(25)	1.386(4)
C(4)	C(5)	1.378(4)	C(20)	S(26)	1.772(3)
C(5)	C(6)	1.372(4)	C(21)	C(22)	1.392(4)
C(7)	O(8)	1.328(3)	C(22)	C(23)	1.388(4)
C(7)	O(9)	1.229(4)	C(23)	C(24)	1.388(4)
C(11)	C(12)	1.507(4)	C(23)	C(30)	1.506(4)
C(11)	N(10)	1.358(3)	C(24)	C(25)	1.400(4)
C(11)	O(18)	1.230(3)	O(27)	S(26)	1.4765(19)
C(12)	C(13)	1.397(4)	O(28)	S(26)	1.4404(19)
C(12)	C(17)	1.398(4)	O(29)	S(26)	1.465(2)

C(13)	C(14)	1.372(4)

Table 3S. Values of valence angles for **11*TsOH**.

Atom	Atom	Atom	Angle/°	Atom	Atom	Atom	Angle/°
C(2)	C(1)	C(7)	122.3(2)	C(15)	C(16)	C(17)	119.6(2)
C(6)	C(1)	C(2)	118.7(3)	C(12)	C(17)	N(19)	121.7(2)
C(6)	C(1)	C(7)	118.9(3)	C(16)	C(17)	C(12)	121.8(2)
C(3)	C(2)	C(1)	119.7(3)	C(16)	C(17)	N(19)	116.5(2)
C(3)	C(2)	N(10)	122.1(3)	C(11)	N(10)	C(2)	129.0(2)
N(10)	C(2)	C(1)	118.2(3)	C(21)	C(20)	S(26)	119.1(2)
C(4)	C(3)	C(2)	119.7(3)	C(25)	C(20)	C(21)	120.7(2)
C(5)	C(4)	C(3)	120.7(3)	C(25)	C(20)	S(26)	120.2(2)
C(6)	C(5)	C(4)	120.0(3)	C(20)	C(21)	C(22)	119.6(3)
C(5)	C(6)	C(1)	121.2(3)	C(23)	C(22)	C(21)	121.0(3)
O(8)	C(7)	C(1)	113.2(2)	C(22)	C(23)	C(30)	121.1(3)
O(9)	C(7)	C(1)	124.6(3)	C(24)	C(23)	C(22)	118.2(3)
O(9)	C(7)	O(8)	122.2(3)	C(24)	C(23)	C(30)	120.7(3)
N(10)	C(11)	C(12)	115.6(2)	C(23)	C(24)	C(25)	122.0(3)
O(18)	C(11)	C(12)	121.0(2)	C(20)	C(25)	C(24)	118.4(3)
O(18)	C(11)	N(10)	123.4(2)	O(27)	S(26)	C(20)	103.98(11)
C(13)	C(12)	C(11)	121.7(2)	O(28)	S(26)	C(20)	107.94(12)
C(13)	C(12)	C(17)	116.9(2)	O(28)	S(26)	O(27)	112.78(12)
C(17)	C(12)	C(11)	121.3(2)	O(28)	S(26)	O(29)	113.66(13)
C(14)	C(13)	C(12)	121.5(3)	O(29)	S(26)	C(20)	107.32(12)
C(13)	C(14)	C(15)	120.5(3)	O(29)	S(26)	O(27)	110.51(11)
C(14)	C(15)	C(16)	119.6(3)				

Table 4S. Values of torsion angles for **11*TsOH**.

A	B	C	D	Angle/°	A	B	C	D	Angle/°
C(1)	C(2)	C(3)	C(4)	1.1(5)	C(15)	C(16)	C(17)	C(12)	0.0(4)
C(1)	C(2)	N(10)	C(11)	-173.4(3)	C(15)	C(16)	C(17)	N(19)	179.1(3)
C(2)	C(1)	C(6)	C(5)	-0.4(5)	C(17)	C(12)	C(13)	C(14)	-1.2(5)
C(2)	C(1)	C(7)	O(8)	172.4(3)	N(10)	C(2)	C(3)	C(4)	179.7(3)
C(2)	C(1)	C(7)	O(9)	-7.7(5)	N(10)	C(11)	C(12)	C(13)	-6.4(4)
C(2)	C(3)	C(4)	C(5)	-1.1(5)	N(10)	C(11)	C(12)	C(17)	173.9(3)
C(3)	C(2)	N(10)	C(11)	8.0(5)	O(18)	C(11)	C(12)	C(13)	173.5(3)
C(3)	C(4)	C(5)	C(6)	0.3(5)	O(18)	C(11)	C(12)	C(17)	-6.2(4)
C(4)	C(5)	C(6)	C(1)	0.4(5)	O(18)	C(11)	N(10)	C(2)	-1.4(5)
C(6)	C(1)	C(2)	C(3)	-0.4(4)	C(20)	C(21)	C(22)	C(23)	-1.0(4)
C(6)	C(1)	C(2)	N(10)	-179.1(3)	C(21)	C(20)	C(25)	C(24)	0.4(4)
C(6)	C(1)	C(7)	O(8)	-7.7(4)	C(21)	C(20)	S(26)	O(27)	-61.5(2)
C(6)	C(1)	C(7)	O(9)	172.2(3)	C(21)	C(20)	S(26)	O(28)	178.5(2)
C(7)	C(1)	C(2)	C(3)	179.5(3)	C(21)	C(20)	S(26)	O(29)	55.6(2)
C(7)	C(1)	C(2)	N(10)	0.8(4)	C(21)	C(22)	C(23)	C(24)	2.0(4)
C(7)	C(1)	C(6)	C(5)	179.7(3)	C(21)	C(22)	C(23)	C(30)	-178.4(3)
C(11)	C(12)	C(13)	C(14)	179.1(3)	C(22)	C(23)	C(24)	C(25)	-1.8(4)
C(11)	C(12)	C(17)	C(16)	-179.1(3)	C(23)	C(24)	C(25)	C(20)	0.7(5)
C(11)	C(12)	C(17)	N(19)	1.8(4)	C(25)	C(20)	C(21)	C(22)	-0.2(4)
C(12)	C(11)	N(10)	C(2)	178.5(3)	C(25)	C(20)	S(26)	O(27)	116.4(2)
C(12)	C(13)	C(14)	C(15)	0.1(6)	C(25)	C(20)	S(26)	O(28)	-3.6(3)
C(13)	C(12)	C(17)	C(16)	1.1(4)	C(25)	C(20)	S(26)	O(29)	-126.5(2)
C(13)	C(12)	C(17)	N(19)	-177.9(3)	C(30)	C(23)	C(24)	C(25)	178.5(3)
C(13)	C(14)	C(15)	C(16)	1.1(5)	S(26)	C(20)	C(21)	C(22)	177.7(2)

C(14)	C(15)	C(16)	C(17)	-1.2(5)	S(26)	C(20)	C(25)	C(24)	-177.5(2)

Table 5S. Bond lengths for **14c.**

Atom	Atom	Length/Å	Atom	Atom	Length/Å
C(1)	C(2)	1.4246(14)	C(4)	C(5)	1.3971(16)
C(1)	C(6)	1.4040(15)	C(5)	C(6)	1.3782(15)
C(1)	C(7)	1.4689(14)	C(7)	O(8)	1.3231(13)
C(2)	C(3)	1.4160(15)	C(7)	O(9)	1.2379(12)
C(2)	N(10)	1.3571(14)	C(11)	N(10)	1.4446(14)
C(3)	C(4)	1.3751(15)			

Table 6S. Values of valence angles for **14c.**

Atom	Atom	Atom	Angle/°	Atom	Atom	Atom	Angle/°
C(2)	C(1)	C(7)	121.09(9)	C(3)	C(4)	C(5)	121.23(10)
C(6)	C(1)	C(2)	119.41(9)	C(6)	C(5)	C(4)	118.70(10)
C(6)	C(1)	C(7)	119.49(9)	C(5)	C(6)	C(1)	121.80(10)
C(3)	C(2)	C(1)	117.65(10)	O(8)	C(7)	C(1)	114.84(9)
N(10)	C(2)	C(1)	122.03(9)	O(9)	C(7)	C(1)	123.68(9)
N(10)	C(2)	C(3)	120.32(10)	O(9)	C(7)	O(8)	121.48(9)
C(4)	C(3)	C(2)	121.11(10)	C(2)	N(10)	C(11)	123.47(9)

Table 7S. Values of torsion angles for **14c.**

A	B	C	D	Angle/°	A	B	C	D	Angle/°
C(1)	C(2)	C(3)	C(4)	-2.52(15)	C(6)	C(1)	C(2)	C(3)	3.48(14)
C(1)	C(2)	N(10)	C(11)	175.42(9)	C(6)	C(1)	C(2)	N(10)	-176.84(9)
C(2)	C(1)	C(6)	C(5)	-1.71(15)	C(6)	C(1)	C(7)	O(8)	-0.83(13)
C(2)	C(1)	C(7)	O(8)	179.36(8)	C(6)	C(1)	C(7)	O(9)	179.75(9)
C(2)	C(1)	C(7)	O(9)	-0.07(15)	C(7)	C(1)	C(2)	C(3)	-176.71(9)
C(2)	C(3)	C(4)	C(5)	-0.32(16)	C(7)	C(1)	C(2)	N(10)	2.98(15)
C(3)	C(2)	N(10)	C(11)	-4.91(15)	C(7)	C(1)	C(6)	C(5)	178.47(9)
C(3)	C(4)	C(5)	C(6)	2.19(16)	N(10)	C(2)	C(3)	C(4)	177.80(9)
C(4)	C(5)	C(6)	C(1)	-1.15(16)					

Table 8S. Bond lengths for **10b.**

Atom	Atom	Length/Å	Atom	Atom	Length/Å
Br(17)	C(2)	1.8945(15)	C(8)	C(13)	1.396(2)
C(1)	C(2)	1.377(2)	C(9)	C(10)	1.384(2)
C(1)	C(16)	1.399(2)	C(10)	C(11)	1.389(3)
C(2)	C(3)	1.389(2)	C(11)	C(12)	1.386(2)
C(3)	C(4)	1.385(2)	C(12)	C(13)	1.393(2)
C(4)	C(5)	1.393(2)	C(13)	N(14)	1.4252(19)
C(5)	C(16)	1.393(2)	C(15)	C(16)	1.492(2)
C(5)	N(6)	1.424(2)	C(15)	N(14)	1.3424(19)
C(7)	C(8)	1.493(2)	C(15)	O(19)	1.2359(19)
C(7)	N(6)	1.347(2)	C(20)	C(21)	1.493(3)
C(7)	O(18)	1.2369(19)	C(20)	C(22)	1.508(3)
C(8)	C(9)	1.396(2)	C(20)	O(23)	1.205(2)

Table 9S. Values of valence angles for **10b.**

Atom	Atom	Atom	Angle/°	Atom	Atom	Atom	Angle/°
C(2)	C(1)	C(16)	119.50(14)	C(12)	C(11)	C(10)	119.92(16)
C(1)	C(2)	Br(17)	119.29(12)	C(11)	C(12)	C(13)	120.20(15)
C(1)	C(2)	C(3)	121.42(14)	C(8)	C(13)	N(14)	121.15(14)
C(3)	C(2)	Br(17)	119.16(12)	C(12)	C(13)	C(8)	120.03(14)

C(4)	C(3)	C(2)	118.82(15)	C(12)	C(13)	N(14)	118.44(14)
C(3)	C(4)	C(5)	120.89(15)	N(14)	C(15)	C(16)	120.37(13)
C(4)	C(5)	C(16)	119.56(14)	O(19)	C(15)	C(16)	118.15(13)
C(4)	C(5)	N(6)	118.09(14)	O(19)	C(15)	N(14)	121.47(14)
C(16)	C(5)	N(6)	121.91(14)	C(1)	C(16)	C(15)	116.33(13)
N(6)	C(7)	C(8)	120.01(13)	C(5)	C(16)	C(1)	119.80(14)
O(18)	C(7)	C(8)	119.20(13)	C(5)	C(16)	C(15)	122.95(14)
O(18)	C(7)	N(6)	120.77(14)	C(7)	N(6)	C(5)	128.09(13)
C(9)	C(8)	C(7)	117.44(14)	C(15)	N(14)	C(13)	126.35(13)
C(9)	C(8)	C(13)	119.29(14)	C(21)	C(20)	C(22)	116.42(18)
C(13)	C(8)	C(7)	122.89(14)	O(23)	C(20)	C(21)	121.97(19)
C(10)	C(9)	C(8)	120.38(16)	O(23)	C(20)	C(22)	121.6(2)
C(9)	C(10)	C(11)	120.18(16)				

Table 10S. Values of torsion angles for **10b.**

A	B	C	D	Angle/°	A	B	C	D	Angle/°
Br(17)	C(2)	C(3)	C(4)	-176.36(12)	C(11)	C(12)	C(13)	C(8)	0.5(2)
C(1)	C(2)	C(3)	C(4)	-0.6(3)	C(11)	C(12)	C(13)	N(14)	173.44(15)
C(2)	C(1)	C(16)	C(5)	1.2(2)	C(12)	C(13)	N(14)	C(15)	114.93(18)
C(2)	C(1)	C(16)	C(15)	-168.09(14)	C(13)	C(8)	C(9)	C(10)	0.4(2)
C(2)	C(3)	C(4)	C(5)	1.0(2)	C(16)	C(1)	C(2)	Br(17)	175.22(12)
C(3)	C(4)	C(5)	C(16)	-0.3(2)	C(16)	C(1)	C(2)	C(3)	-0.5(2)
C(3)	C(4)	C(5)	N(6)	172.23(15)	C(16)	C(5)	N(6)	C(7)	-67.0(2)
C(4)	C(5)	C(16)	C(1)	-0.8(2)	C(16)	C(15)	N(14)	C(13)	11.2(2)
C(4)	C(5)	C(16)	C(15)	167.78(14)	N(6)	C(5)	C(16)	C(1)	-173.09(14)
C(4)	C(5)	N(6)	C(7)	120.60(18)	N(6)	C(5)	C(16)	C(15)	-4.5(2)
C(7)	C(8)	C(9)	C(10)	-172.76(16)	N(6)	C(7)	C(8)	C(9)	-126.53(16)
C(7)	C(8)	C(13)	C(12)	171.93(14)	N(6)	C(7)	C(8)	C(13)	60.6(2)
C(7)	C(8)	C(13)	N(14)	-0.8(2)	N(14)	C(15)	C(16)	C(1)	-130.19(15)
C(8)	C(7)	N(6)	C(5)	7.8(2)	N(14)	C(15)	C(16)	C(5)	60.8(2)
C(8)	C(9)	C(10)	C(11)	0.4(3)	O(18)	C(7)	C(8)	C(9)	52.0(2)
C(8)	C(13)	N(14)	C(15)	-72.2(2)	O(18)	C(7)	C(8)	C(13)	-120.89(17)
C(9)	C(8)	C(13)	C(12)	-0.8(2)	O(18)	C(7)	N(6)	C(5)	-170.68(15)
C(9)	C(8)	C(13)	N(14)	-173.57(14)	O(19)	C(15)	C(16)	C(1)	50.7(2)
C(9)	C(10)	C(11)	C(12)	-0.7(3)	O(19)	C(15)	C(16)	C(5)	-118.29(17)
C(10)	C(11)	C(12)	C(13)	0.3(3)	O(19)	C(15)	N(14)	C(13)	-169.73(15)

Table 11S. Bond lengths for **10i.**

Atom	Atom	Length/Å	Atom	Atom	Length/Å
C(1)	C(2)	1.3869(18)	C(11)	C(12)	1.388(2)
C(1)	C(16)	1.3905(18)	C(12)	C(13)	1.3954(18)
C(2)	C(3)	1.3885(19)	C(13)	N(14)	1.4249(16)
C(3)	C(4)	1.3832(19)	C(15)	C(16)	1.4993(17)
C(4)	C(5)	1.3966(17)	C(15)	N(14)	1.3518(16)
C(5)	C(16)	1.3918(18)	C(15)	O(25)	1.2320(15)
C(5)	N(6)	1.4334(17)	C(17)	C(18)	1.5066(18)
C(7)	C(8)	1.5031(18)	C(17)	N(6)	1.4801(16)
C(7)	N(6)	1.3562(16)	C(18)	C(19)	1.3877(19)
C(7)	O(24)	1.2312(15)	C(18)	C(23)	1.3779(19)
C(8)	C(9)	1.3979(18)	C(19)	C(20)	1.381(2)
C(8)	C(13)	1.3948(19)	C(20)	C(21)	1.361(2)
C(9)	C(10)	1.387(2)	C(21)	C(22)	1.372(2)
C(10)	C(11)	1.385(2)	C(22)	C(23)	1.386(2)

Table 12S. Values of valence angles for **10i.**

Atom	Atom	Atom	Angle/°	Atom	Atom	Atom	Angle/°
C(2)	C(1)	C(16)	120.63(12)	N(14)	C(15)	C(16)	118.96(11)
C(1)	C(2)	C(3)	119.51(12)	O(25)	C(15)	C(16)	119.38(11)
C(4)	C(3)	C(2)	120.41(12)	O(25)	C(15)	N(14)	121.61(11)
C(3)	C(4)	C(5)	120.08(12)	C(1)	C(16)	C(5)	119.66(11)
C(4)	C(5)	N(6)	118.77(11)	C(1)	C(16)	C(15)	117.49(11)
C(16)	C(5)	C(4)	119.70(12)	C(5)	C(16)	C(15)	122.70(11)
C(16)	C(5)	N(6)	121.25(11)	N(6)	C(17)	C(18)	111.63(10)
N(6)	C(7)	C(8)	118.23(11)	C(19)	C(18)	C(17)	121.04(12)
O(24)	C(7)	C(8)	119.29(11)	C(23)	C(18)	C(17)	120.96(12)
O(24)	C(7)	N(6)	122.47(12)	C(23)	C(18)	C(19)	117.99(13)
C(9)	C(8)	C(7)	119.00(12)	C(20)	C(19)	C(18)	120.95(14)
C(13)	C(8)	C(7)	121.22(11)	C(21)	C(20)	C(19)	120.37(14)
C(13)	C(8)	C(9)	119.12(12)	C(20)	C(21)	C(22)	119.55(15)
C(10)	C(9)	C(8)	120.53(14)	C(21)	C(22)	C(23)	120.43(15)
C(11)	C(10)	C(9)	120.11(13)	C(18)	C(23)	C(22)	120.68(14)
C(10)	C(11)	C(12)	119.96(13)	C(5)	N(6)	C(17)	116.65(10)
C(11)	C(12)	C(13)	120.18(13)	C(7)	N(6)	C(5)	123.79(11)
C(8)	C(13)	C(12)	120.05(12)	C(7)	N(6)	C(17)	119.53(11)
C(8)	C(13)	N(14)	121.36(11)	C(15)	N(14)	C(13)	125.97(10)
C(12)	C(13)	N(14)	118.46(12)				

Table 13S. Values of torsion angles for **10i.**

A	B	C	D	Angle/°	A	B	C	D	Angle/°
C(1)	C(2)	C(3)	C(4)	-0.70(19)	C(16)	C(5)	N(6)	C(17)	105.64(13)
C(2)	C(1)	C(16)	C(5)	-0.33(19)	C(16)	C(15)	N(14)	C(13)	8.4(2)
C(2)	C(1)	C(16)	C(15)	-176.09(11)	C(17)	C(18)	C(19)	C(20)	179.33(15)
C(2)	C(3)	C(4)	C(5)	-0.14(19)	C(17)	C(18)	C(23)	C(22)	-179.95(15)
C(3)	C(4)	C(5)	C(16)	0.74(18)	C(18)	C(17)	N(6)	C(5)	-78.62(13)
C(3)	C(4)	C(5)	N(6)	174.81(11)	C(18)	C(17)	N(6)	C(7)	99.39(13)
C(4)	C(5)	C(16)	C(1)	-0.51(18)	C(18)	C(19)	C(20)	C(21)	0.6(3)
C(4)	C(5)	C(16)	C(15)	175.02(11)	C(19)	C(18)	C(23)	C(22)	-1.4(2)
C(4)	C(5)	N(6)	C(7)	113.76(13)	C(19)	C(20)	C(21)	C(22)	-1.3(3)
C(4)	C(5)	N(6)	C(17)	-68.33(14)	C(20)	C(21)	C(22)	C(23)	0.7(3)
C(7)	C(8)	C(9)	C(10)	-170.84(12)	C(21)	C(22)	C(23)	C(18)	0.7(3)
C(7)	C(8)	C(13)	C(12)	168.64(11)	C(23)	C(18)	C(19)	C(20)	0.8(2)
C(7)	C(8)	C(13)	N(14)	-7.13(18)	N(6)	C(5)	C(16)	C(1)	-174.43(11)
C(8)	C(7)	N(6)	C(5)	5.75(17)	N(6)	C(5)	C(16)	C(15)	1.10(18)
C(8)	C(7)	N(6)	C(17)	-172.10(11)	N(6)	C(7)	C(8)	C(9)	-121.09(13)
C(8)	C(9)	C(10)	C(11)	1.9(2)	N(6)	C(7)	C(8)	C(13)	68.30(16)
C(8)	C(13)	N(14)	C(15)	-68.39(18)	N(6)	C(17)	C(18)	C(19)	-68.32(17)
C(9)	C(8)	C(13)	C(12)	-1.96(18)	N(6)	C(17)	C(18)	C(23)	110.17(15)
C(9)	C(8)	C(13)	N(14)	-177.74(11)	N(14)	C(15)	C(16)	C(1)	-123.00(13)
C(9)	C(10)	C(11)	C(12)	-1.7(2)	N(14)	C(15)	C(16)	C(5)	61.38(17)
C(10)	C(11)	C(12)	C(13)	-0.2(2)	O(24)	C(7)	C(8)	C(9)	59.53(16)
C(11)	C(12)	C(13)	C(8)	2.11(19)	O(24)	C(7)	C(8)	C(13)	-111.08(14)
C(11)	C(12)	C(13)	N(14)	178.01(12)	O(24)	C(7)	N(6)	C(5)	-174.89(11)
C(12)	C(13)	N(14)	C(15)	115.77(15)	O(24)	C(7)	N(6)	C(17)	7.26(18)
C(13)	C(8)	C(9)	C(10)	-0.02(19)	O(25)	C(15)	C(16)	C(1)	54.37(17)
C(16)	C(1)	C(2)	C(3)	0.94(19)	O(25)	C(15)	C(16)	C(5)	-121.25(14)
C(16)	C(5)	N(6)	C(7)	-72.27(16)	O(25)	C(15)	N(14)	C(13)	-168.90(13)

Table 14S. Bond lengths for **10g.**

Atom	Atom	Length/Å	Atom	Atom	Length/Å
C(1)	C(2)	1.386(2)	C(10)	C(11)	1.415(2)
C(1)	C(16)	1.397(2)	C(10)	O(19)	1.3693(16)
C(2)	C(3)	1.386(2)	C(11)	C(12)	1.3856(19)
C(3)	C(4)	1.388(2)	C(11)	O(21)	1.3559(17)
C(4)	C(5)	1.396(2)	C(12)	C(13)	1.400(2)
C(5)	C(16)	1.387(2)	C(13)	N(14)	1.4307(18)
C(5)	N(6)	1.4373(17)	C(15)	C(16)	1.5015(19)
C(7)	C(8)	1.4927(19)	C(15)	N(14)	1.3520(18)
C(7)	N(6)	1.3527(18)	C(15)	O(23)	1.2289(17)
C(7)	O(18)	1.2361(17)	C(17)	N(6)	1.4671(18)
C(8)	C(9)	1.4055(19)	C(20)	O(19)	1.4289(17)
C(8)	C(13)	1.388(2)	C(22)	O(21)	1.4303(18)
C(9)	C(10)	1.375(2)			

Table 15S. Values of valence angles for **10g.**

Atom	Atom	Atom	Angle/°	Atom	Atom	Atom	Angle/°
C(2)	C(1)	C(16)	120.24(13)	O(21)	C(11)	C(10)	114.90(12)
C(3)	C(2)	C(1)	119.97(13)	O(21)	C(11)	C(12)	125.60(13)
C(2)	C(3)	C(4)	120.34(13)	C(11)	C(12)	C(13)	120.35(13)
C(3)	C(4)	C(5)	119.66(13)	C(8)	C(13)	C(12)	120.20(13)
C(4)	C(5)	N(6)	118.68(12)	C(8)	C(13)	N(14)	121.64(13)
C(16)	C(5)	C(4)	120.26(13)	C(12)	C(13)	N(14)	117.99(12)
C(16)	C(5)	N(6)	120.86(12)	N(14)	C(15)	C(16)	117.11(12)
N(6)	C(7)	C(8)	118.57(12)	O(23)	C(15)	C(16)	120.66(12)
O(18)	C(7)	C(8)	120.26(12)	O(23)	C(15)	N(14)	122.22(12)
O(18)	C(7)	N(6)	121.17(13)	C(1)	C(16)	C(15)	118.54(12)
C(9)	C(8)	C(7)	117.16(12)	C(5)	C(16)	C(1)	119.52(13)
C(13)	C(8)	C(7)	123.02(12)	C(5)	C(16)	C(15)	121.75(12)
C(13)	C(8)	C(9)	119.30(13)	C(5)	N(6)	C(17)	117.36(11)
C(10)	C(9)	C(8)	120.85(13)	C(7)	N(6)	C(5)	123.48(11)
C(9)	C(10)	C(11)	119.77(13)	C(7)	N(6)	C(17)	118.81(12)
O(19)	C(10)	C(9)	125.23(13)	C(15)	N(14)	C(13)	126.41(12)
O(19)	C(10)	C(11)	114.98(12)	C(10)	O(19)	C(20)	117.18(11)
C(12)	C(11)	C(10)	119.51(13)	C(11)	O(21)	C(22)	117.63(11)

Table 16S. Values of torsion angles for **10g.**

A	B	C	D	Angle/°	A	B	C	D	Angle/°
C(1)	C(2)	C(3)	C(4)	0.2(2)	C(11)	C(12)	C(13)	C(8)	-0.2(2)
C(2)	C(1)	C(16)	C(5)	0.3(2)	C(11)	C(12)	C(13)	N(14)	-175.54(12)
C(2)	C(1)	C(16)	C(15)	175.29(12)	C(12)	C(11)	O(21)	C(22)	-2.7(2)
C(2)	C(3)	C(4)	C(5)	0.6(2)	C(12)	C(13)	N(14)	C(15)	-115.85(16)
C(3)	C(4)	C(5)	C(16)	-1.0(2)	C(13)	C(8)	C(9)	C(10)	1.2(2)
C(3)	C(4)	C(5)	N(6)	-175.87(12)	C(16)	C(1)	C(2)	C(3)	-0.6(2)
C(4)	C(5)	C(16)	C(1)	0.55(19)	C(16)	C(5)	N(6)	C(7)	75.63(17)
C(4)	C(5)	C(16)	C(15)	-174.31(12)	C(16)	C(5)	N(6)	C(17)	-111.20(15)
C(4)	C(5)	N(6)	C(7)	-109.53(15)	C(16)	C(15)	N(14)	C(13)	-4.3(2)
C(4)	C(5)	N(6)	C(17)	63.64(17)	N(6)	C(5)	C(16)	C(1)	175.30(12)
C(7)	C(8)	C(9)	C(10)	173.19(12)	N(6)	C(5)	C(16)	C(15)	0.45(19)
C(7)	C(8)	C(13)	C(12)	-172.62(12)	N(6)	C(7)	C(8)	C(9)	126.92(13)
C(7)	C(8)	C(13)	N(14)	2.6(2)	N(6)	C(7)	C(8)	C(13)	-61.41(18)
C(8)	C(7)	N(6)	C(5)	-11.37(19)	N(14)	C(15)	C(16)	C(1)	119.93(14)

C(8)	C(7)	N(6)	C(17)	175.56(12)	N(14)	C(15)	C(16)	C(5)	-65.16(17)
C(8)	C(9)	C(10)	C(11)	0.0(2)	O(18)	C(7)	C(8)	C(9)	-52.67(17)
C(8)	C(9)	C(10)	O(19)	-178.59(12)	O(18)	C(7)	C(8)	C(13)	119.00(15)
C(8)	C(13)	N(14)	C(15)	68.86(19)	O(18)	C(7)	N(6)	C(5)	168.22(12)
C(9)	C(8)	C(13)	C(12)	-1.12(19)	O(18)	C(7)	N(6)	C(17)	-4.85(19)
C(9)	C(8)	C(13)	N(14)	174.07(12)	O(19)	C(10)	C(11)	C(12)	177.43(11)
C(9)	C(10)	C(11)	C(12)	-1.3(2)	O(19)	C(10)	C(11)	O(21)	-2.27(17)
C(9)	C(10)	C(11)	O(21)	178.98(12)	O(21)	C(11)	C(12)	C(13)	-178.94(12)
C(9)	C(10)	O(19)	C(20)	13.16(19)	O(23)	C(15)	C(16)	C(1)	-59.07(18)
C(10)	C(11)	C(12)	C(13)	1.4(2)	O(23)	C(15)	C(16)	C(5)	115.84(15)
C(10)	C(11)	O(21)	C(22)	176.94(13)	O(23)	C(15)	N(14)	C(13)	174.70(13)
C(11)	C(10)	O(19)	C(20)	-165.52(12)					

Table 17S. Bond lengths for **10l.**

Atom	Atom	Length/Å	Atom	Atom	Length/Å
C(1)	C(2)	1.391(4)	C(8)	C(9)	1.385(4)
C(1)	C(16)	1.394(4)	C(8)	C(13)	1.404(4)
C(2)	C(3)	1.392(4)	C(9)	C(10)	1.391(4)
C(2)	Cl(17)	1.743(3)	C(10)	C(11)	1.384(4)
C(3)	C(4)	1.377(4)	C(11)	N(12)	1.332(4)
C(4)	C(5)	1.400(4)	C(13)	N(12)	1.350(3)
C(5)	C(16)	1.398(4)	C(13)	N(14)	1.411(4)
C(5)	N(6)	1.428(4)	C(15)	C(16)	1.518(4)
C(7)	C(8)	1.501(3)	C(15)	N(14)	1.362(4)
C(7)	N(6)	1.342(4)	C(15)	O(19)	1.219(4)
C(7)	O(18)	1.227(4)			

Table 18S. Values of valence angles for **10l.**

Atom	Atom	Atom	Angle/°	Atom	Atom	Atom	Angle/°
C(2)	C(1)	C(16)	119.2(2)	C(8)	C(9)	C(10)	119.9(2)
C(1)	C(2)	C(3)	121.3(3)	C(11)	C(10)	C(9)	117.5(3)
C(1)	C(2)	Cl(17)	119.5(2)	N(12)	C(11)	C(10)	124.2(2)
C(3)	C(2)	Cl(17)	119.2(2)	C(8)	C(13)	N(14)	123.1(2)
C(4)	C(3)	C(2)	119.1(2)	N(12)	C(13)	C(8)	121.9(3)
C(3)	C(4)	C(5)	120.8(2)	N(12)	C(13)	N(14)	114.6(2)
C(4)	C(5)	N(6)	116.2(2)	N(14)	C(15)	C(16)	120.1(2)
C(16)	C(5)	C(4)	119.7(3)	O(19)	C(15)	C(16)	118.9(3)
C(16)	C(5)	N(6)	123.7(3)	O(19)	C(15)	N(14)	120.9(2)
N(6)	C(7)	C(8)	119.7(2)	C(1)	C(16)	C(5)	119.9(2)
O(18)	C(7)	C(8)	118.5(3)	C(1)	C(16)	C(15)	115.7(2)
O(18)	C(7)	N(6)	121.7(2)	C(5)	C(16)	C(15)	124.1(3)
C(9)	C(8)	C(7)	116.6(2)	C(7)	N(6)	C(5)	128.0(2)
C(9)	C(8)	C(13)	118.4(2)	C(11)	N(12)	C(13)	118.1(2)
C(13)	C(8)	C(7)	124.6(3)	C(15)	N(14)	C(13)	127.9(2)

Table 19S. Values of torsion angles for **10l.**

A	B	C	D	Angle/°	A	B	C	D	Angle/°
C(1)	C(2)	C(3)	C(4)	-0.9(4)	C(13)	C(8)	C(9)	C(10)	-0.6(4)
C(2)	C(1)	C(16)	C(5)	-0.1(4)	C(16)	C(1)	C(2)	C(3)	0.9(4)
C(2)	C(1)	C(16)	C(15)	-173.8(2)	C(16)	C(1)	C(2)	Cl(17)	179.4(2)
C(2)	C(3)	C(4)	C(5)	0.1(4)	C(16)	C(5)	N(6)	C(7)	-72.2(4)
C(3)	C(4)	C(5)	C(16)	0.7(4)	C(16)	C(15)	N(14)	C(13)	15.3(4)
C(3)	C(4)	C(5)	N(6)	173.4(3)	Cl(17)	C(2)	C(3)	C(4)	-179.4(2)

C(4)	C(5)	C(16)	C(1)	-0.7(4)	N(6)	C(5)	C(16)	C(1)	-172.8(3)
C(4)	C(5)	C(16)	C(15)	172.5(3)	N(6)	C(5)	C(16)	C(15)	0.4(4)
C(4)	C(5)	N(6)	C(7)	115.5(3)	N(6)	C(7)	C(8)	C(9)	-131.4(3)
C(7)	C(8)	C(9)	C(10)	-173.9(3)	N(6)	C(7)	C(8)	C(13)	55.7(4)
C(7)	C(8)	C(13)	N(12)	170.3(3)	N(12)	C(13)	N(14)	C(15)	115.7(3)
C(7)	C(8)	C(13)	N(14)	-2.0(4)	N(14)	C(13)	N(12)	C(11)	176.1(2)
C(8)	C(7)	N(6)	C(5)	14.1(4)	N(14)	C(15)	C(16)	C(1)	-133.6(3)
C(8)	C(9)	C(10)	C(11)	2.5(4)	N(14)	C(15)	C(16)	C(5)	53.0(4)
C(8)	C(13)	N(12)	C(11)	3.2(4)	O(18)	C(7)	C(8)	C(9)	45.6(4)
C(8)	C(13)	N(14)	C(15)	-71.4(4)	O(18)	C(7)	C(8)	C(13)	-127.3(3)
C(9)	C(8)	C(13)	N(12)	-2.4(4)	O(18)	C(7)	N(6)	C(5)	-162.8(3)
C(9)	C(8)	C(13)	N(14)	-174.7(3)	O(19)	C(15)	C(16)	C(1)	43.6(3)
C(9)	C(10)	C(11)	N(12)	-1.7(4)	O(19)	C(15)	C(16)	C(5)	-129.8(3)
C(10)	C(11)	N(12)	C(13)	-1.1(4)	O(19)	C(15)	N(14)	C(13)	-161.8(2)

Table 20S. Bond lengths for **10j.**

Atom	Atom	Length/Å	Atom	Atom	Length/Å
Br(24)	C(21)	1.901(3)	C(3)	C(4)	1.382(4)
C(9)	C(10)	1.382(4)	C(4)	C(5)	1.391(4)
C(9)	C(8)	1.403(4)	C(5)	N(6)	1.440(4)
C(10)	C(11)	1.387(4)	C(7)	C(8)	1.494(4)
C(11)	C(12)	1.390(4)	C(7)	N(6)	1.353(4)
C(12)	C(13)	1.395(4)	C(7)	O(25)	1.234(3)
C(13)	C(8)	1.390(4)	C(17)	C(18)	1.520(4)
C(13)	N(14)	1.431(4)	C(17)	N(6)	1.478(4)
C(15)	C(16)	1.503(4)	C(18)	C(19)	1.382(4)
C(15)	N(14)	1.350(4)	C(18)	C(23)	1.398(4)
C(15)	O(26)	1.228(3)	C(19)	C(20)	1.390(4)
C(16)	C(1)	1.399(4)	C(20)	C(21)	1.388(5)
C(16)	C(5)	1.399(4)	C(21)	C(22)	1.385(5)
C(1)	C(2)	1.387(4)	C(22)	C(23)	1.384(4)
C(2)	C(3)	1.391(4)			

Table 21S. Values of valence angles for **10j.**

Atom	Atom	Atom	Angle/°	Atom	Atom	Atom	Angle/°
C(10)	C(9)	C(8)	120.7(3)	O(25)	C(7)	C(8)	119.0(3)
C(9)	C(10)	C(11)	119.7(3)	O(25)	C(7)	N(6)	121.6(3)
C(10)	C(11)	C(12)	120.3(3)	C(9)	C(8)	C(7)	117.3(3)
C(11)	C(12)	C(13)	120.1(3)	C(13)	C(8)	C(9)	119.4(3)
C(12)	C(13)	N(14)	118.4(3)	C(13)	C(8)	C(7)	122.6(2)
C(8)	C(13)	C(12)	119.8(3)	N(6)	C(17)	C(18)	111.7(2)
C(8)	C(13)	N(14)	121.6(3)	C(19)	C(18)	C(17)	120.1(3)
N(14)	C(15)	C(16)	117.9(2)	C(19)	C(18)	C(23)	119.3(3)
O(26)	C(15)	C(16)	120.4(3)	C(23)	C(18)	C(17)	120.5(3)
O(26)	C(15)	N(14)	121.7(3)	C(18)	C(19)	C(20)	120.8(3)
C(1)	C(16)	C(15)	117.5(3)	C(21)	C(20)	C(19)	119.0(3)
C(5)	C(16)	C(15)	123.6(3)	C(20)	C(21)	Br(24)	119.3(2)
C(5)	C(16)	C(1)	118.7(3)	C(22)	C(21)	Br(24)	119.6(2)
C(2)	C(1)	C(16)	121.0(3)	C(22)	C(21)	C(20)	121.1(3)
C(1)	C(2)	C(3)	119.6(3)	C(23)	C(22)	C(21)	119.3(3)
C(4)	C(3)	C(2)	120.1(3)	C(22)	C(23)	C(18)	120.4(3)
C(3)	C(4)	C(5)	120.6(3)	C(15)	N(14)	C(13)	127.4(2)
C(16)	C(5)	N(6)	121.5(3)	C(5)	N(6)	C(17)	116.8(2)

C(4)	C(5)	C(16)	120.0(3)	C(7)	N(6)	C(5)	123.9(2)
C(4)	C(5)	N(6)	118.2(3)	C(7)	N(6)	C(17)	117.6(2)
N(6)	C(7)	C(8)	119.4(2)				

Table 22S. Values of torsion angles for **10j.**

A	B	C	D	Angle/°	A	B	C	D	Angle/°
Br(24)	C(21)	C(22)	C(23)	-179.8(2)	C(8)	C(7)	N(6)	C(5)	-13.0(4)
C(9)	C(10)	C(11)	C(12)	0.1(5)	C(8)	C(7)	N(6)	C(17)	-177.4(2)
C(10)	C(9)	C(8)	C(13)	1.2(4)	C(17)	C(18)	C(19)	C(20)	-179.1(3)
C(10)	C(9)	C(8)	C(7)	172.2(3)	C(17)	C(18)	C(23)	C(22)	-179.2(3)
C(10)	C(11)	C(12)	C(13)	1.6(4)	C(18)	C(17)	N(6)	C(5)	57.2(3)
C(11)	C(12)	C(13)	C(8)	-1.9(4)	C(18)	C(17)	N(6)	C(7)	-137.3(3)
C(11)	C(12)	C(13)	N(14)	-176.6(3)	C(18)	C(19)	C(20)	C(21)	-1.6(5)
C(12)	C(13)	C(8)	C(9)	0.6(4)	C(19)	C(18)	C(23)	C(22)	1.2(5)
C(12)	C(13)	C(8)	C(7)	-170.0(3)	C(19)	C(20)	C(21)	Br(24)	-178.6(2)
C(12)	C(13)	N(14)	C(15)	-112.6(3)	C(19)	C(20)	C(21)	C(22)	1.1(5)
C(15)	C(16)	C(1)	C(2)	175.3(3)	C(20)	C(21)	C(22)	C(23)	0.5(5)
C(15)	C(16)	C(5)	C(4)	-175.0(3)	C(21)	C(22)	C(23)	C(18)	-1.7(5)
C(15)	C(16)	C(5)	N(6)	-1.6(4)	C(23)	C(18)	C(19)	C(20)	0.4(5)
C(16)	C(15)	N(14)	C(13)	-14.3(4)	N(14)	C(13)	C(8)	C(9)	175.1(3)
C(16)	C(1)	C(2)	C(3)	-0.1(4)	N(14)	C(13)	C(8)	C(7)	4.5(4)
C(16)	C(5)	N(6)	C(7)	76.1(4)	N(14)	C(15)	C(16)	C(1)	129.0(3)
C(16)	C(5)	N(6)	C(17)	-119.4(3)	N(14)	C(15)	C(16)	C(5)	-56.4(4)
C(1)	C(16)	C(5)	C(4)	-0.4(4)	N(6)	C(7)	C(8)	C(9)	128.7(3)
C(1)	C(16)	C(5)	N(6)	173.0(2)	N(6)	C(7)	C(8)	C(13)	-60.6(4)
C(1)	C(2)	C(3)	C(4)	-0.2(4)	N(6)	C(17)	C(18)	C(19)	-134.3(3)
C(2)	C(3)	C(4)	C(5)	0.2(4)	N(6)	C(17)	C(18)	C(23)	46.1(4)
C(3)	C(4)	C(5)	C(16)	0.1(4)	O(26)	C(15)	C(16)	C(1)	-49.8(4)
C(3)	C(4)	C(5)	N(6)	-173.5(3)	O(26)	C(15)	C(16)	C(5)	124.9(3)
C(4)	C(5)	N(6)	C(7)	-110.3(3)	O(26)	C(15)	N(14)	C(13)	164.4(3)
C(4)	C(5)	N(6)	C(17)	54.2(3)	O(25)	C(7)	C(8)	C(9)	-51.8(4)
C(5)	C(16)	C(1)	C(2)	0.4(4)	O(25)	C(7)	C(8)	C(13)	118.9(3)
C(8)	C(9)	C(10)	C(11)	-1.5(4)	O(25)	C(7)	N(6)	C(5)	167.5(3)
C(8)	C(13)	N(14)	C(15)	72.8(4)	O(25)	C(7)	N(6)	C(17)	3.1(4)

Table 23S. Bond lengths for **10m.**

Atom	Atom	Length/Å	Atom	Atom	Length/Å
C(10)	C(11)	1.387(2)	C(1)	C(2)	1.382(2)
C(10)	N(9)	1.331(2)	C(2)	C(3)	1.391(2)
C(11)	N(12)	1.331(2)	C(2)	Cl(17)	1.7428(16)
C(13)	C(8)	1.397(2)	C(3)	C(4)	1.387(2)
C(13)	N(12)	1.335(2)	C(4)	C(5)	1.391(2)
C(13)	N(14)	1.408(2)	C(5)	N(6)	1.426(2)
C(15)	C(16)	1.507(2)	C(7)	C(8)	1.508(2)
C(15)	N(14)	1.357(2)	C(7)	N(6)	1.349(2)
C(15)	O(19)	1.2290(19)	C(7)	O(18)	1.227(2)
C(16)	C(1)	1.393(2)	C(8)	N(9)	1.339(2)
C(16)	C(5)	1.401(2)			

Table 24S. Values of valence angles for **10m.**

Atom	Atom	Atom	Angle/°	Atom	Atom	Atom	Angle/°
N(9)	C(10)	C(11)	121.73(14)	C(4)	C(3)	C(2)	118.68(15)
N(12)	C(11)	C(10)	122.34(14)	C(3)	C(4)	C(5)	120.61(14)

C(8)	C(13)	N(14)	122.17(14)	C(16)	C(5)	N(6)	120.93(14)
N(12)	C(13)	C(8)	121.54(14)	C(4)	C(5)	C(16)	119.99(14)
N(12)	C(13)	N(14)	116.09(13)	C(4)	C(5)	N(6)	119.03(13)
N(14)	C(15)	C(16)	118.96(13)	N(6)	C(7)	C(8)	118.77(13)
O(19)	C(15)	C(16)	119.75(13)	O(18)	C(7)	C(8)	117.79(13)
O(19)	C(15)	N(14)	121.25(14)	O(18)	C(7)	N(6)	123.44(14)
C(1)	C(16)	C(15)	116.43(13)	C(13)	C(8)	C(7)	123.25(14)
C(1)	C(16)	C(5)	119.53(14)	N(9)	C(8)	C(13)	121.69(14)
C(5)	C(16)	C(15)	123.88(14)	N(9)	C(8)	C(7)	114.37(13)
C(2)	C(1)	C(16)	119.46(14)	C(10)	N(9)	C(8)	116.24(13)
C(1)	C(2)	C(3)	121.69(15)	C(11)	N(12)	C(13)	116.12(13)
C(1)	C(2)	Cl(17)	119.10(12)	C(15)	N(14)	C(13)	125.90(13)
C(3)	C(2)	Cl(17)	119.19(12)	C(7)	N(6)	C(5)	123.60(13)

Table 25S. Values of torsion angles for **10m.**

A	B	C	D	Angle/°	A	B	C	D	Angle/°
C(10)	C(11)	N(12)	C(13)	-0.9(2)	C(8)	C(13)	N(14)	C(15)	63.6(2)
C(11)	C(10)	N(9)	C(8)	-2.6(2)	C(8)	C(7)	N(6)	C(5)	-10.8(2)
C(13)	C(8)	N(9)	C(10)	-2.6(2)	Cl(17)	C(2)	C(3)	C(4)	179.81(11)
C(15)	C(16)	C(1)	C(2)	174.08(13)	N(9)	C(10)	C(11)	N(12)	4.6(2)
C(15)	C(16)	C(5)	C(4)	-173.10(14)	N(12)	C(13)	C(8)	C(7)	-163.50(14)
C(15)	C(16)	C(5)	N(6)	4.2(2)	N(12)	C(13)	C(8)	N(9)	6.4(2)
C(16)	C(15)	N(14)	C(13)	-7.8(2)	N(12)	C(13)	N(14)	C(15)	-121.46(16)
C(16)	C(1)	C(2)	C(3)	-0.3(2)	N(14)	C(13)	C(8)	C(7)	11.2(2)
C(16)	C(1)	C(2)	Cl(17)	-178.74(11)	N(14)	C(13)	C(8)	N(9)	-178.94(13)
C(16)	C(5)	N(6)	C(7)	70.6(2)	N(14)	C(13)	N(12)	C(11)	-179.28(13)
C(1)	C(16)	C(5)	C(4)	2.1(2)	N(14)	C(15)	C(16)	C(1)	120.20(15)
C(1)	C(16)	C(5)	N(6)	179.32(13)	N(14)	C(15)	C(16)	C(5)	-64.5(2)
C(1)	C(2)	C(3)	C(4)	1.3(2)	N(6)	C(7)	C(8)	C(13)	-67.3(2)
C(2)	C(3)	C(4)	C(5)	-0.7(2)	N(6)	C(7)	C(8)	N(9)	122.16(15)
C(3)	C(4)	C(5)	C(16)	-1.0(2)	O(19)	C(15)	C(16)	C(1)	-57.50(19)
C(3)	C(4)	C(5)	N(6)	-178.30(13)	O(19)	C(15)	C(16)	C(5)	117.81(16)
C(4)	C(5)	N(6)	C(7)	-112.14(17)	O(19)	C(15)	N(14)	C(13)	169.87(14)
C(5)	C(16)	C(1)	C(2)	-1.4(2)	O(18)	C(7)	C(8)	C(13)	113.00(17)
C(7)	C(8)	N(9)	C(10)	168.08(14)	O(18)	C(7)	C(8)	N(9)	-57.5(2)
C(8)	C(13)	N(12)	C(11)	-4.3(2)	O(18)	C(7)	N(6)	C(5)	168.89(15)

Table 26S. Bond lengths for **10h**.

Atom	Atom	Length/Å	Atom	Atom	Length/Å
C(1)	C(2)	1.386(6)	C(10)	C(11)	1.426(6)
C(1)	C(20)	1.401(6)	C(10)	C(15)	1.423(6)
C(2)	C(3)	1.386(6)	C(11)	C(12)	1.358(6)
C(2)	Cl(21)	1.740(4)	C(12)	C(13)	1.401(6)
C(3)	C(4)	1.388(6)	C(13)	C(14)	1.371(6)
C(4)	C(5)	1.393(6)	C(14)	C(15)	1.417(6)
C(5)	C(20)	1.386(6)	C(15)	C(16)	1.410(6)
C(5)	N(6)	1.435(5)	C(16)	C(17)	1.364(6)
C(7)	C(8)	1.501(6)	C(17)	N(18)	1.433(5)
C(7)	N(6)	1.362(5)	C(19)	C(20)	1.502(5)
C(7)	O(23)	1.232(5)	C(19)	N(18)	1.352(5)
C(8)	C(9)	1.378(6)	C(19)	O(24)	1.223(5)
C(8)	C(17)	1.431(6)	C(22)	N(6)	1.463(5)

C(9)	C(10)	1.418(5)

Table 27S. Values of valence angles for **10h**.

Atom	Atom	Atom	Angle/°	Atom	Atom	Atom	Angle/°
C(2)	C(1)	C(20)	119.1(4)	C(11)	C(12)	C(13)	121.1(4)
C(1)	C(2)	C(3)	121.5(4)	C(14)	C(13)	C(12)	120.3(4)
C(1)	C(2)	Cl(21)	118.7(3)	C(13)	C(14)	C(15)	120.7(4)
C(3)	C(2)	Cl(21)	119.8(3)	C(14)	C(15)	C(10)	118.7(4)
C(2)	C(3)	C(4)	118.9(4)	C(16)	C(15)	C(10)	118.4(4)
C(3)	C(4)	C(5)	120.5(4)	C(16)	C(15)	C(14)	123.0(4)
C(4)	C(5)	N(6)	118.6(4)	C(17)	C(16)	C(15)	121.9(4)
C(20)	C(5)	C(4)	120.2(4)	C(8)	C(17)	N(18)	121.7(4)
C(20)	C(5)	N(6)	121.2(4)	C(16)	C(17)	C(8)	120.1(4)
N(6)	C(7)	C(8)	118.1(4)	C(16)	C(17)	N(18)	118.0(4)
O(23)	C(7)	C(8)	120.0(4)	N(18)	C(19)	C(20)	117.6(4)
O(23)	C(7)	N(6)	121.9(4)	O(24)	C(19)	C(20)	119.9(4)
C(9)	C(8)	C(7)	117.6(4)	O(24)	C(19)	N(18)	122.6(4)
C(9)	C(8)	C(17)	119.1(4)	C(1)	C(20)	C(19)	118.7(4)
C(17)	C(8)	C(7)	122.7(4)	C(5)	C(20)	C(1)	119.8(4)
C(8)	C(9)	C(10)	121.2(4)	C(5)	C(20)	C(19)	121.3(4)
C(9)	C(10)	C(11)	121.8(4)	C(5)	N(6)	C(22)	117.0(3)
C(9)	C(10)	C(15)	119.2(4)	C(7)	N(6)	C(5)	121.5(3)
C(15)	C(10)	C(11)	118.9(4)	C(7)	N(6)	C(22)	120.6(3)
C(12)	C(11)	C(10)	120.3(4)	C(19)	N(18)	C(17)	125.5(3)

Table 28S. Values of torsion angles for **10h**.

A	B	C	D	Angle/°	A	B	C	D	Angle/°
C(1)	C(2)	C(3)	C(4)	-0.8(7)	C(12)	C(13)	C(14)	C(15)	-0.3(6)
C(2)	C(1)	C(20)	C(5)	-0.3(6)	C(13)	C(14)	C(15)	C(10)	0.2(6)
C(2)	C(1)	C(20)	C(19)	-176.4(4)	C(13)	C(14)	C(15)	C(16)	-179.9(4)
C(2)	C(3)	C(4)	C(5)	-0.2(7)	C(14)	C(15)	C(16)	C(17)	-179.5(4)
C(3)	C(4)	C(5)	C(20)	1.0(7)	C(15)	C(10)	C(11)	C(12)	0.4(6)
C(3)	C(4)	C(5)	N(6)	177.2(4)	C(15)	C(16)	C(17)	C(8)	0.9(6)
C(4)	C(5)	C(20)	C(1)	-0.7(6)	C(15)	C(16)	C(17)	N(18)	175.9(4)
C(4)	C(5)	C(20)	C(19)	175.2(4)	C(16)	C(17)	N(18)	C(19)	114.0(4)
C(4)	C(5)	N(6)	C(7)	102.0(5)	C(17)	C(8)	C(9)	C(10)	0.4(5)
C(4)	C(5)	N(6)	C(22)	-66.9(5)	C(20)	C(1)	C(2)	C(3)	1.1(6)
C(7)	C(8)	C(9)	C(10)	-171.3(3)	C(20)	C(1)	C(2)	Cl(21)	-178.4(3)
C(7)	C(8)	C(17)	C(16)	170.0(4)	C(20)	C(5)	N(6)	C(7)	-81.9(5)
C(7)	C(8)	C(17)	N(18)	-4.8(6)	C(20)	C(5)	N(6)	C(22)	109.2(4)
C(8)	C(7)	N(6)	C(5)	20.3(6)	C(20)	C(19)	N(18)	C(17)	10.5(6)
C(8)	C(7)	N(6)	C(22)	-171.2(3)	Cl(21)	C(2)	C(3)	C(4)	178.7(3)
C(8)	C(9)	C(10)	C(11)	179.7(4)	N(6)	C(5)	C(20)	C(1)	-176.8(4)
C(8)	C(9)	C(10)	C(15)	0.8(5)	N(6)	C(5)	C(20)	C(19)	-0.9(6)
C(8)	C(17)	N(18)	C(19)	-71.1(5)	N(6)	C(7)	C(8)	C(9)	-131.8(4)
C(9)	C(8)	C(17)	C(16)	-1.2(6)	N(6)	C(7)	C(8)	C(17)	56.8(5)
C(9)	C(8)	C(17)	N(18)	-176.1(3)	N(18)	C(19)	C(20)	C(1)	-121.5(4)
C(9)	C(10)	C(11)	C(12)	-178.5(4)	N(18)	C(19)	C(20)	C(5)	62.4(5)
C(9)	C(10)	C(15)	C(14)	178.7(3)	O(23)	C(7)	C(8)	C(9)	49.5(5)
C(9)	C(10)	C(15)	C(16)	-1.2(5)	O(23)	C(7)	C(8)	C(17)	-121.9(4)
C(10)	C(11)	C(12)	C(13)	-0.5(6)	O(23)	C(7)	N(6)	C(5)	-161.0(4)
C(10)	C(15)	C(16)	C(17)	0.4(6)	O(23)	C(7)	N(6)	C(22)	7.6(6)
C(11)	C(10)	C(15)	C(14)	-0.2(5)	O(24)	C(19)	C(20)	C(1)	57.5(6)

C(11)	C(10)	C(15)	C(16)	179.9(4)	O(24)	C(19)	C(20)	C(5)	-118.5(5)
C(11)	C(12)	C(13)	C(14)	0.5(7)	O(24)	C(19)	N(18)	C(17)	-168.5(4)

Table 29S. Bond lengths for **10o**.

Atom	Atom	Length/Å	Atom	Atom	Length/Å
C(1)	C(2)	1.381(2)	C(9)	C(10)	1.386(2)
C(1)	C(16)	1.3982(19)	C(10)	C(11)	1.388(2)
C(2)	C(3)	1.389(2)	C(11)	C(12)	1.3850(19)
C(3)	C(4)	1.390(2)	C(12)	C(13)	1.3958(19)
C(4)	C(5)	1.389(2)	C(13)	N(14)	1.4346(17)
C(5)	C(16)	1.3930(19)	C(15)	C(16)	1.4947(19)
C(5)	N(6)	1.4502(17)	C(15)	N(14)	1.3630(17)
C(7)	C(8)	1.4979(18)	C(15)	O(22)	1.2282(17)
C(7)	N(6)	1.4006(17)	C(17)	C(18)	1.498(2)
C(7)	O(20)	1.2112(17)	C(17)	N(6)	1.4256(17)
C(8)	C(9)	1.3982(19)	C(17)	O(19)	1.2087(17)
C(8)	C(13)	1.3949(19)	C(21)	N(14)	1.4674(18)

Table 30S. Values of valence angles for **10o**.

Atom	Atom	Atom	Angle/°	Atom	Atom	Atom	Angle/°
C(2)	C(1)	C(16)	120.24(14)	C(8)	C(13)	N(14)	121.12(11)
C(1)	C(2)	C(3)	120.38(13)	C(12)	C(13)	N(14)	119.04(12)
C(2)	C(3)	C(4)	119.98(14)	N(14)	C(15)	C(16)	117.84(11)
C(5)	C(4)	C(3)	119.61(14)	O(22)	C(15)	C(16)	120.64(12)
C(4)	C(5)	C(16)	120.71(13)	O(22)	C(15)	N(14)	121.50(13)
C(4)	C(5)	N(6)	119.83(12)	C(1)	C(16)	C(15)	117.93(12)
C(16)	C(5)	N(6)	119.26(12)	C(5)	C(16)	C(1)	119.05(13)
N(6)	C(7)	C(8)	117.50(11)	C(5)	C(16)	C(15)	122.92(12)
O(20)	C(7)	C(8)	120.12(12)	N(6)	C(17)	C(18)	119.13(12)
O(20)	C(7)	N(6)	122.37(12)	O(19)	C(17)	C(18)	121.68(12)
C(9)	C(8)	C(7)	117.23(12)	O(19)	C(17)	N(6)	119.12(12)
C(13)	C(8)	C(7)	122.80(12)	C(7)	N(6)	C(5)	120.44(11)
C(13)	C(8)	C(9)	119.55(12)	C(7)	N(6)	C(17)	123.55(11)
C(10)	C(9)	C(8)	120.48(13)	C(17)	N(6)	C(5)	115.57(11)
C(9)	C(10)	C(11)	119.76(12)	C(13)	N(14)	C(21)	117.88(11)
C(12)	C(11)	C(10)	120.28(12)	C(15)	N(14)	C(13)	123.23(11)
C(11)	C(12)	C(13)	120.30(13)	C(15)	N(14)	C(21)	116.26(11)
C(8)	C(13)	C(12)	119.63(12)				

Table 31S. Values of valence angles for **10o**.

A	B	C	D	Angle/°	A	B	C	D	Angle/°
C(1)	C(2)	C(3)	C(4)	1.2(2)	C(12)	C(13)	N(14)	C(21)	-48.75(17)
C(2)	C(1)	C(16)	C(5)	-1.46(19)	C(13)	C(8)	C(9)	C(10)	-0.9(2)
C(2)	C(1)	C(16)	C(15)	-177.98(12)	C(16)	C(1)	C(2)	C(3)	0.1(2)
C(2)	C(3)	C(4)	C(5)	-1.2(2)	C(16)	C(5)	N(6)	C(7)	-79.20(16)
C(3)	C(4)	C(5)	C(16)	-0.22(19)	C(16)	C(5)	N(6)	C(17)	108.10(14)
C(3)	C(4)	C(5)	N(6)	174.45(12)	C(16)	C(15)	N(14)	C(13)	8.03(18)
C(4)	C(5)	C(16)	C(1)	1.52(18)	C(16)	C(15)	N(14)	C(21)	169.20(11)
C(4)	C(5)	C(16)	C(15)	177.86(12)	C(18)	C(17)	N(6)	C(5)	-169.62(12)
C(4)	C(5)	N(6)	C(7)	106.06(14)	C(18)	C(17)	N(6)	C(7)	17.9(2)
C(4)	C(5)	N(6)	C(17)	-66.65(16)	N(6)	C(5)	C(16)	C(1)	-173.18(11)
C(7)	C(8)	C(9)	C(10)	-173.61(12)	N(6)	C(5)	C(16)	C(15)	3.16(18)
C(7)	C(8)	C(13)	C(12)	173.09(12)	N(6)	C(7)	C(8)	C(9)	-125.40(13)

C(7)	C(8)	C(13)	N(14)	-1.65(19)	N(6)	C(7)	C(8)	C(13)	62.13(17)
C(8)	C(7)	N(6)	C(5)	12.64(18)	N(14)	C(15)	C(16)	C(1)	-120.74(13)
C(8)	C(7)	N(6)	C(17)	-175.26(12)	N(14)	C(15)	C(16)	C(5)	62.88(16)
C(8)	C(9)	C(10)	C(11)	0.4(2)	O(19)	C(17)	N(6)	C(5)	7.24(19)
C(8)	C(13)	N(14)	C(15)	-73.10(17)	O(19)	C(17)	N(6)	C(7)	-165.21(13)
C(8)	C(13)	N(14)	C(21)	126.02(14)	O(20)	C(7)	C(8)	C(9)	53.62(18)
C(9)	C(8)	C(13)	C(12)	0.79(19)	O(20)	C(7)	C(8)	C(13)	-118.84(15)
C(9)	C(8)	C(13)	N(14)	-173.94(12)	O(20)	C(7)	N(6)	C(5)	-166.36(13)
C(9)	C(10)	C(11)	C(12)	0.3(2)	O(20)	C(7)	N(6)	C(17)	5.7(2)
C(10)	C(11)	C(12)	C(13)	-0.3(2)	O(22)	C(15)	C(16)	C(1)	57.54(17)
C(11)	C(12)	C(13)	C(8)	-0.2(2)	O(22)	C(15)	C(16)	C(5)	-118.83(14)
C(11)	C(12)	C(13)	N(14)	174.66(12)	O(22)	C(15)	N(14)	C(13)	-170.24(12)
C(12)	C(13)	N(14)	C(15)	112.14(14)	O(22)	C(15)	N(14)	C(21)	-9.07(18)

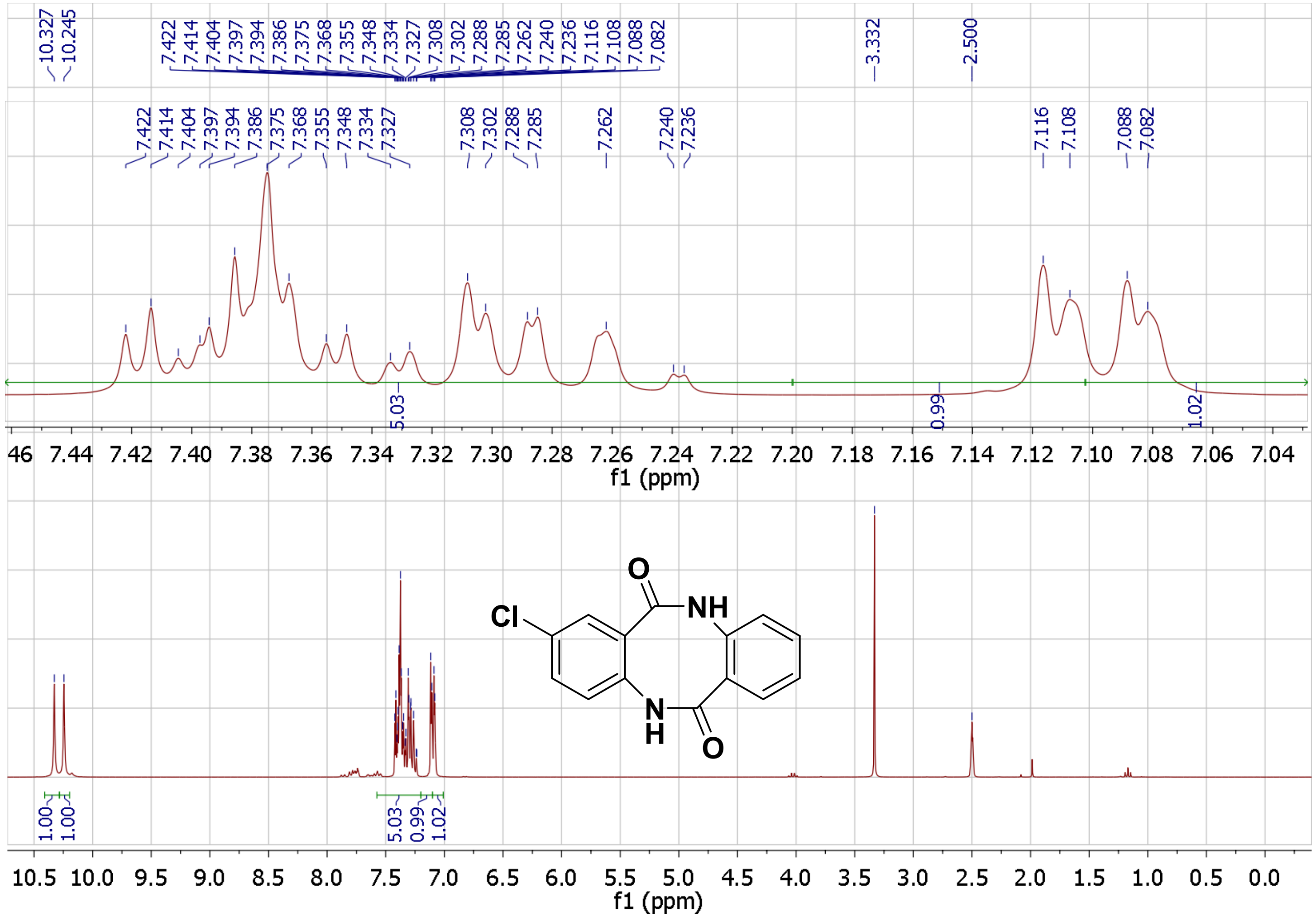

Figure 11S. 1H NMR spectrum of 2-chlorodibenzo[*b*,*f*][1,5]diazocine-6,12(5*H*,11*H*)-dione (**10a**)

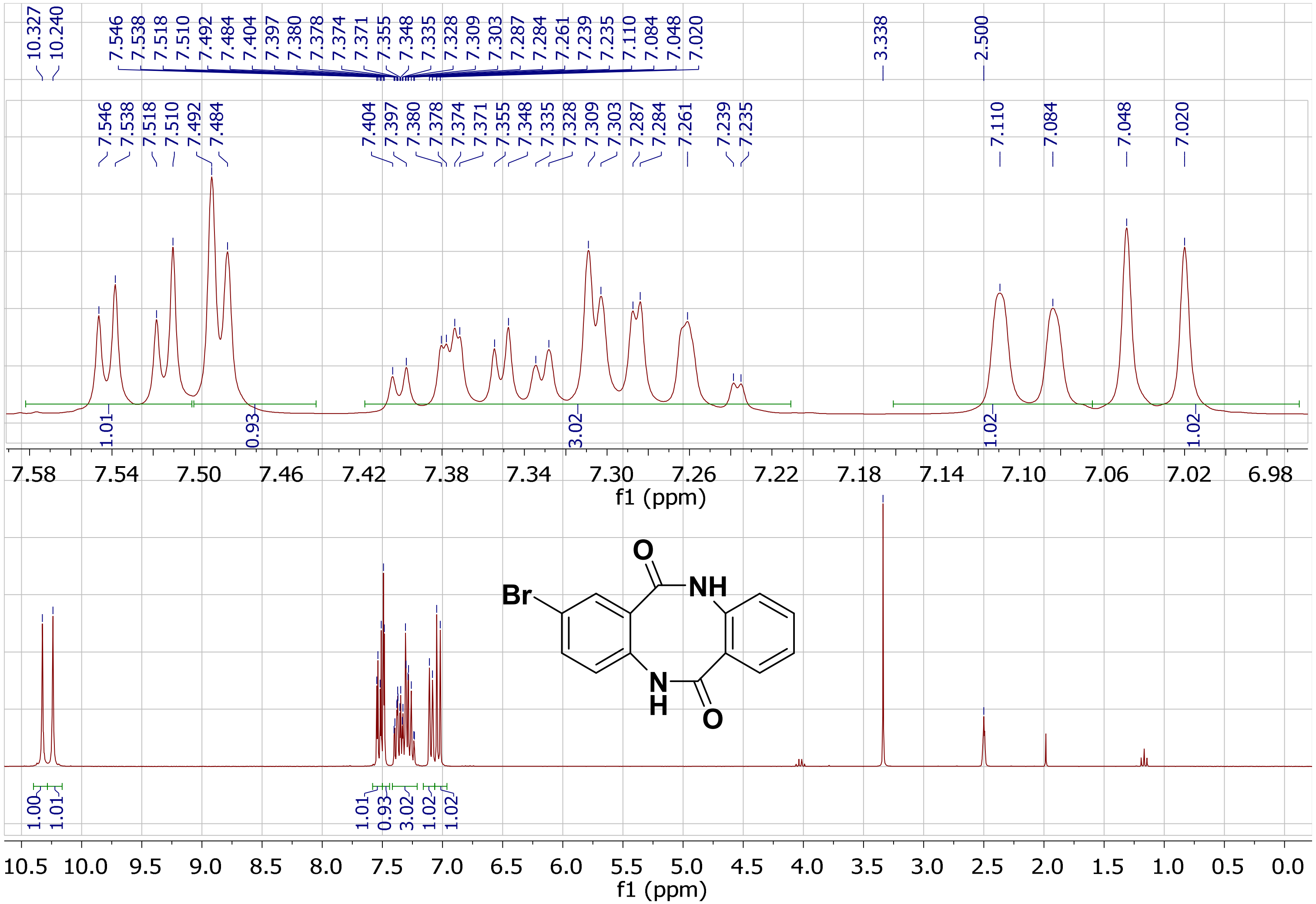

Figure 12S. 1H NMR spectrum of 2-bromodibenzo[*b*,*f*][1,5]diazocine-6,12(5*H*,11*H*)-dione (**10b**)

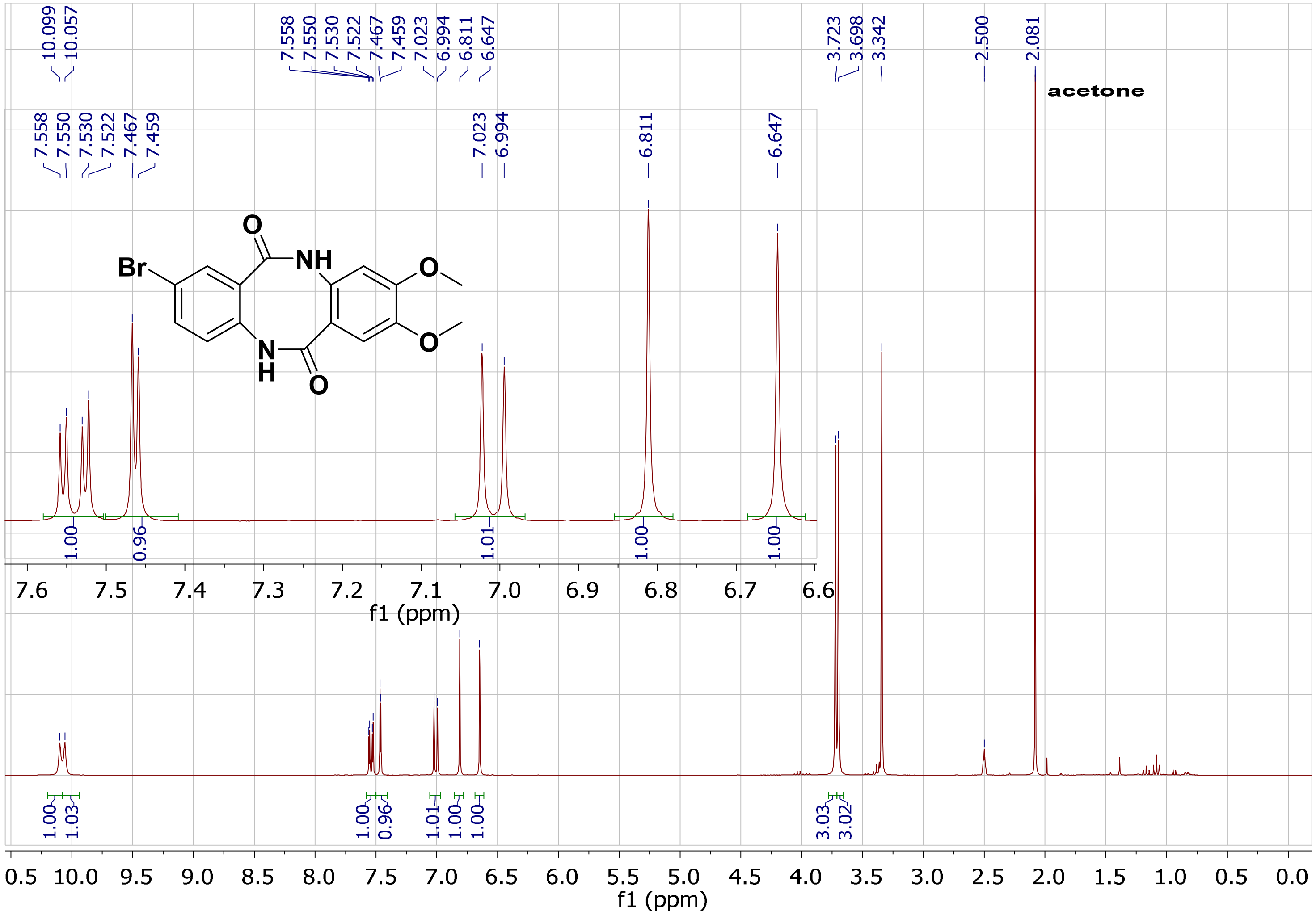

Figure 13S. 1H NMR spectrum of 8-bromo-2,3-dimethoxydibenzo[*b*,*f*][1,5]diazocine-6,12(5*H*,11*H*)-dione (**10c**)

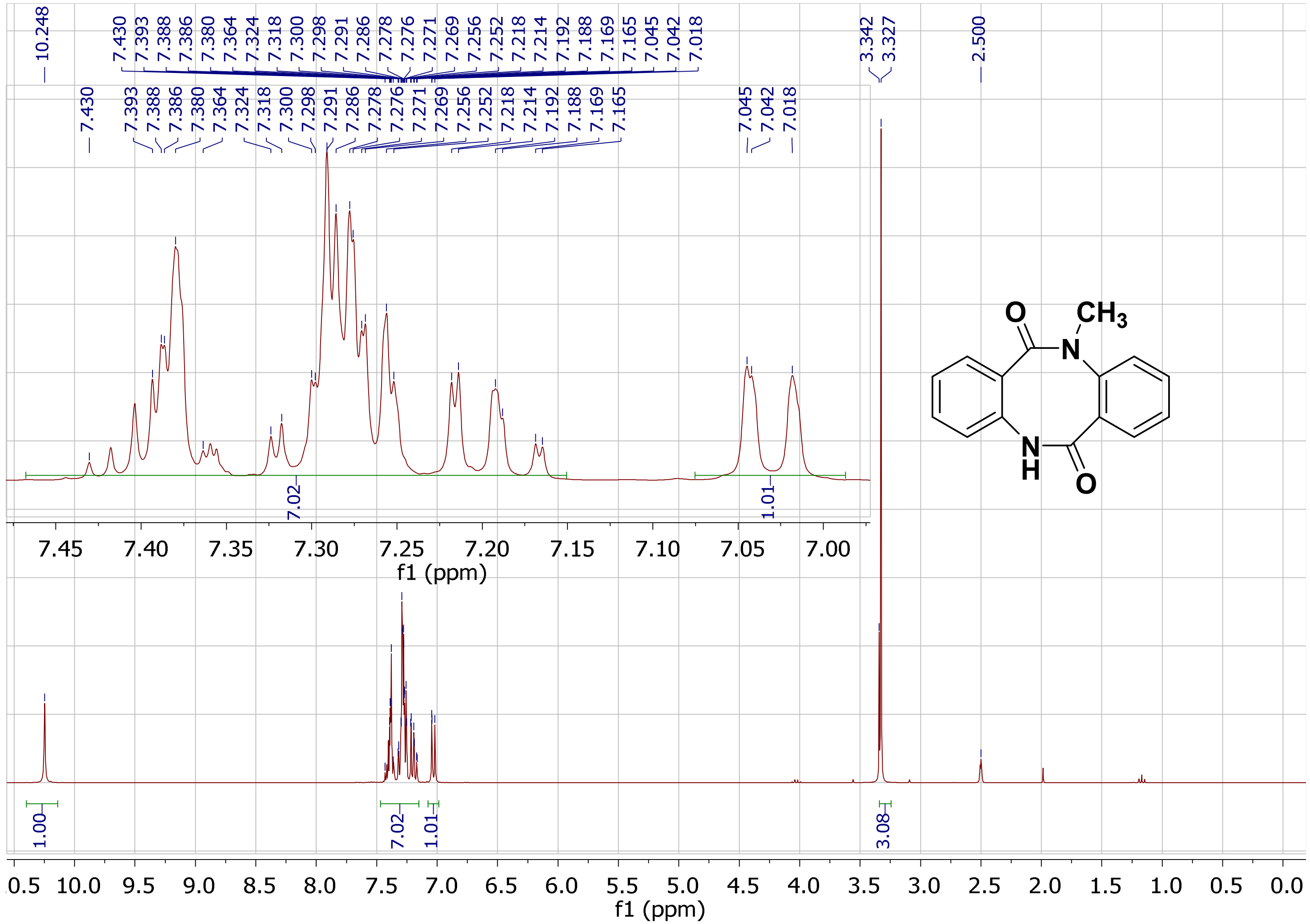

Figure 14S. 1H NMR spectrum of 5-methyldibenzo[*b*,*f*][1,5]diazocine-6,12(5*H*,11*H*)-dione (**10d**)

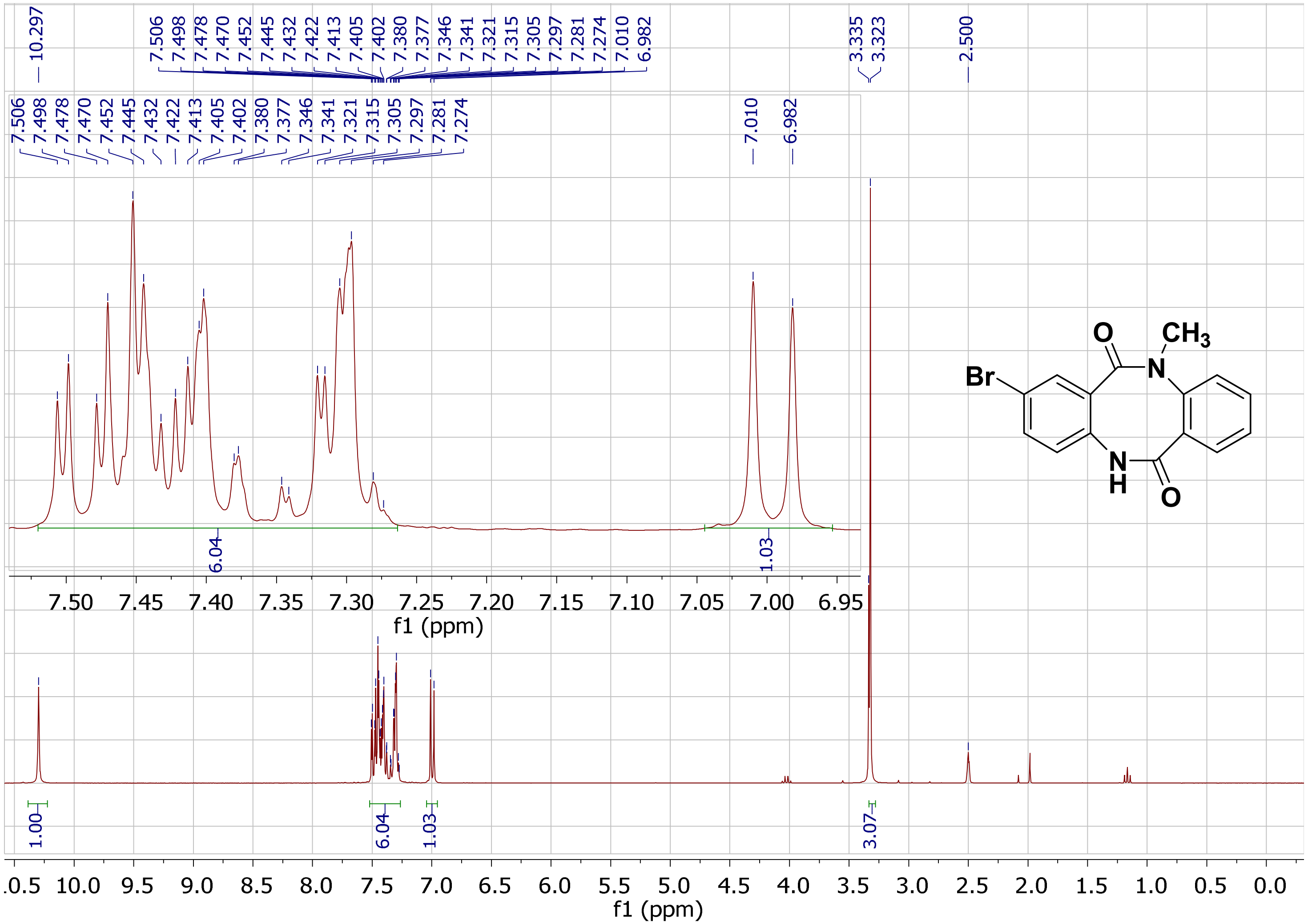

Figure 15S. 1H NMR spectrum of 2-bromo-11-methyldibenzo[*b*,*f*][1,5]diazocine-6,12(5*H*,11*H*)-dione (**10e**)

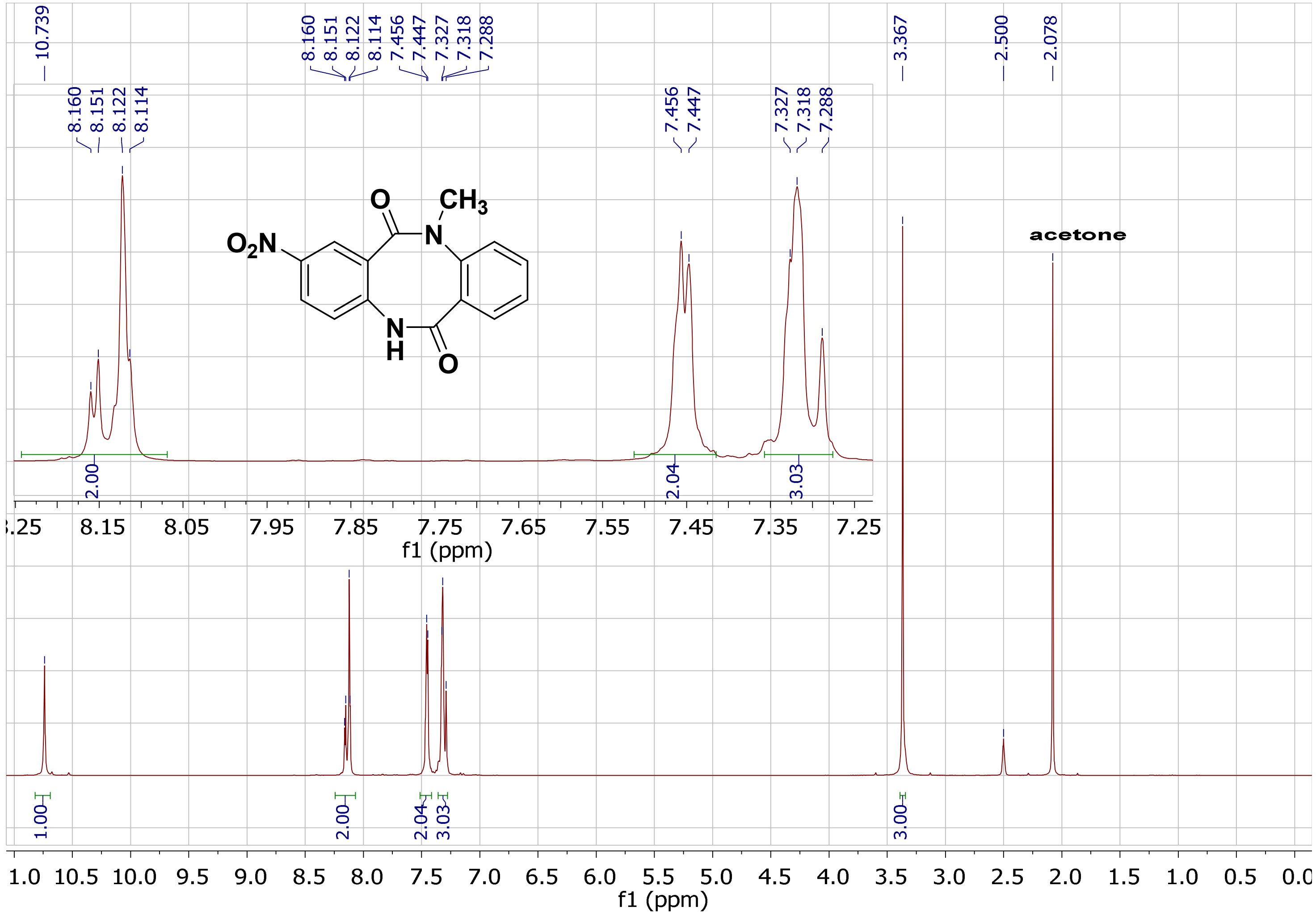

Figure 16S. 1H NMR spectrum of 11-methyl-2-nitrodibenzo[*b*,*f*][1,5]diazocine-6,12(5*H*,11*H*)-dione (**10f**)

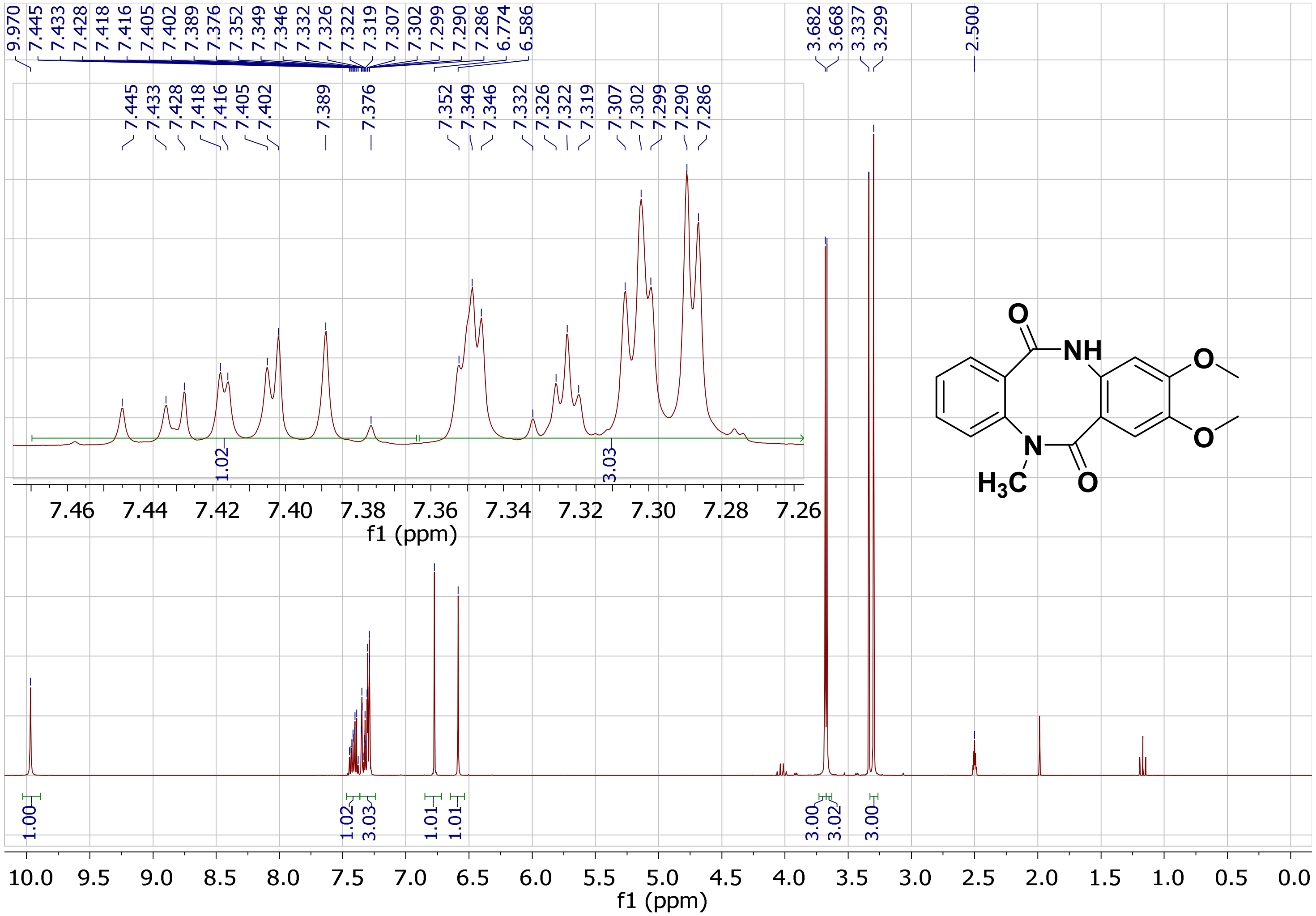

Figure 17S. 1H NMR spectrum of 2,3-dimethoxy-11-methyldibenzo[*b*,*f*][1,5]diazocine-6,12(5*H*,11*H*)-dione (**10g**)

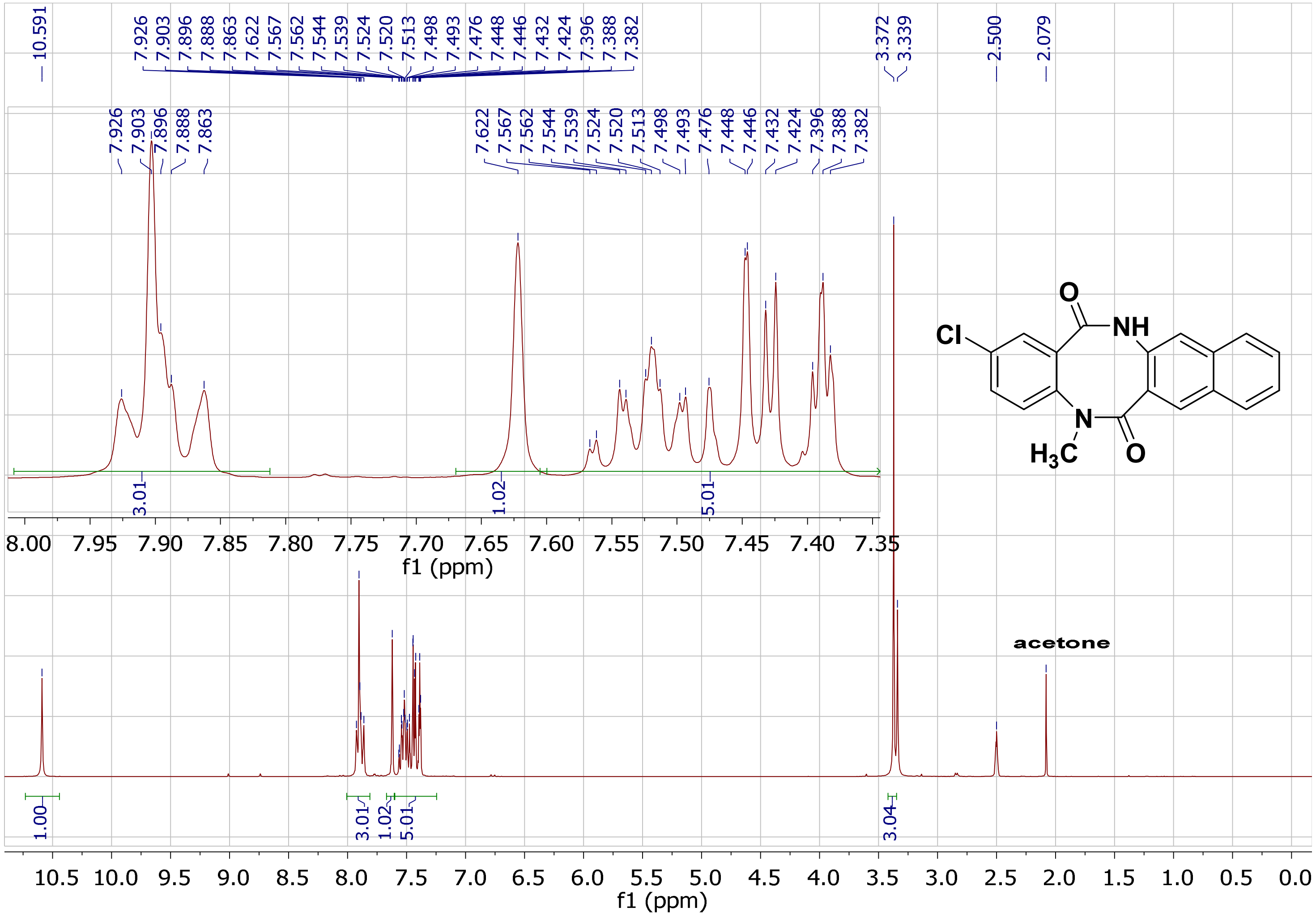

Figure 18S. 1H NMR spectrum of 2-chloro-5-methylbenzo[*b*]naphtho[2,3-*f*][1,5]diazocine-6,14(5*H*,13*H*)-dione (**10h**)

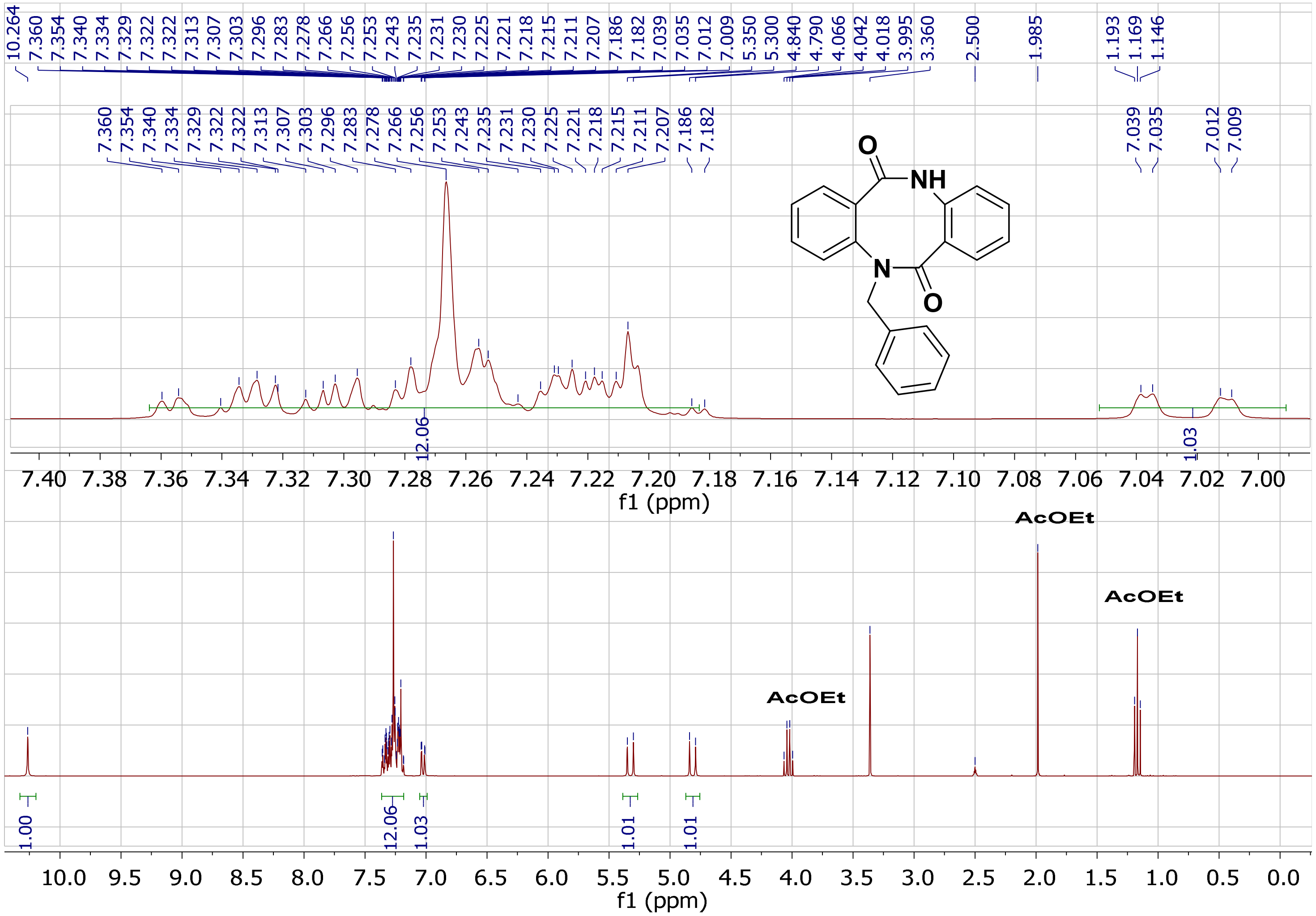

Figure 19S. 1H NMR spectrum of 5-benzyldibenzo[*b*,*f*][1,5]diazocine-6,12(5*H*,11*H*)-dione (**10i**)

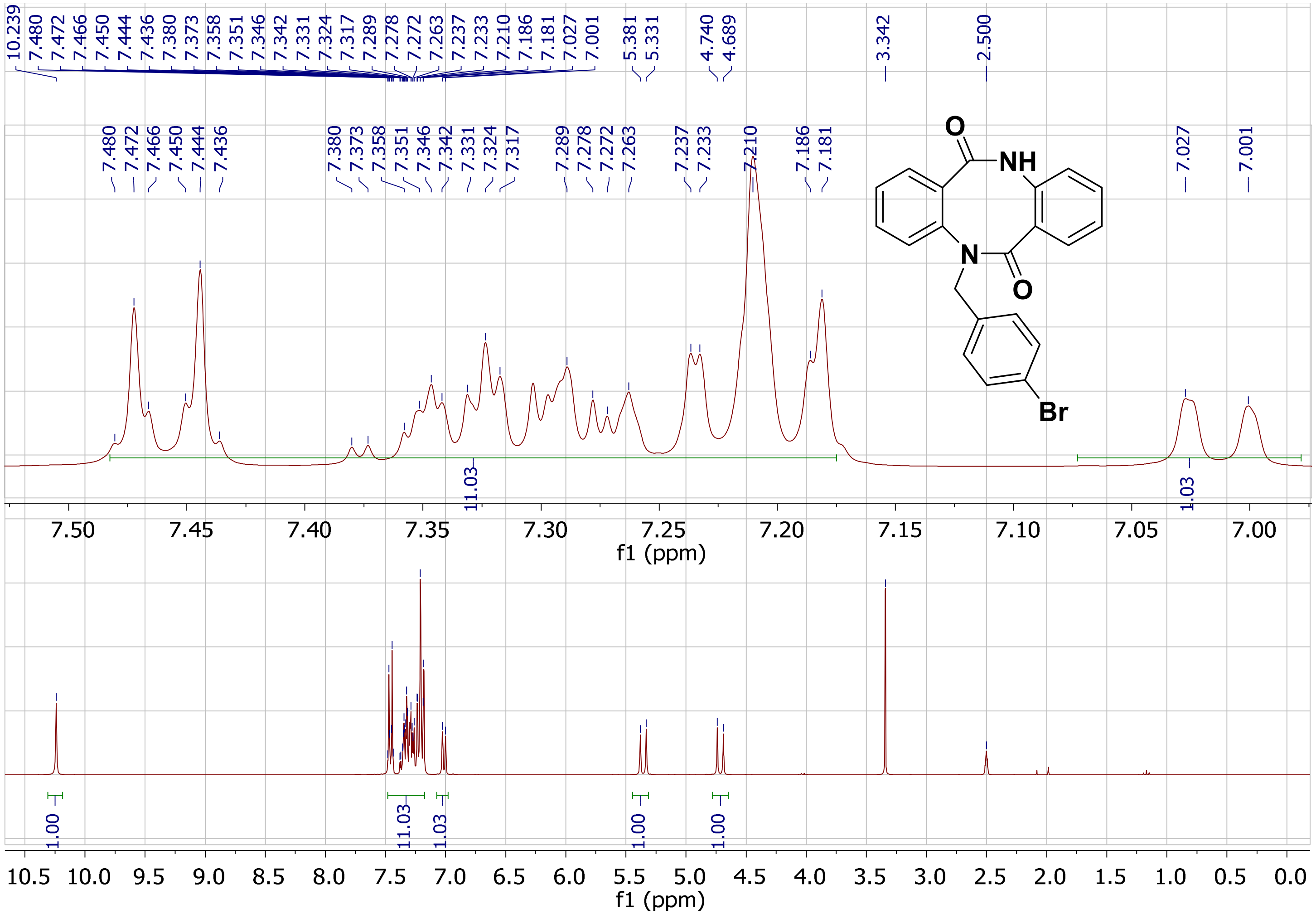

Figure 20S. 1H NMR spectrum of 5-(4-bromobenzyl)dibenzo[*b*,*f*][1,5]diazocine-6,12(5*H*,11*H*)-dione (**10j**)

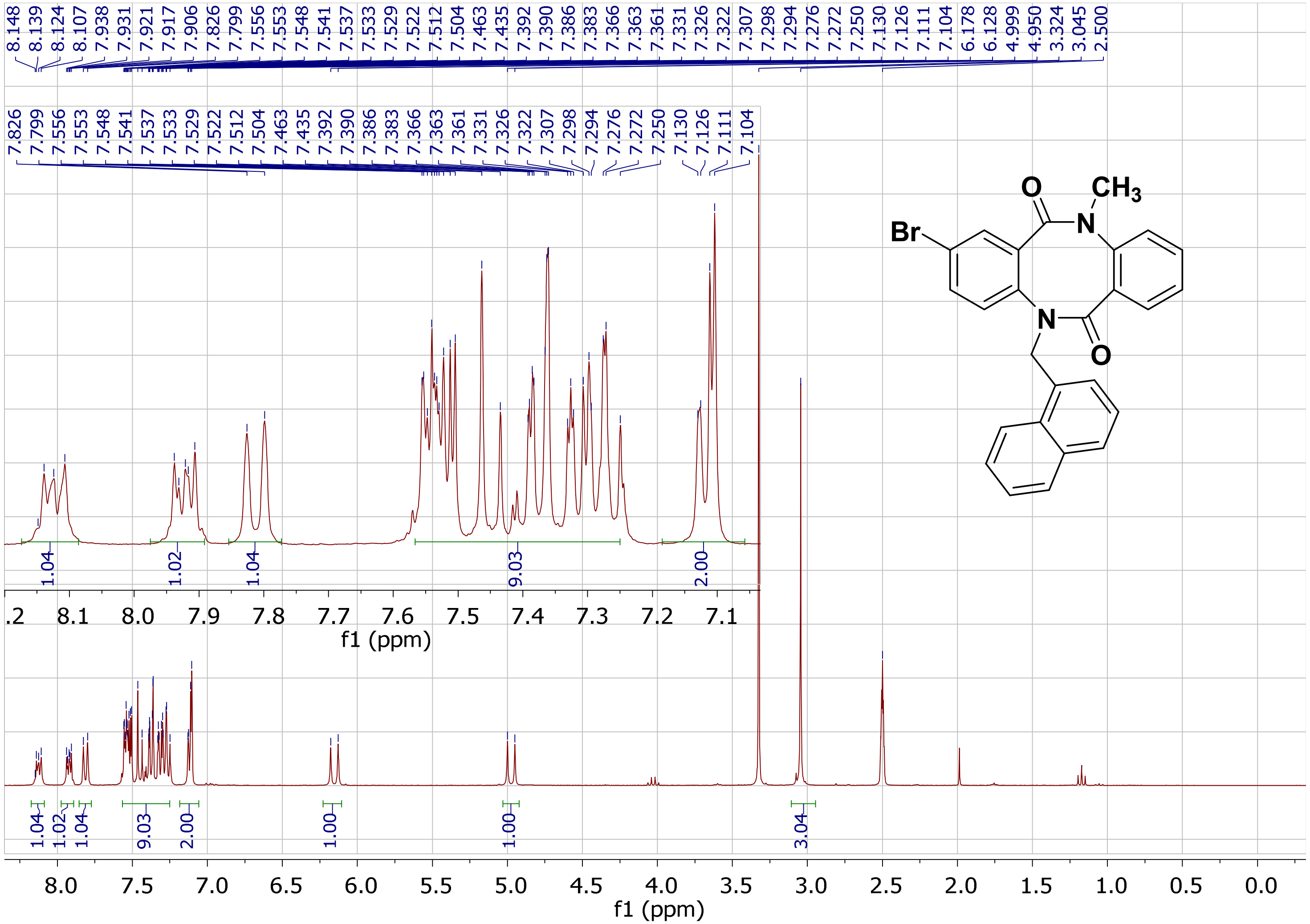

Figure 21S. 1H NMR spectrum of 2-bromo-11-methyl-5-(naphthalen-1-ylmethyl)dibenzo[*b,f*][1,5]diazocine-6,12(5*H*,11*H*)-dione (**10k**)

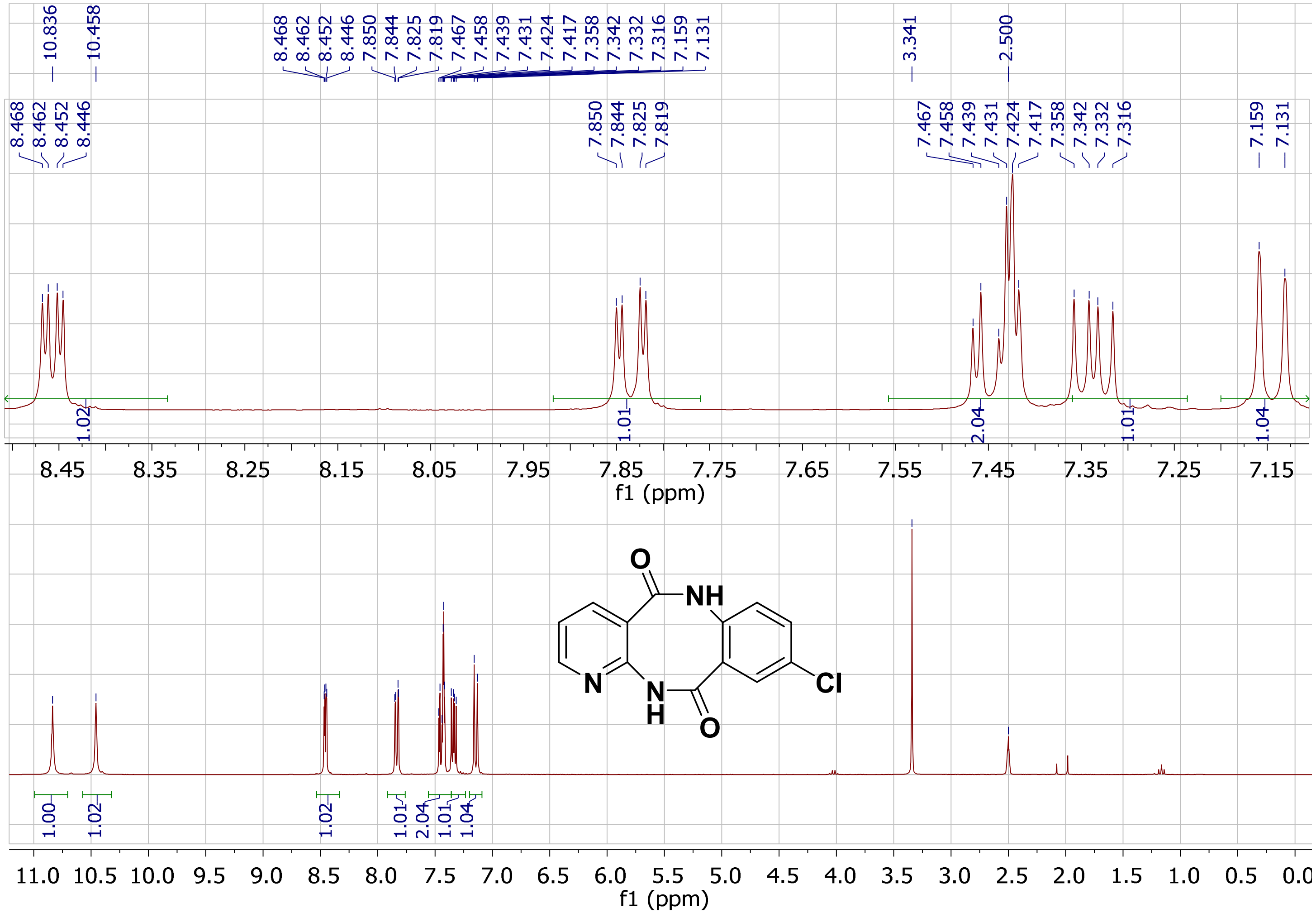

Figure 22S. 1H NMR spectrum of 8-chloropyrido[3,2-*c*][1,5]benzodiazocine-5,11(6*H*,12*H*)-dione (**10l**)

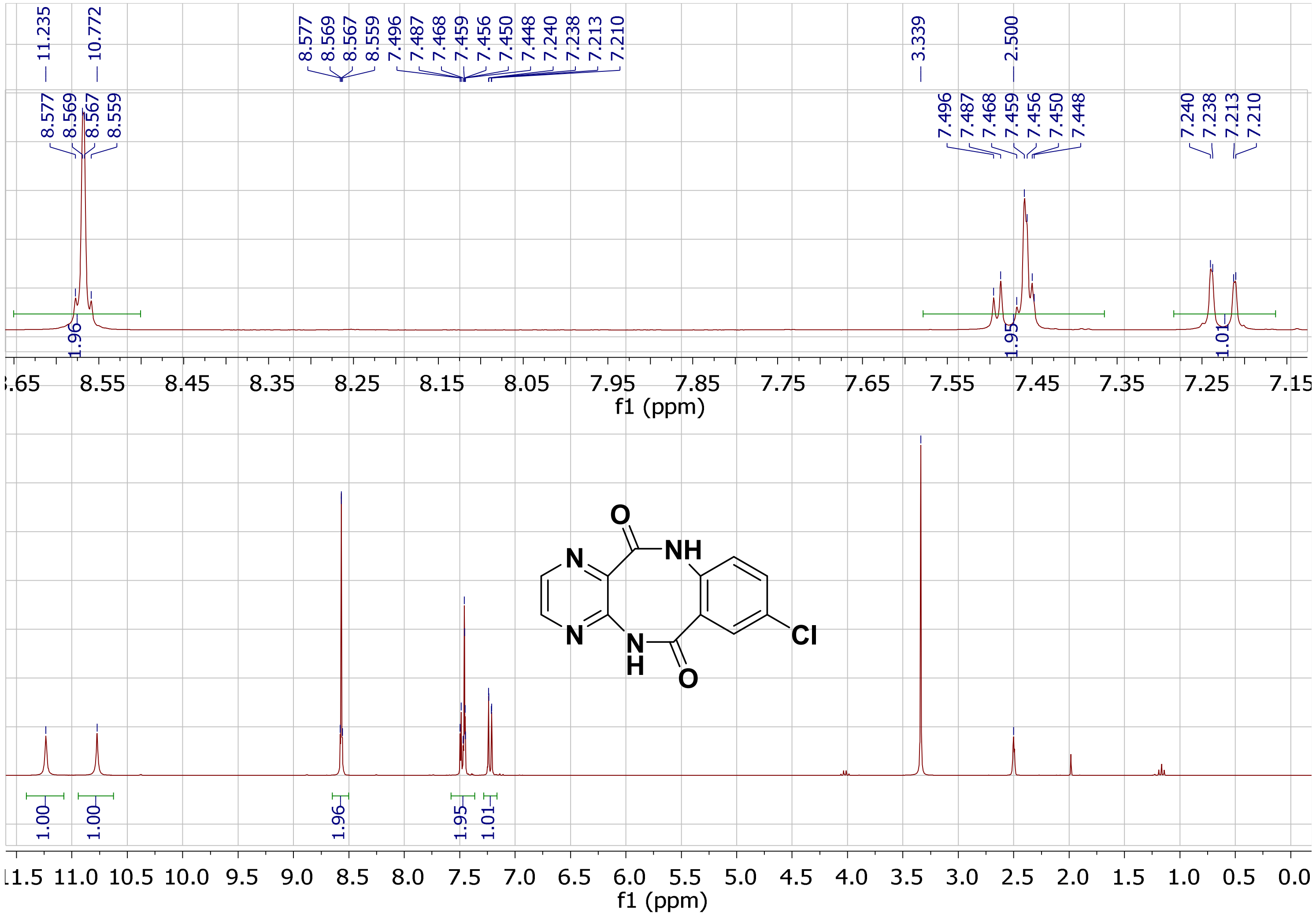

Figure 23S. 1H NMR spectrum of 8-chloropyrazino[3,2-*c*][1,5]benzodiazocine-6,12(5*H*,11*H*)-dione (**10m**)

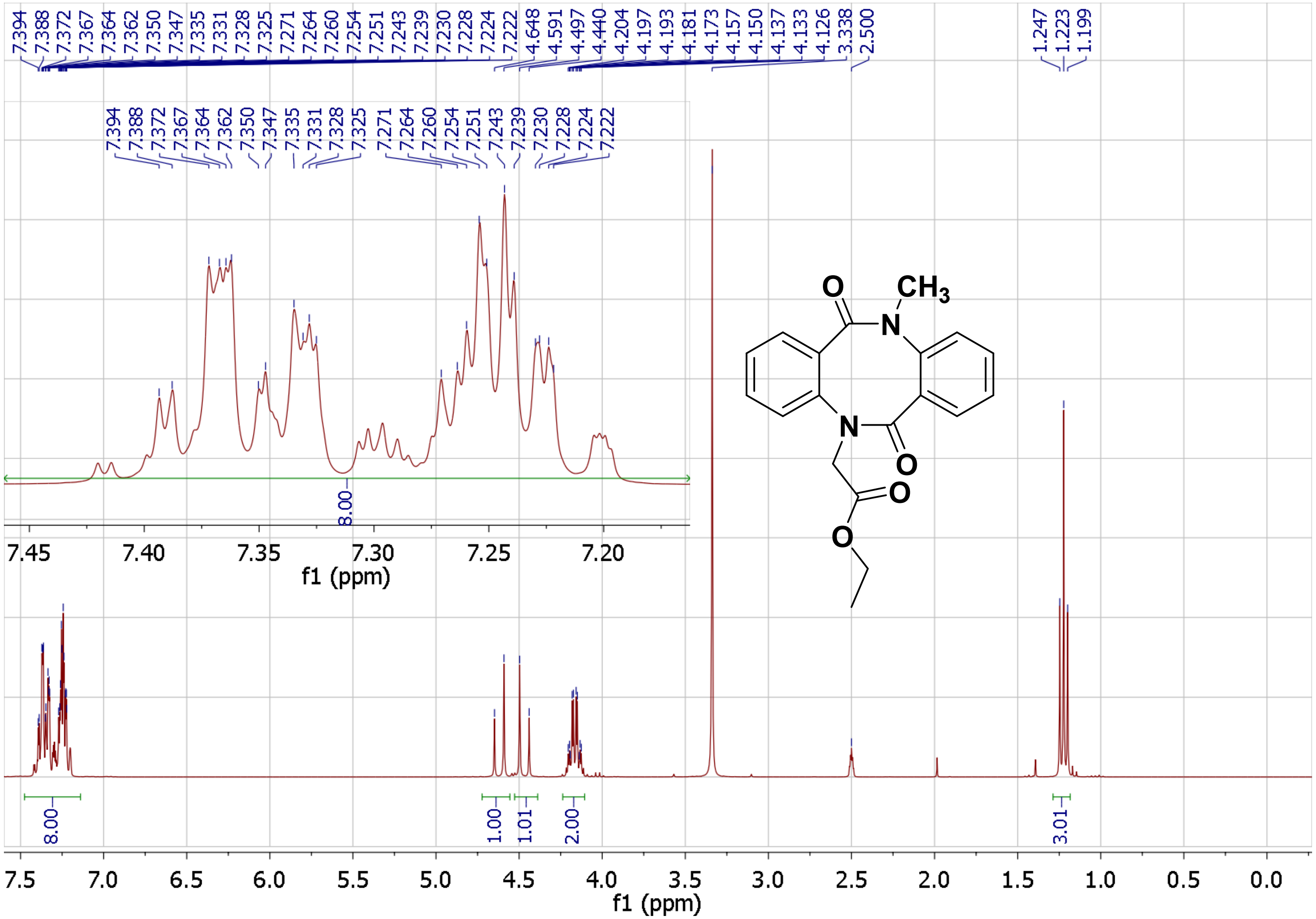

Figure 24S. 1H NMR spectrum of ethyl 2-(11-methyl-6,12-dioxo-11,12-dihydrodibenzo[*b*,*f*][1,5]diazocin-5(6*H*)-yl)acetate (**10n**)

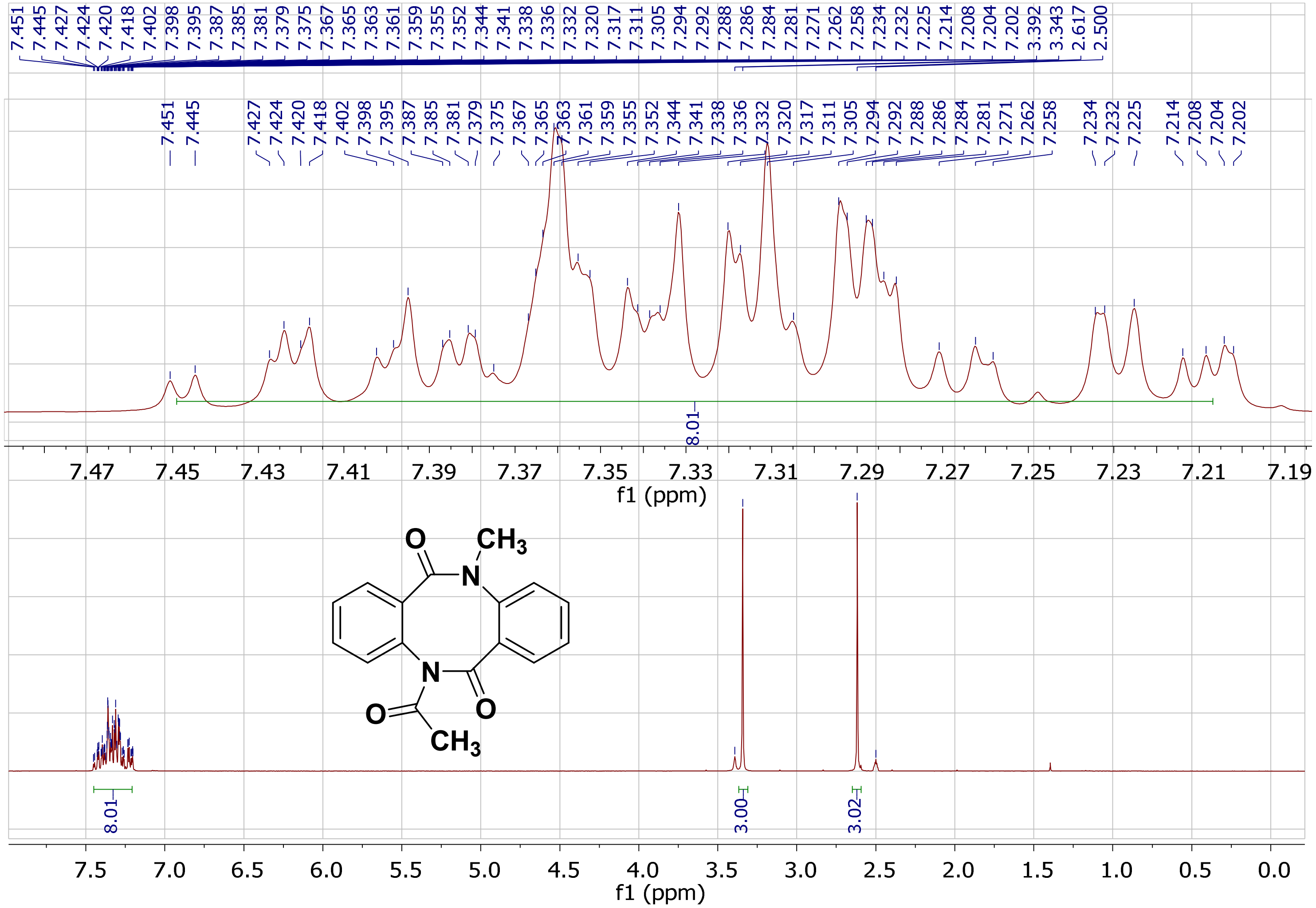

Figure 25S. 1H NMR spectrum of 5-acetyl-11-methyldibenzo[*b*,*f*][1,5]diazocine-6,12(5*H*,11*H*)-dione (**10o**)

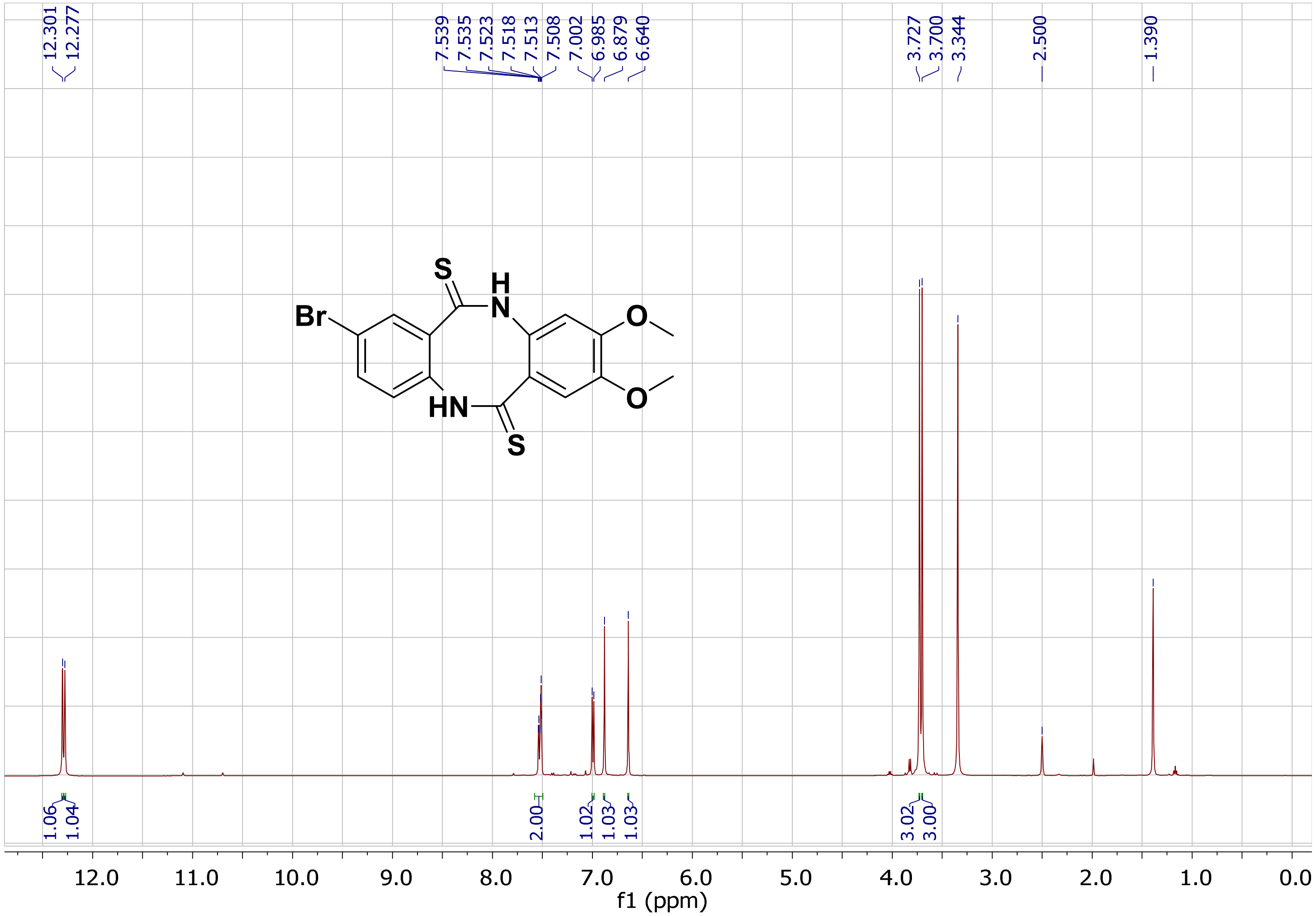

Figure 26S. 1H NMR spectrum of 8-bromo-2,3-dimethoxydibenzo[*b*,*f*][1,5]diazocine-6,12(5*H*,11*H*)-dithione (**10p**)

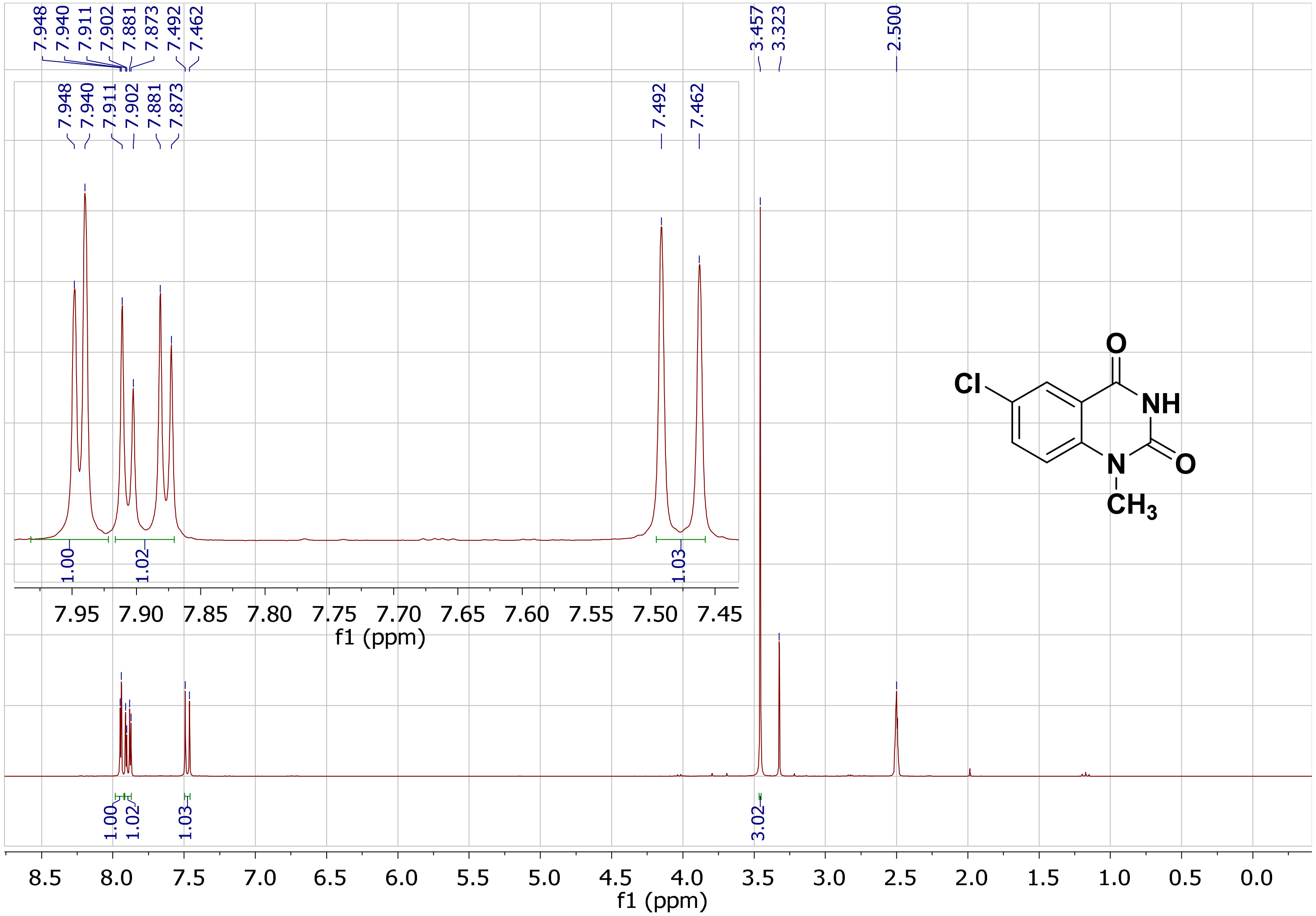

Figure 27S. 1H NMR spectrum of 6-chloro-1-methyl-1*H*-benzo[*d*][1,3]oxazine-2,4-dione (**13f**)

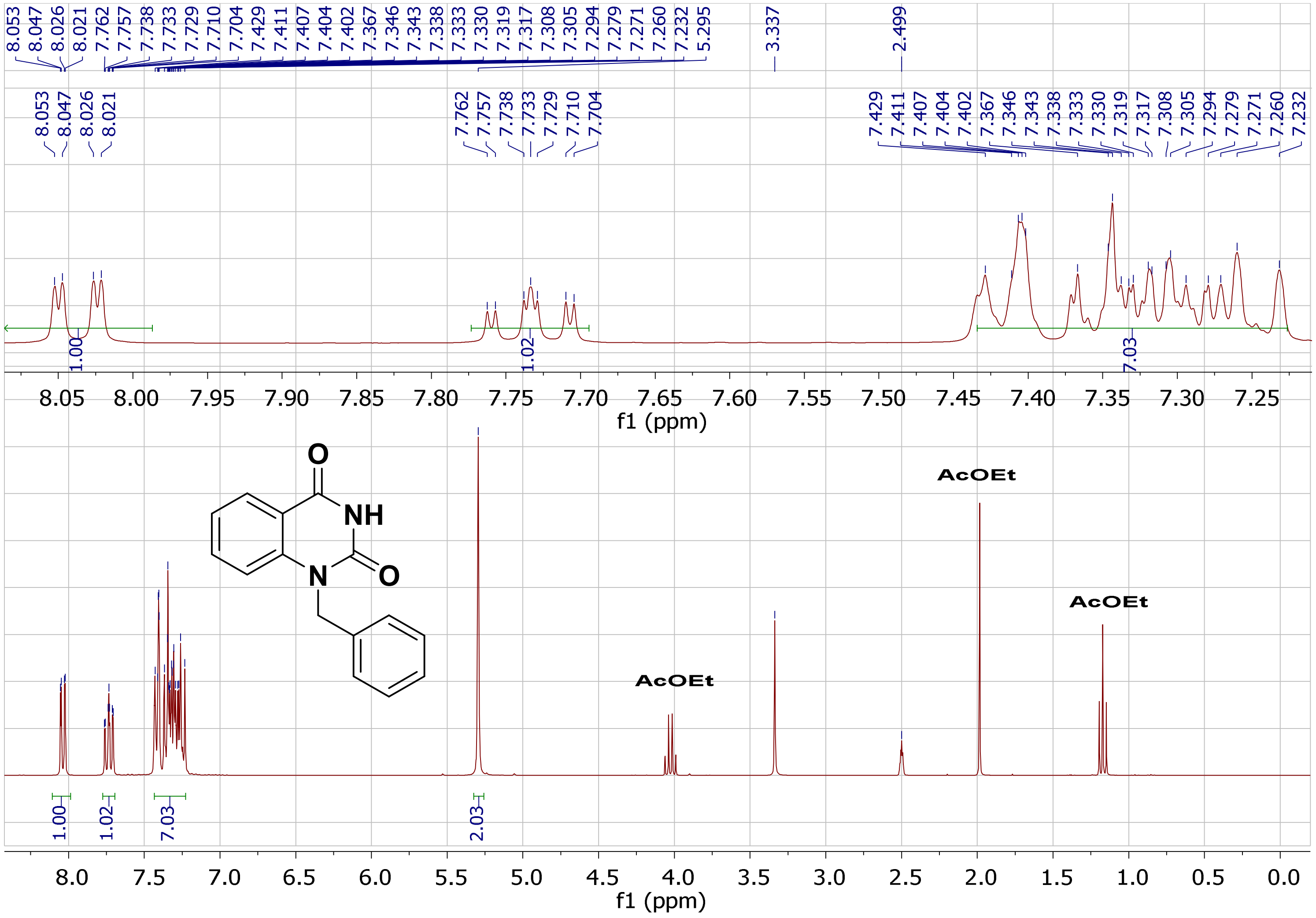

Figure 28S. 1H NMR spectrum of 1-benzyl-1*H*-benzo[*d*][1,3]oxazine-2,4-dione (**13g**)

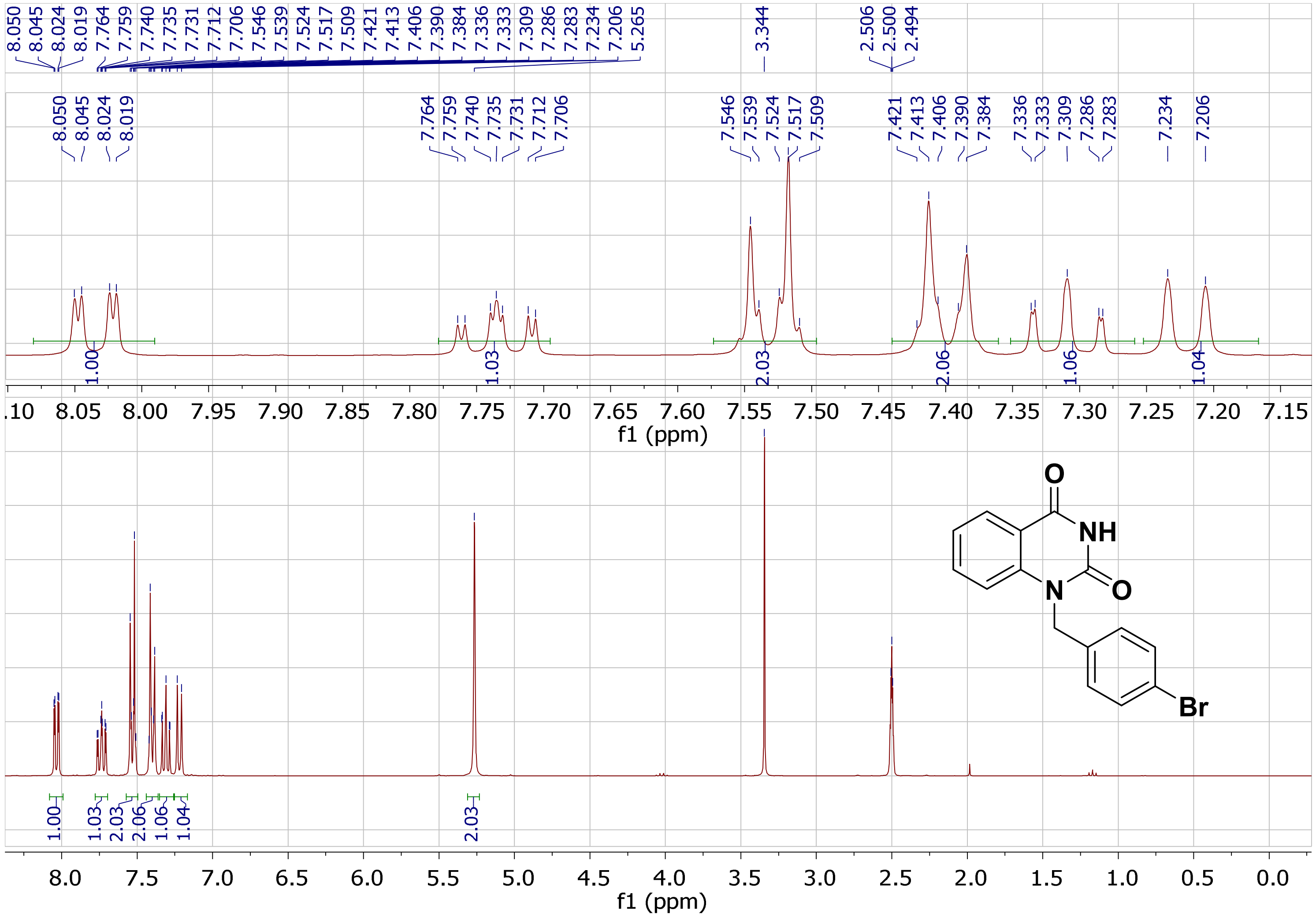

Figure 29S. 1H NMR spectrum of 1-(4-bromobenzyl)-1*H*-benzo[*d*][1,3]oxazine-2,4-dione (**13h**)

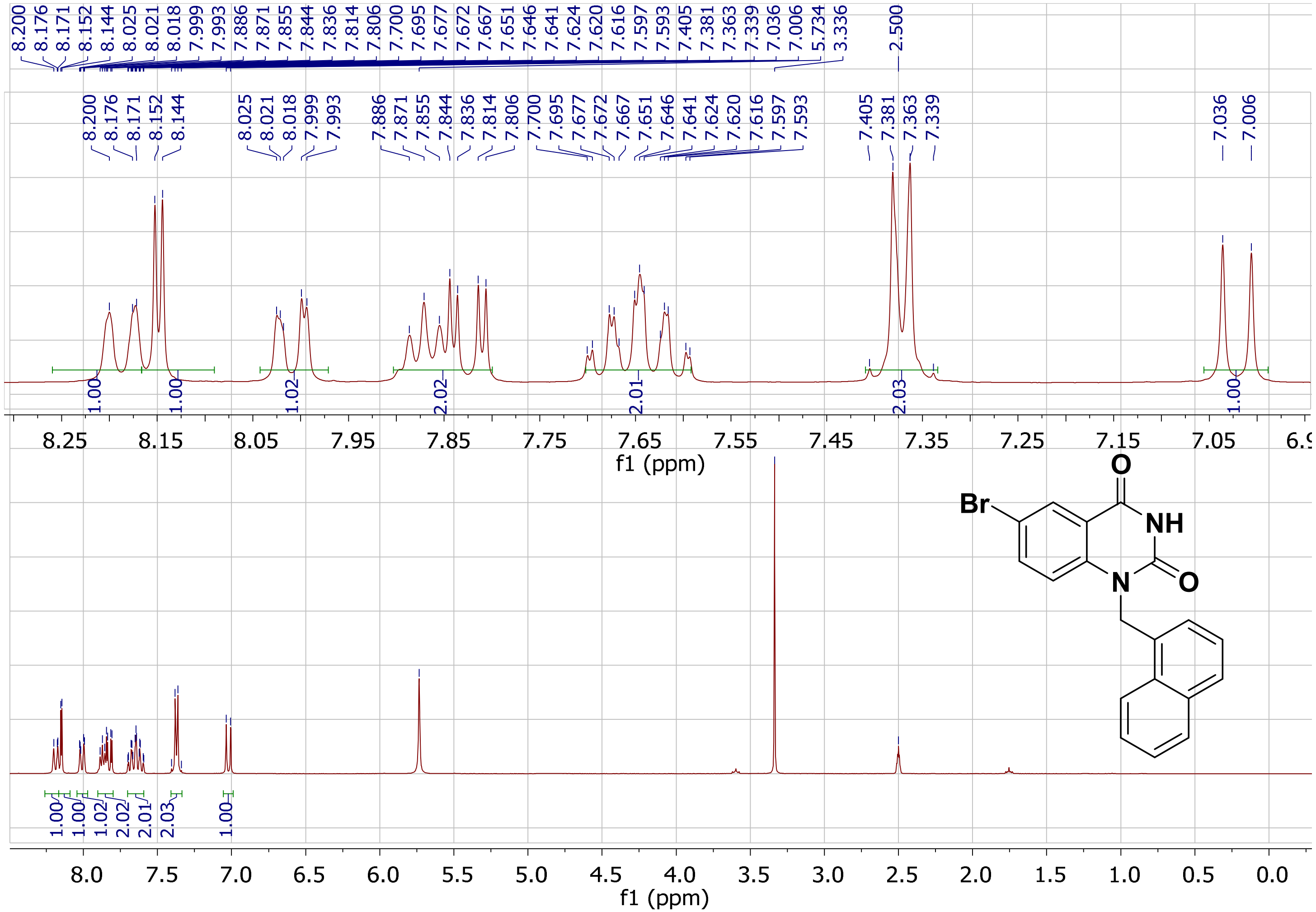

Figure 30S. 1H NMR spectrum of 6-bromo-1-(naphthalen-1-ylmethyl)-1*H*-benzo[*d*][1,3]oxazine-2,4-dione (**13i**)

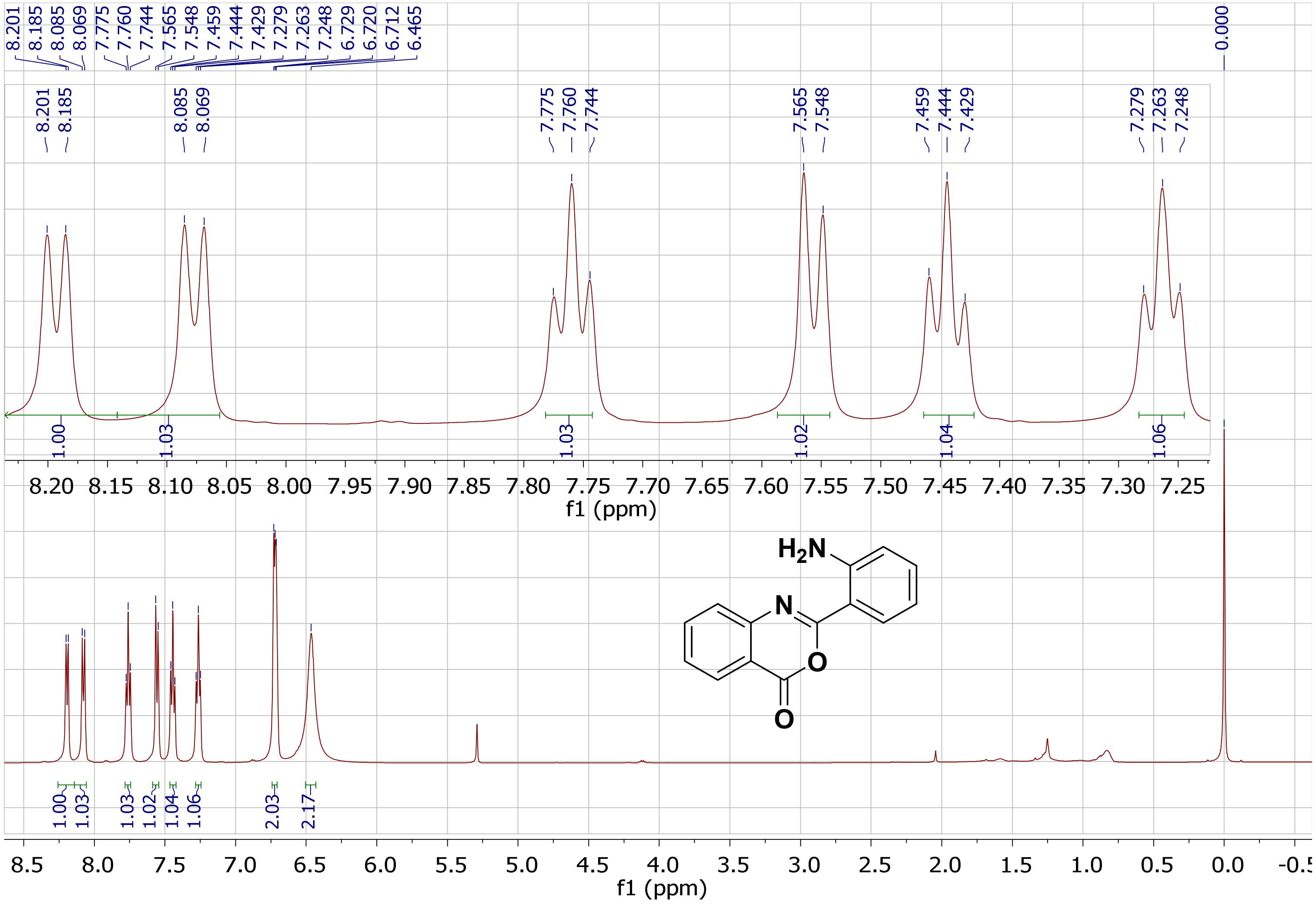

Figure 31S. 1H NMR spectrum of 2-(2-aminophenyl)-4*H*-benzo[*d*][1,3]oxazin-4-one **(12)**

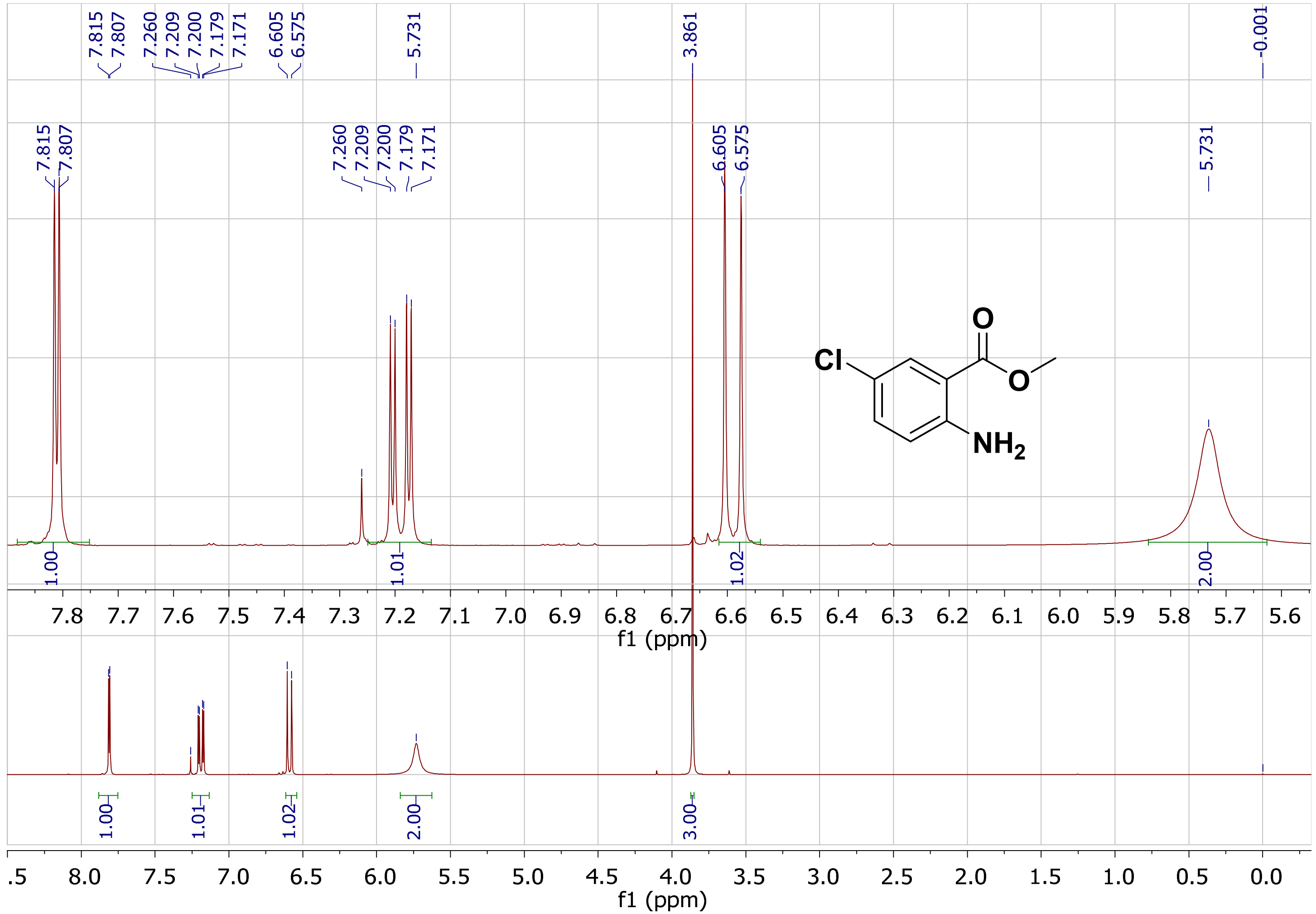

Figure 32S. 1H NMR spectrum of of methyl 2-amino-5-chlorobenzoate (**20**)

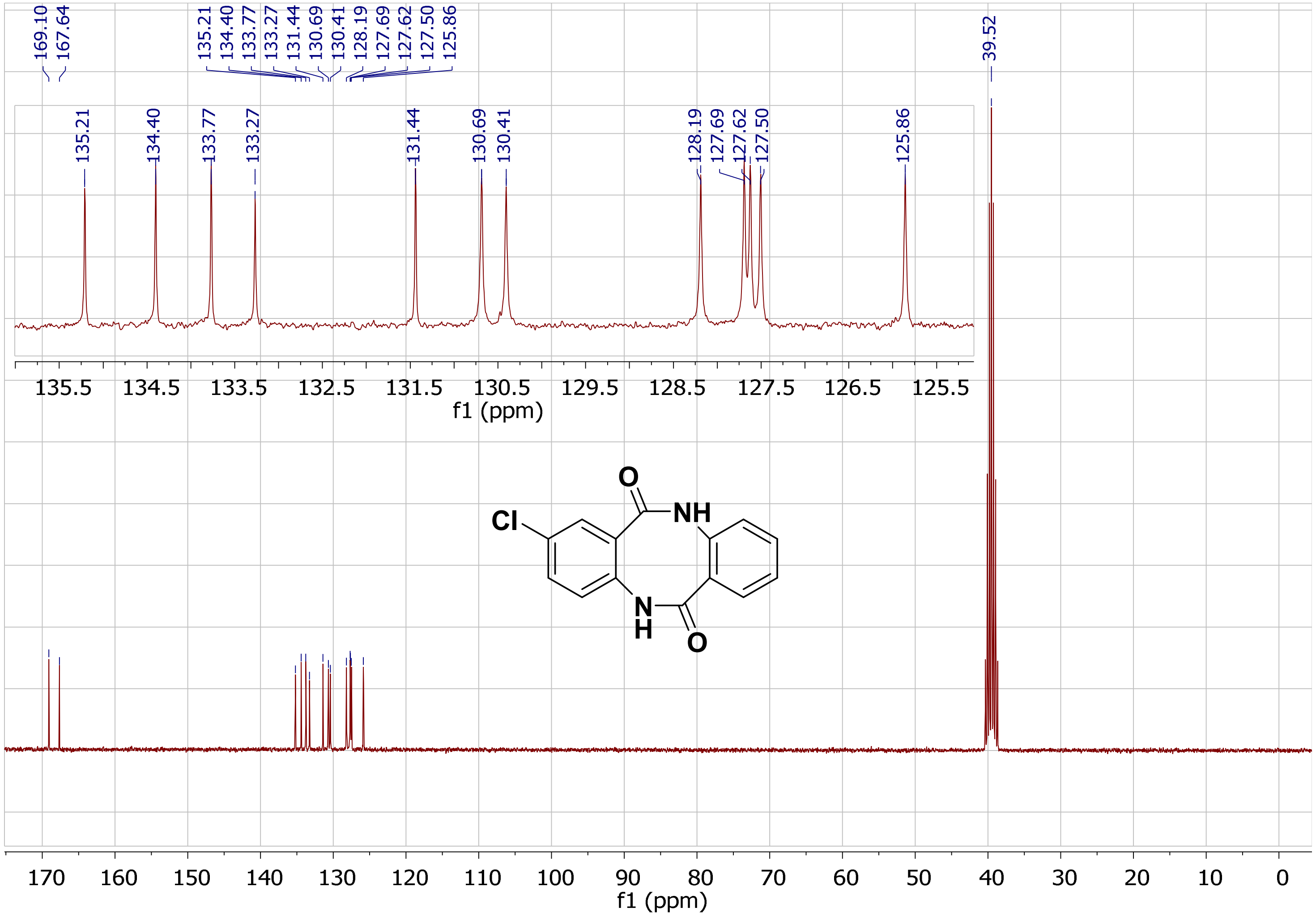

Figure 33S. 13C NMR spectrum of 2-chlorodibenzo[*b,f*][1,5]diazocine-6,12(5*H*,11*H*)-dione (**10a**)

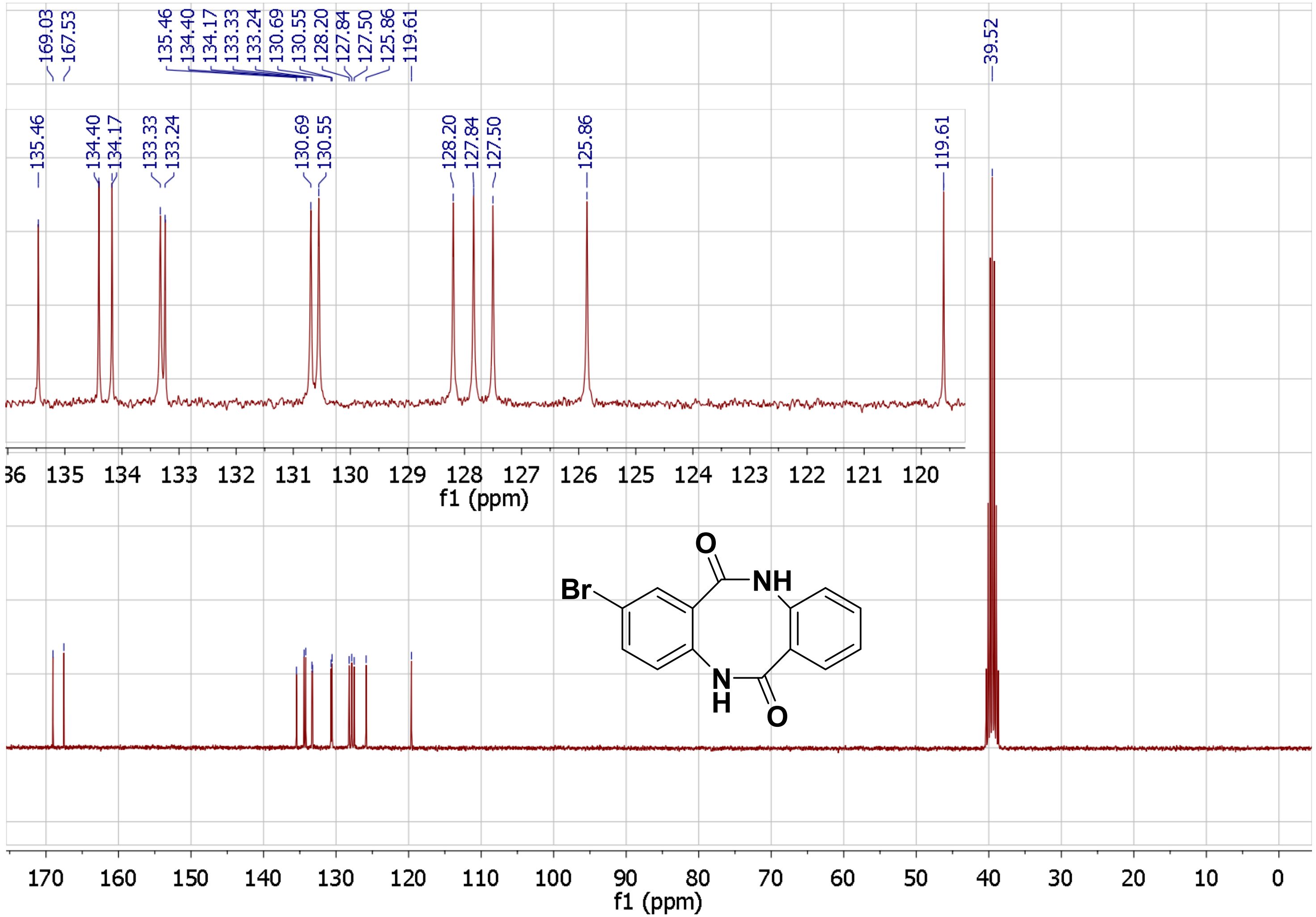

Figure 34S. 13C NMR spectrum of 2-bromodibenzo[*b*,*f*][1,5]diazocine-6,12(5*H*,11*H*)-dione (**10b**)

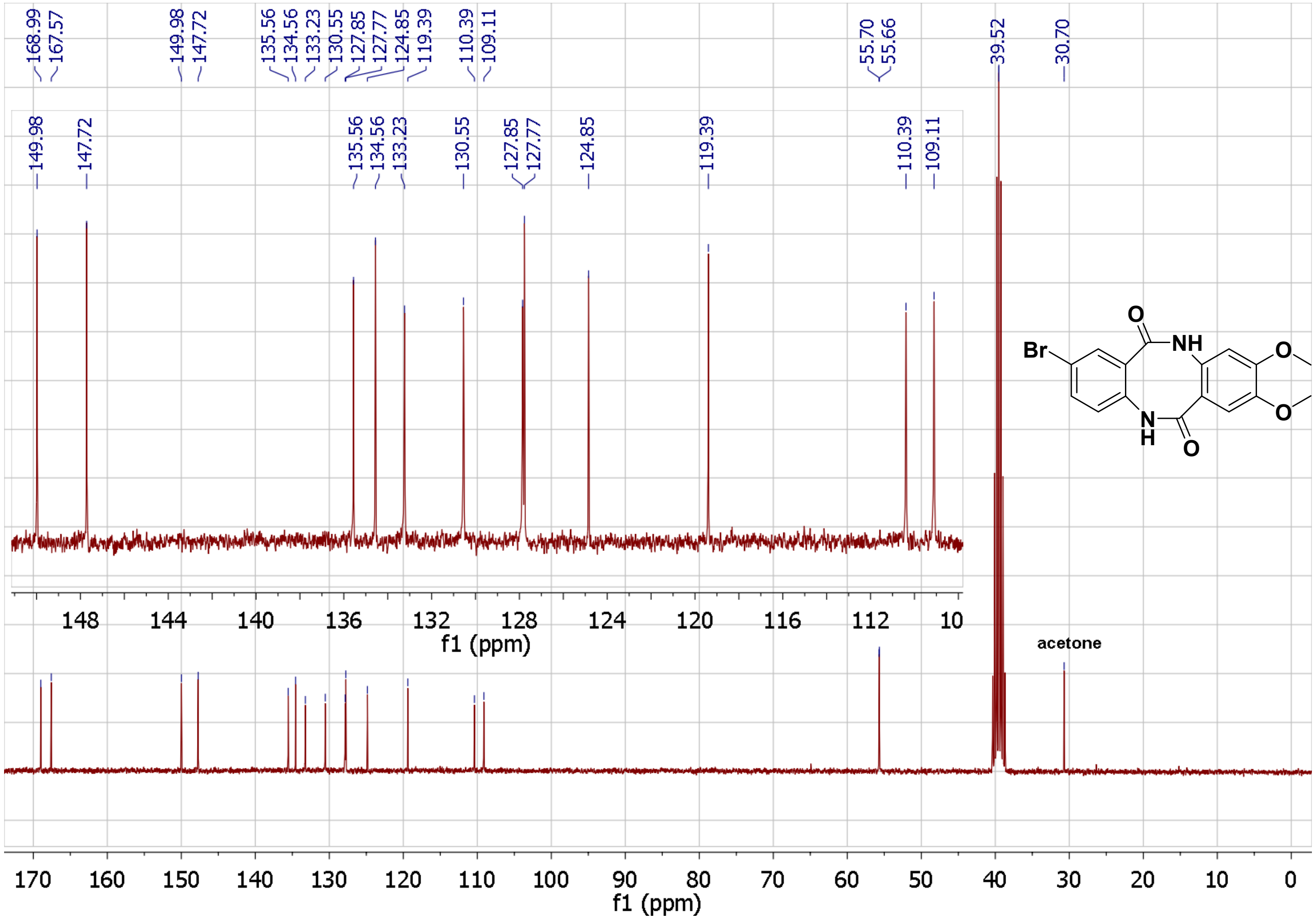

Figure 35S. 13C NMR spectrum of 8-bromo-2,3-dimethoxydibenzo[*b*,*f*][1,5]diazocine-6,12(5*H*,11*H*)-dione (**10c**)

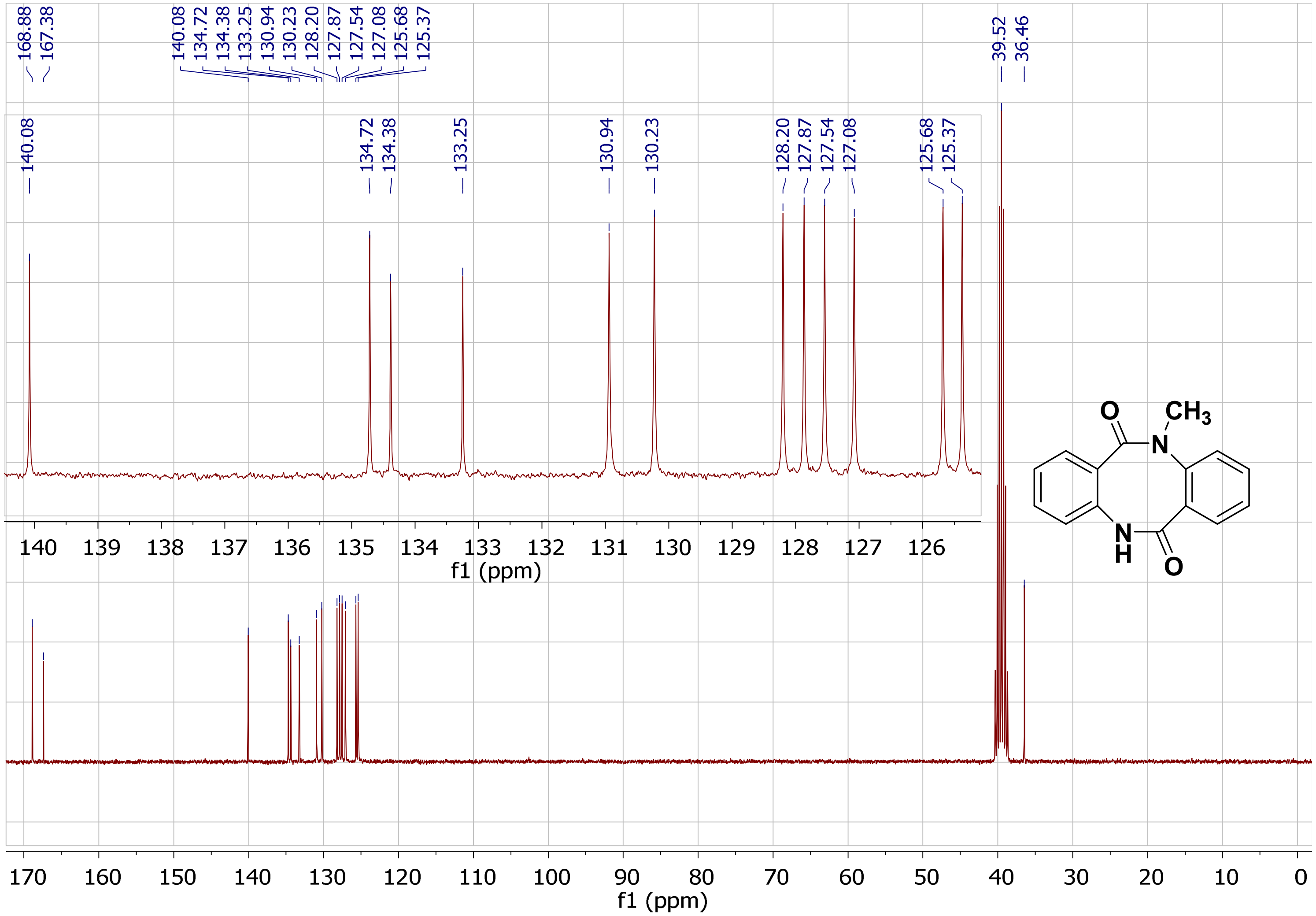

Figure 36S. 13C NMR spectrum of 5-methyldibenzo[*b*,*f*][1,5]diazocine-6,12(5*H*,11*H*)-dione (**10d**)

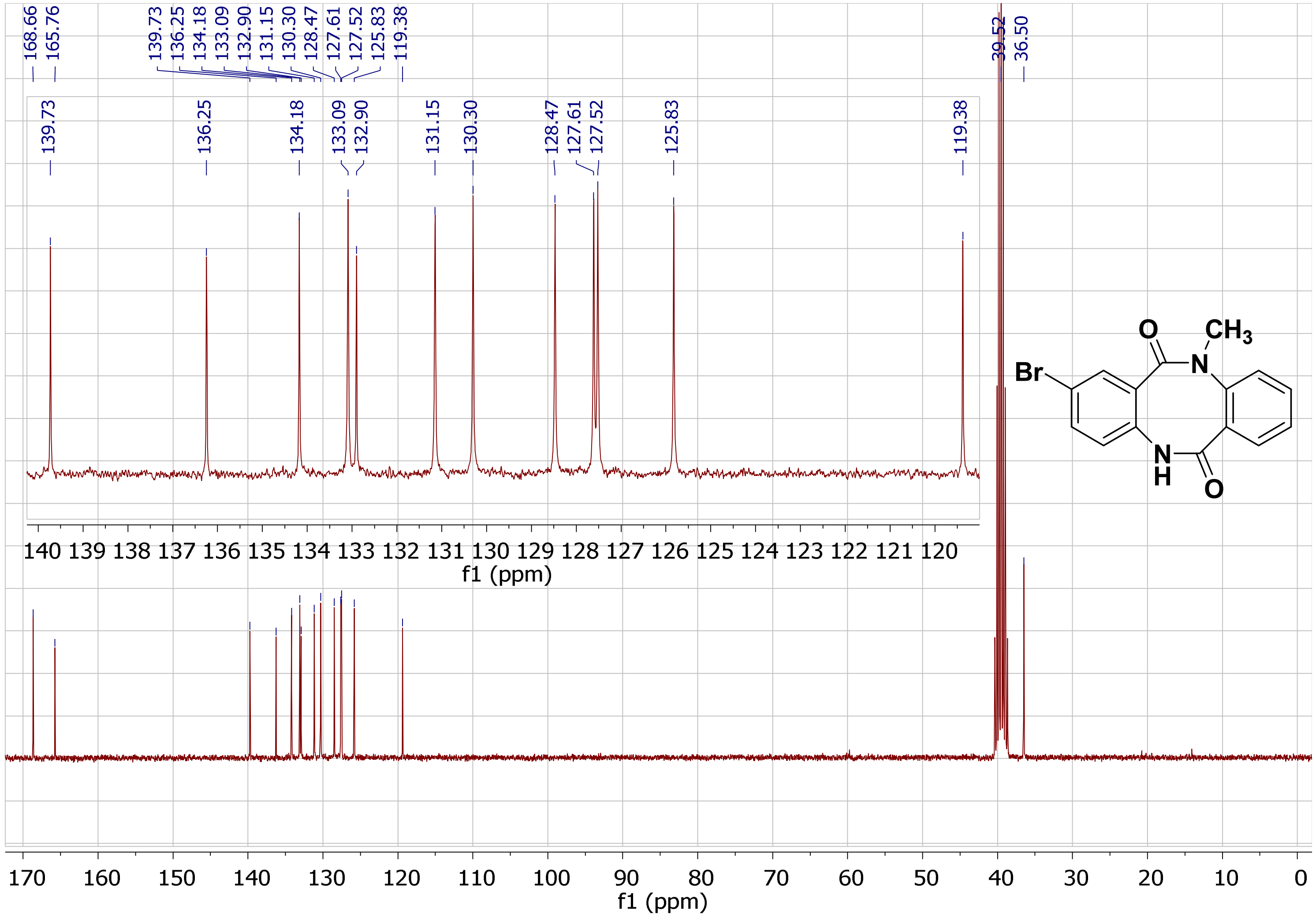

Figure 37S. 13C NMR spectrum of 2-bromo-11-methyldibenzo[*b*,*f*][1,5]diazocine-6,12(5*H*,11*H*)-dione (**10e**)

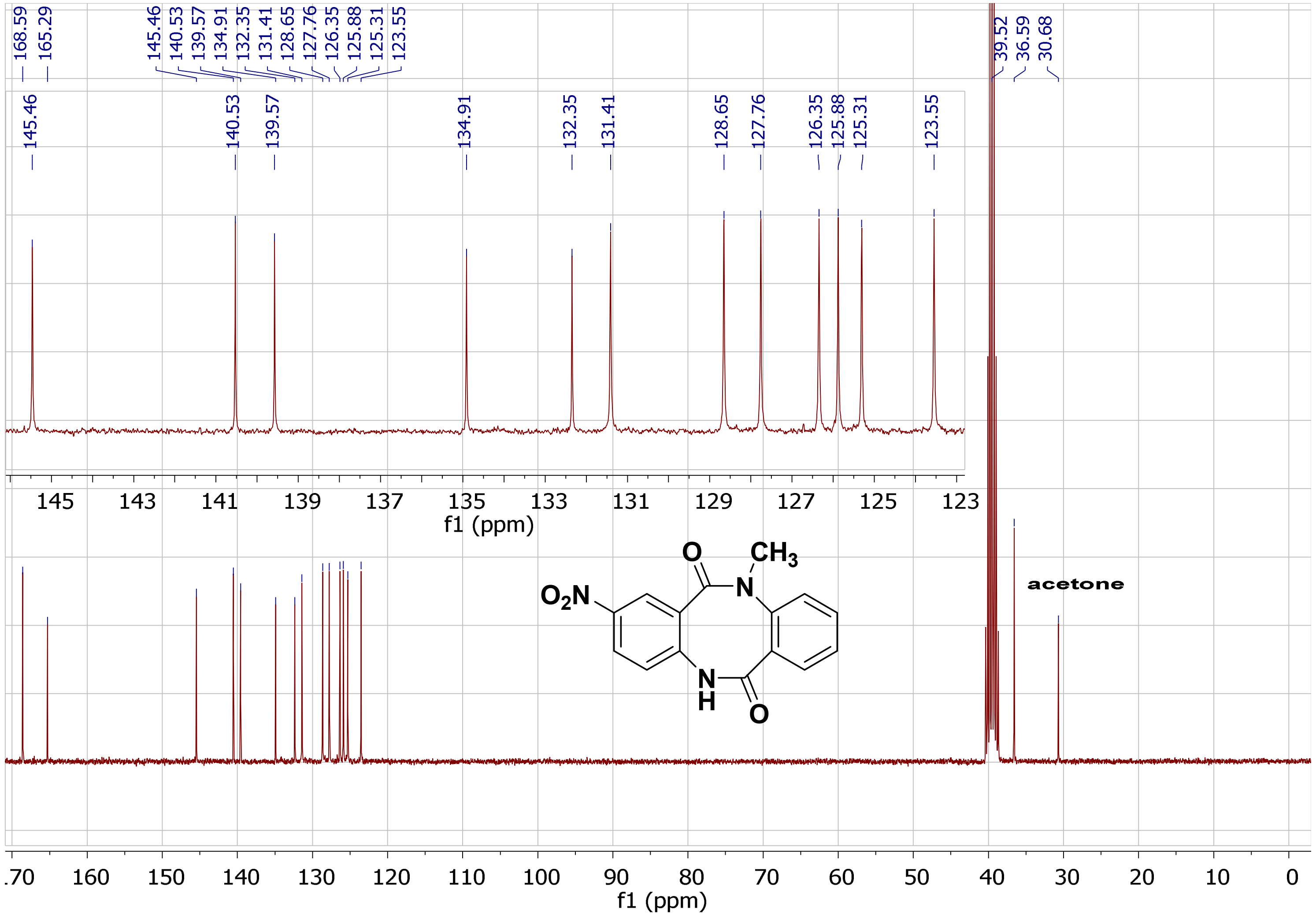

Figure 38S. 13C NMR spectrum of 11-methyl-2-nitrodibenzo[*b*,*f*][1,5]diazocine-6,12(5*H*,11*H*)-dione (**10f**)

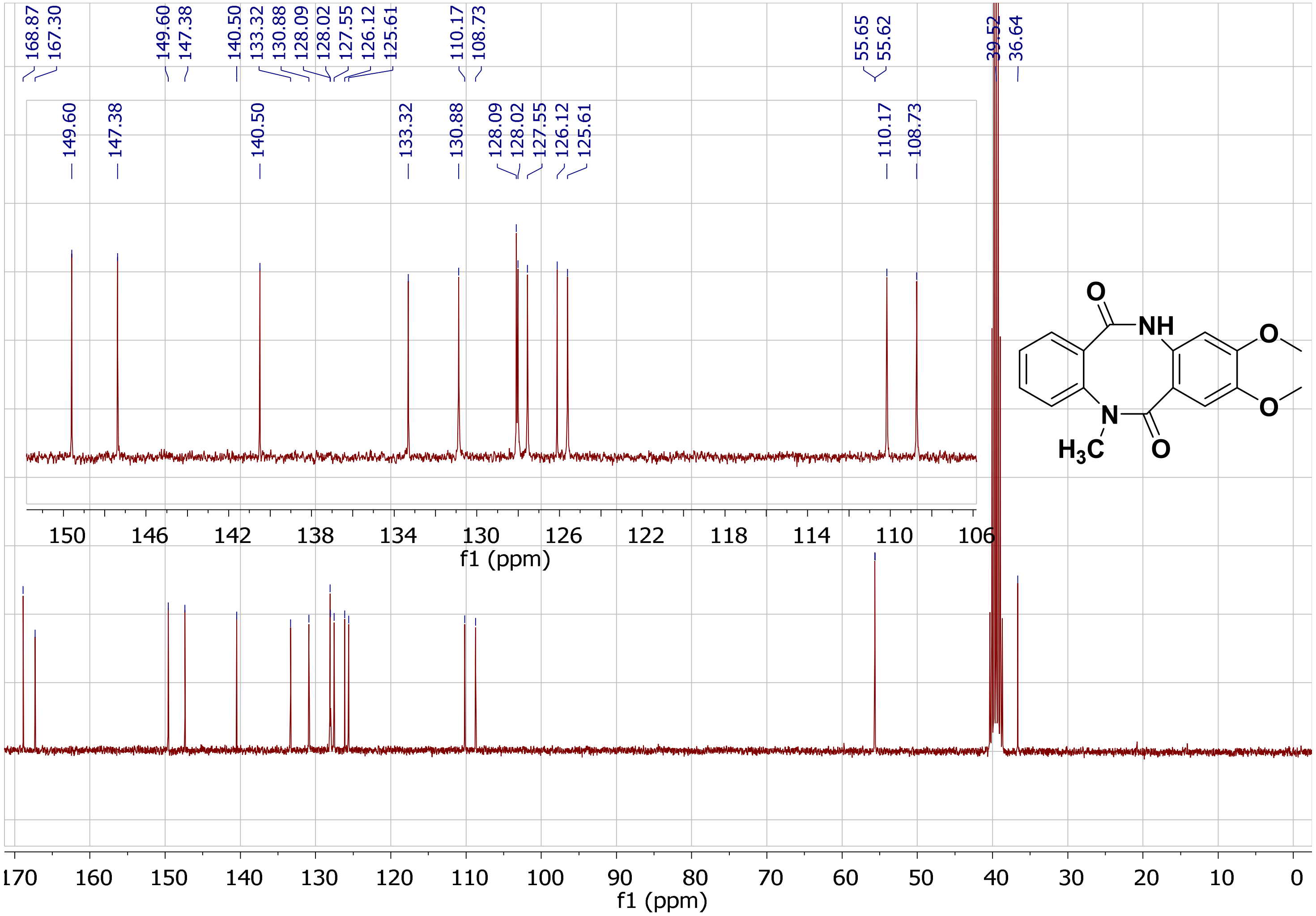

Figure 39S. 13C NMR spectrum of 2,3-dimethoxy-11-methyldibenzo[*b,f*][1,5]diazocine-6,12(5*H*,11*H*)-dione (**10g**)

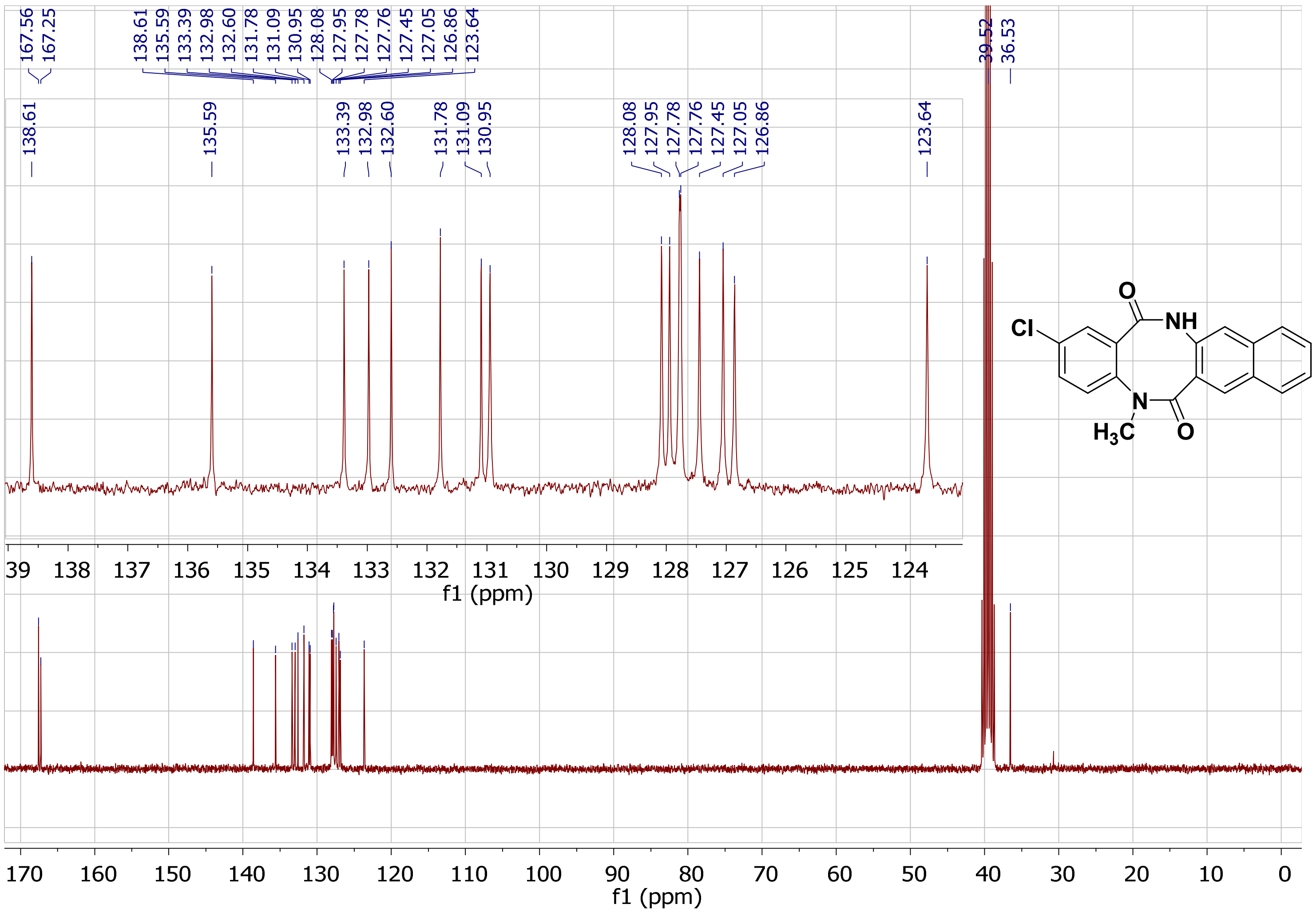

Figure 40S. 13C NMR spectrum of 2-chloro-5-methylbenzo[*b*]naphtho[*2,3-f*][1,5]diazocine-6,14(5*H*,13*H*)-dione (**10h**)

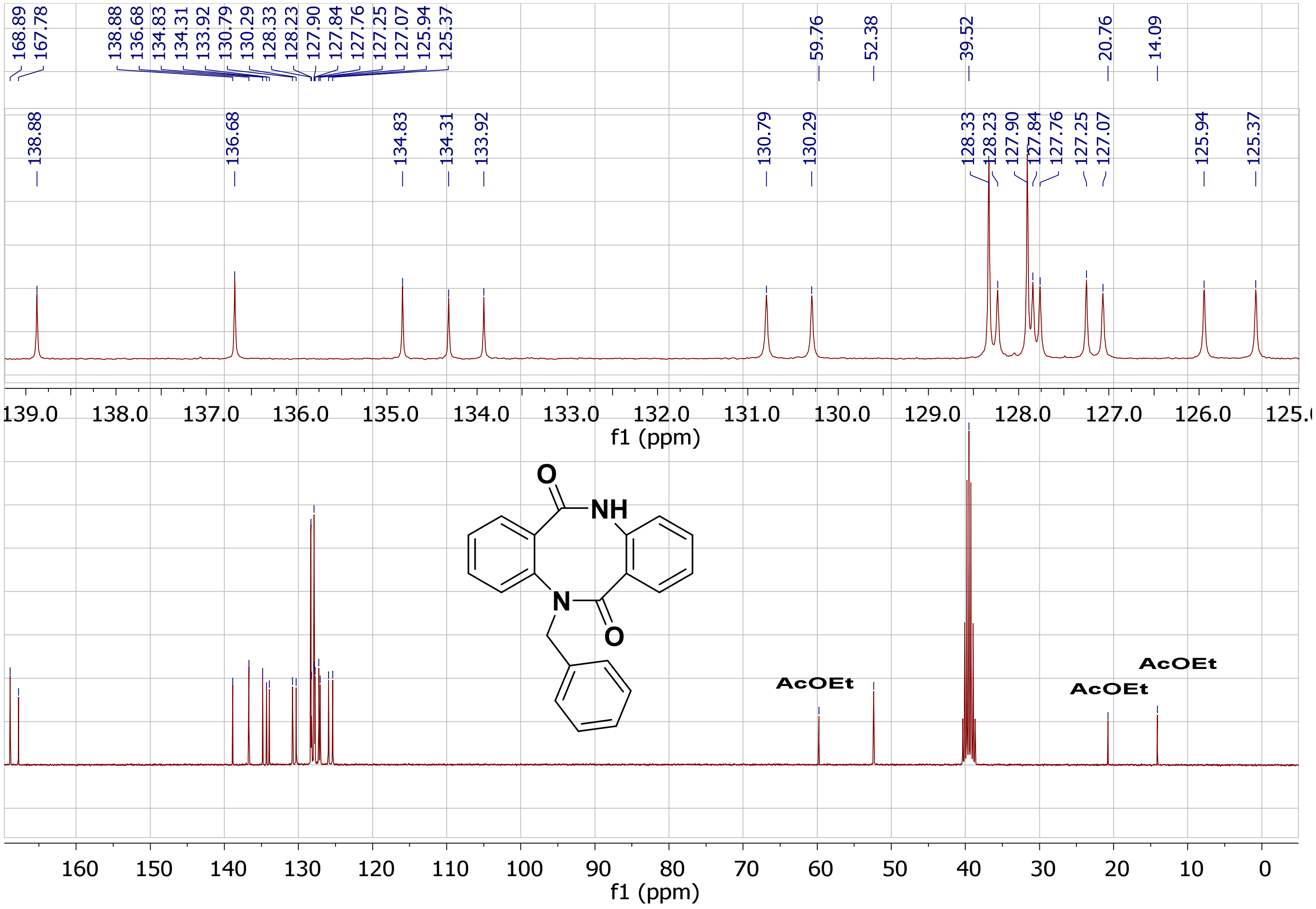

Figure 41S. 13C NMR spectrum of 5-benzyldibenzo[*b*,*f*][1,5]diazocine-6,12(5*H*,11*H*)-dione (**10i**)

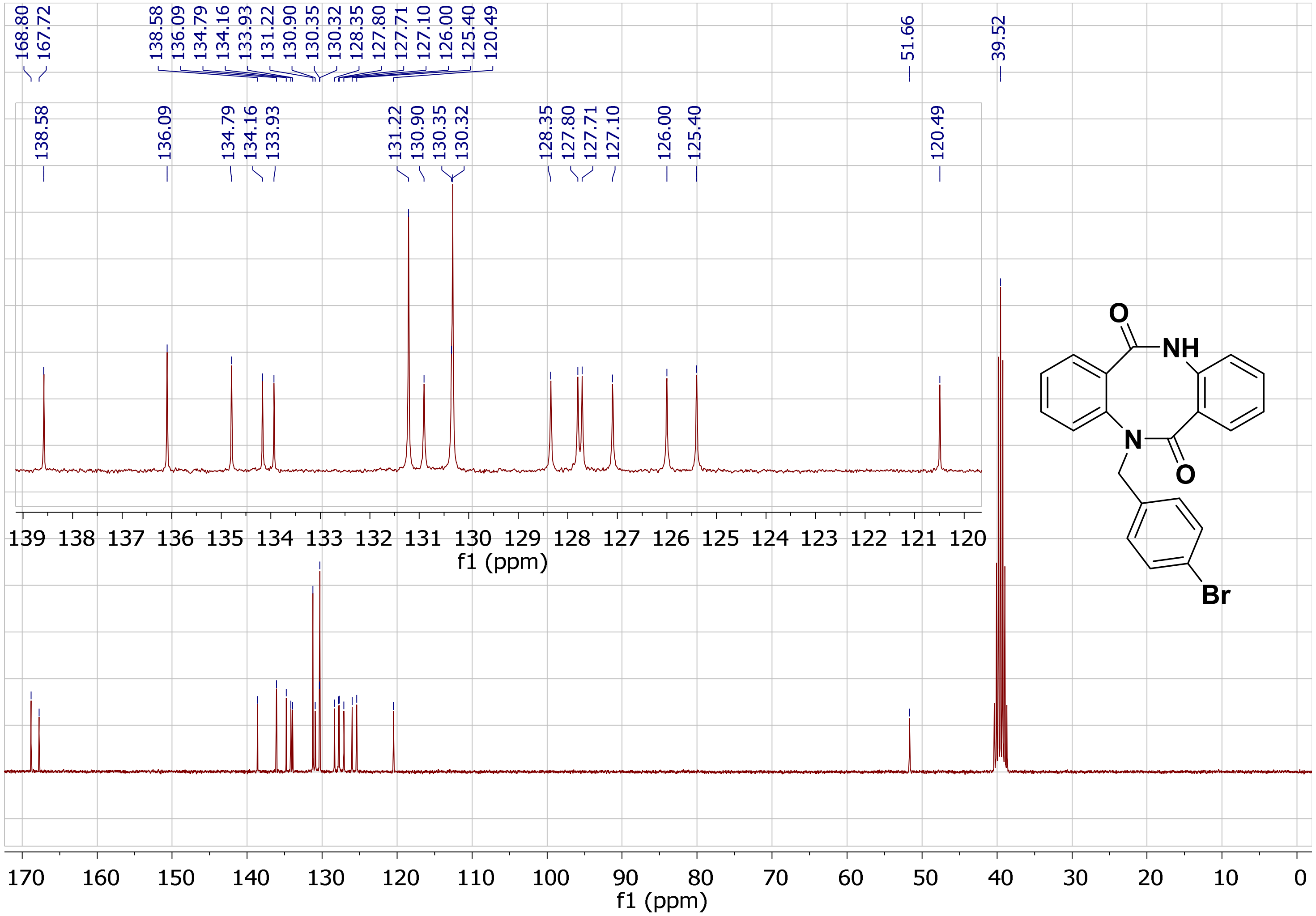

Figure 42S. 13C NMR spectrum of 5-(4-bromobenzyl)dibenzo[*b*,*f*][1,5]diazocine-6,12(5*H*,11*H*)-dione (**10j**)

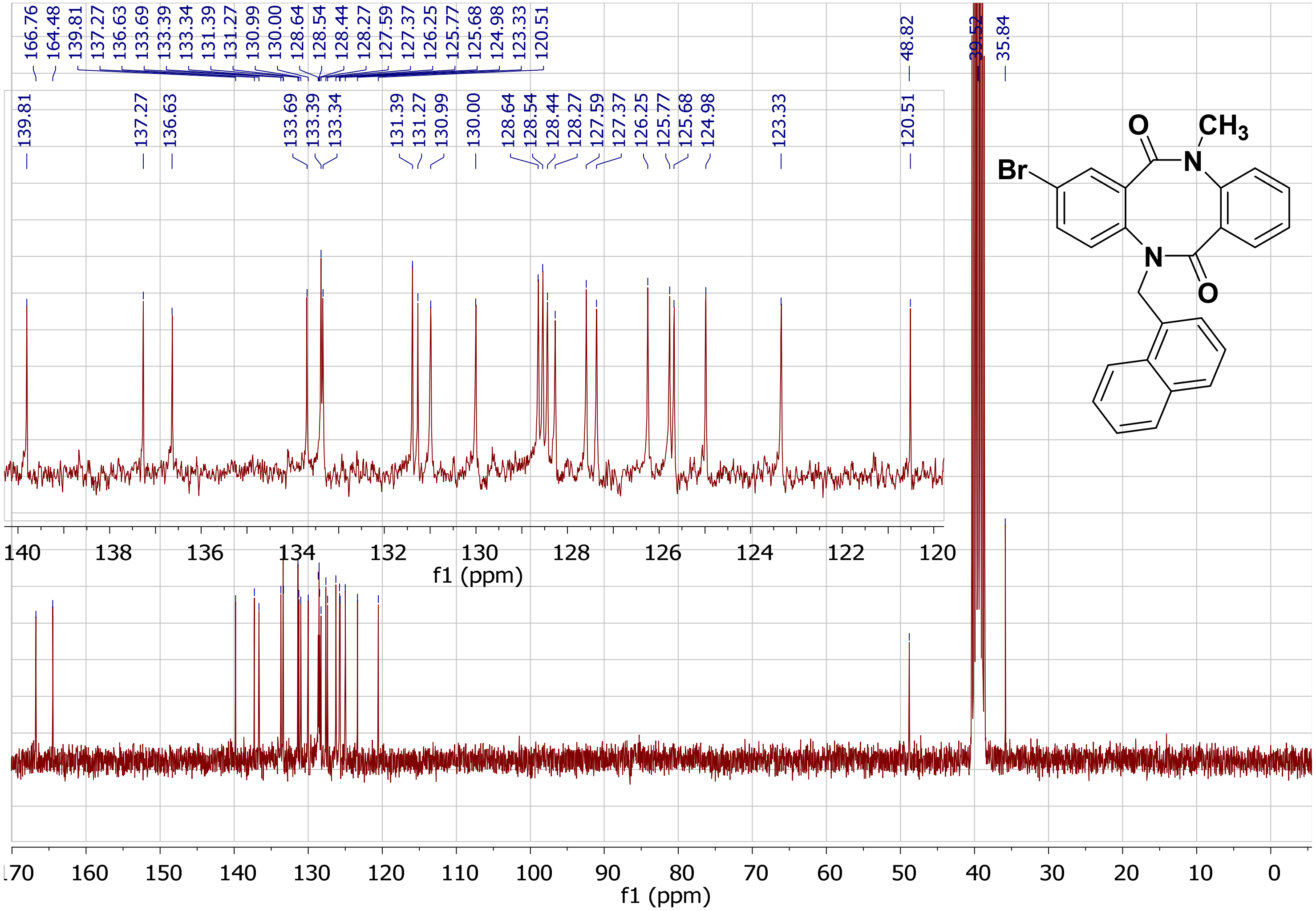

Figure 43S. 13C NMR spectrum of 2-bromo-11-methyl-5-(naphthalen-1-ylmethyl)dibenzo[*b*,*f*][1,5]diazocine-6,12(5*H*,11*H*)-dione (**10k**)

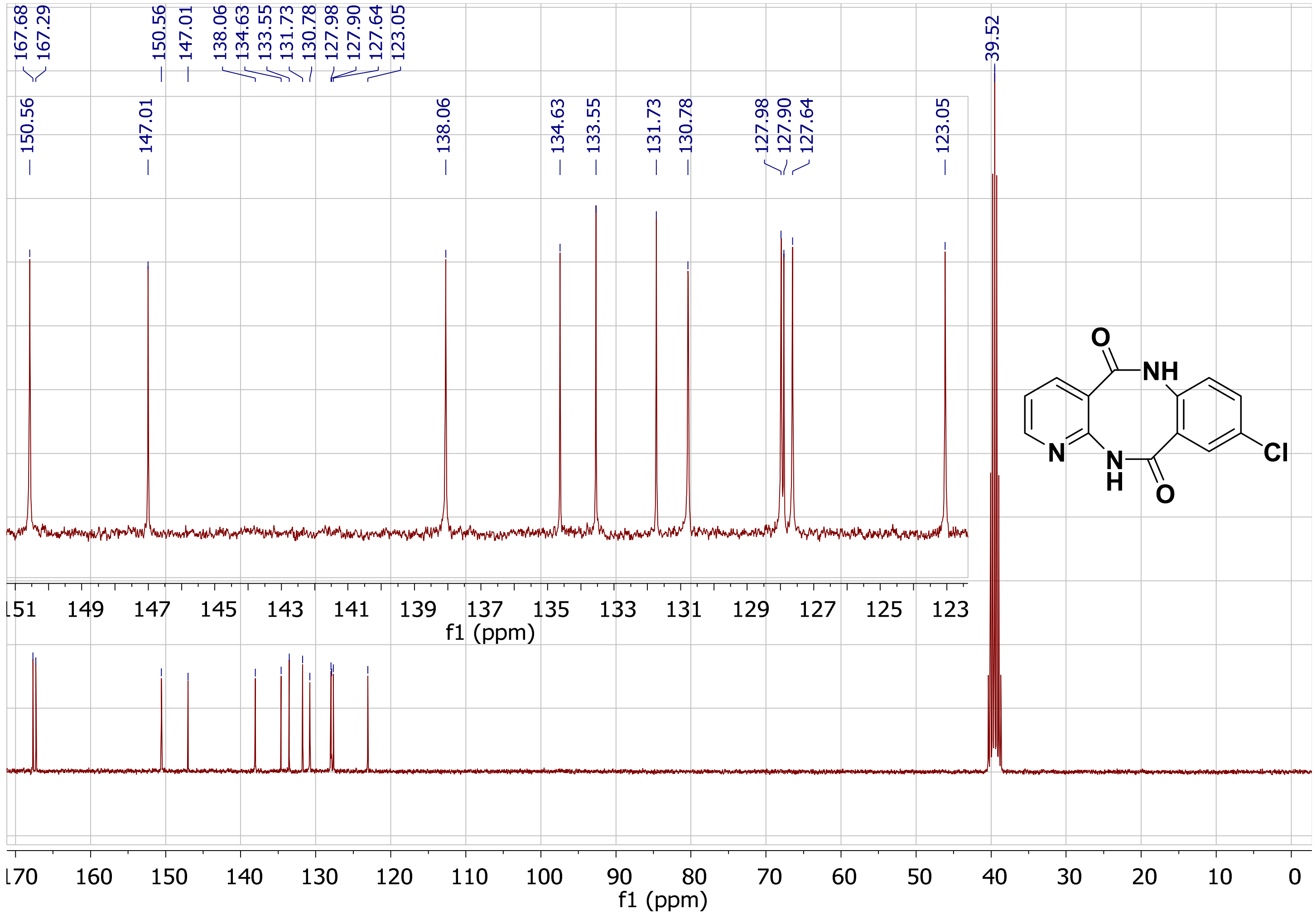

Figure 44S. 13C NMR spectrum of 8-chloropyrido[3,2-*c*][1,5]benzodiazocine-5,11(6*H*,12*H*)-dione (**10l**)

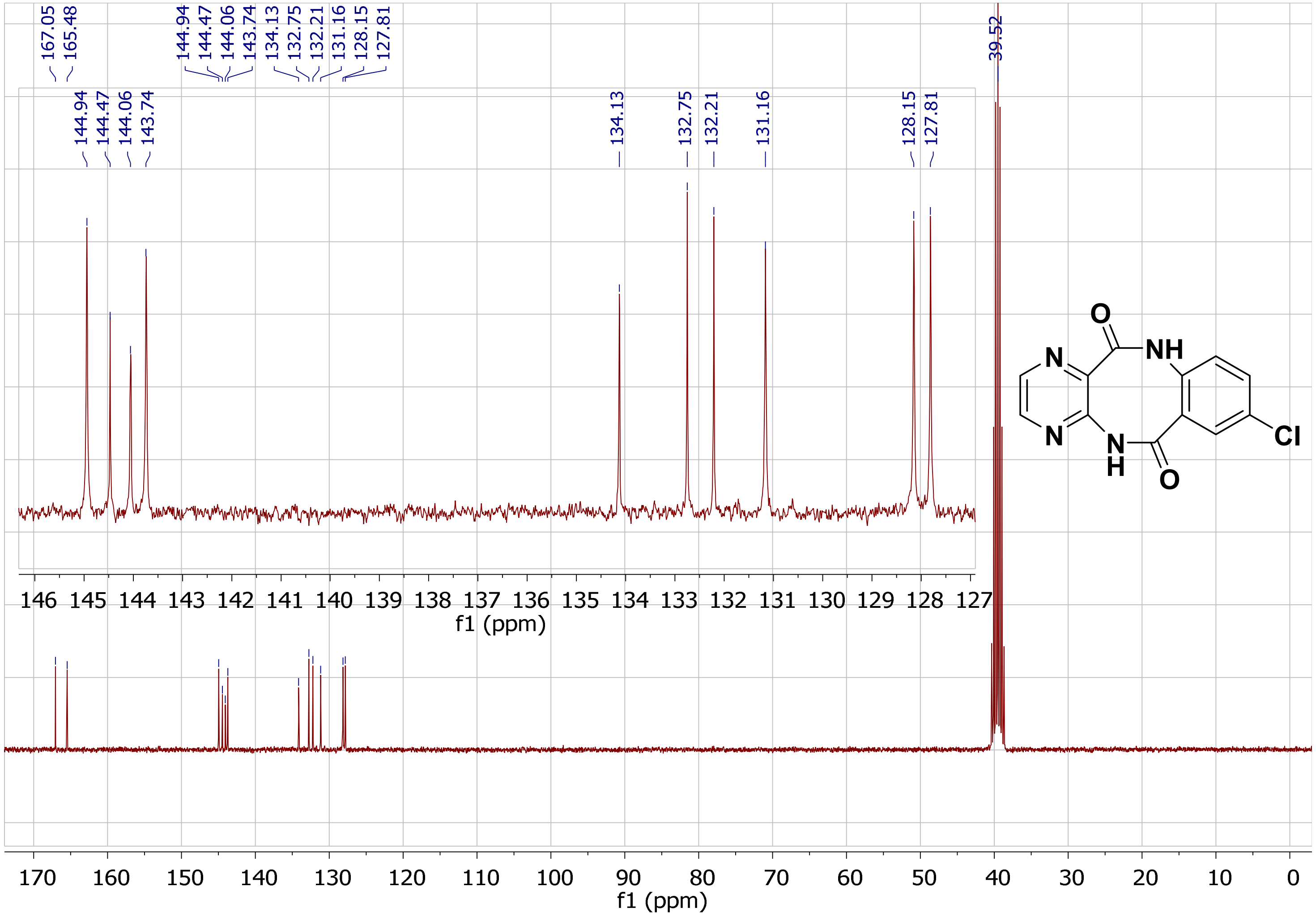

Figure 45S. 13C NMR spectrum of 8-chloropyrazino[3,2-*c*][1,5]benzodiazocine-6,12(5*H*,11*H*)-dione (**10m**)

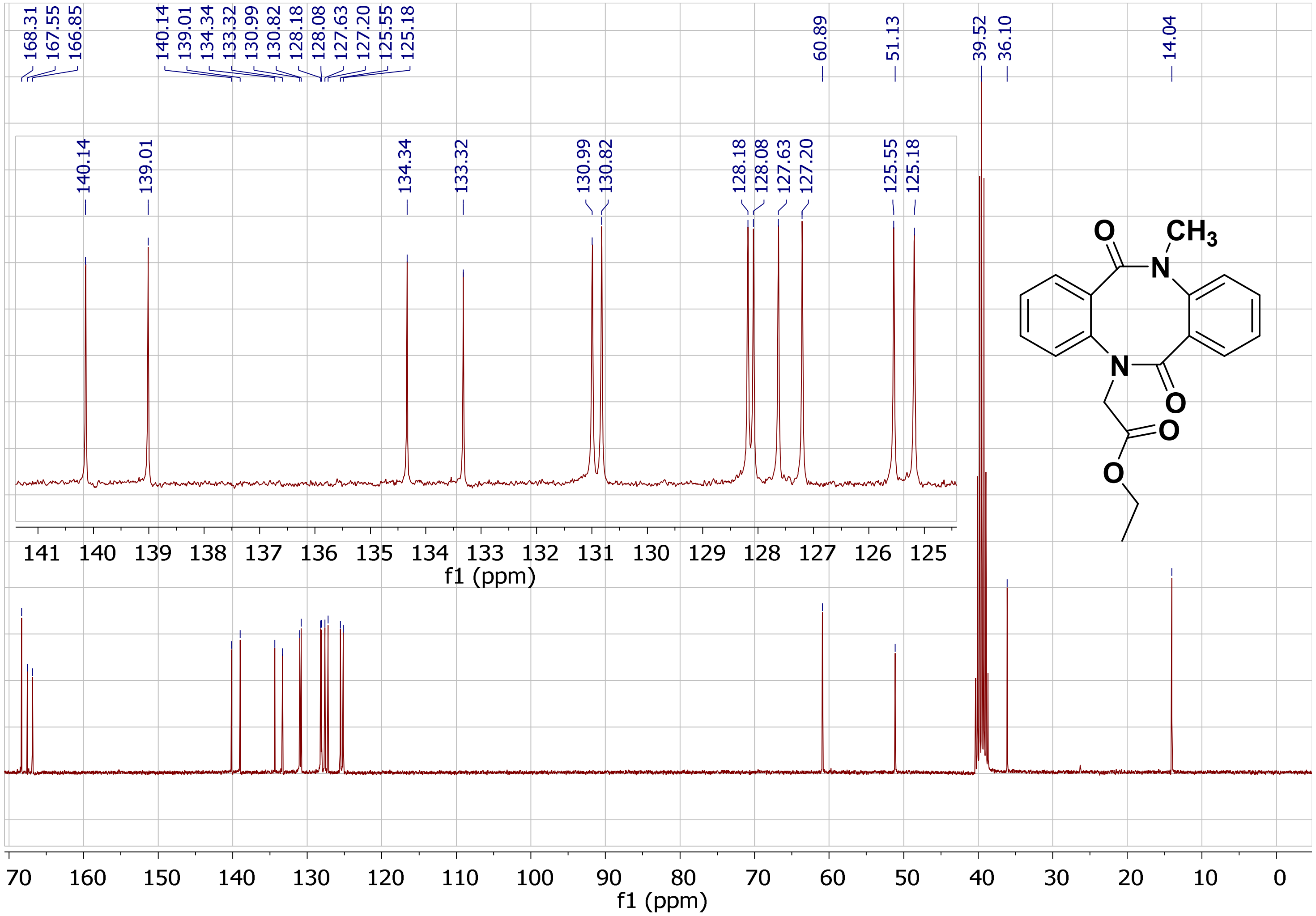

Figure 46S. 13C NMR spectrum of ethyl 2-(11-methyl-6,12-dioxo-11,12-dihydrodibenzo[*b*,*f*][1,5]diazocin-5(6*H*)-yl)acetate (**10n**)

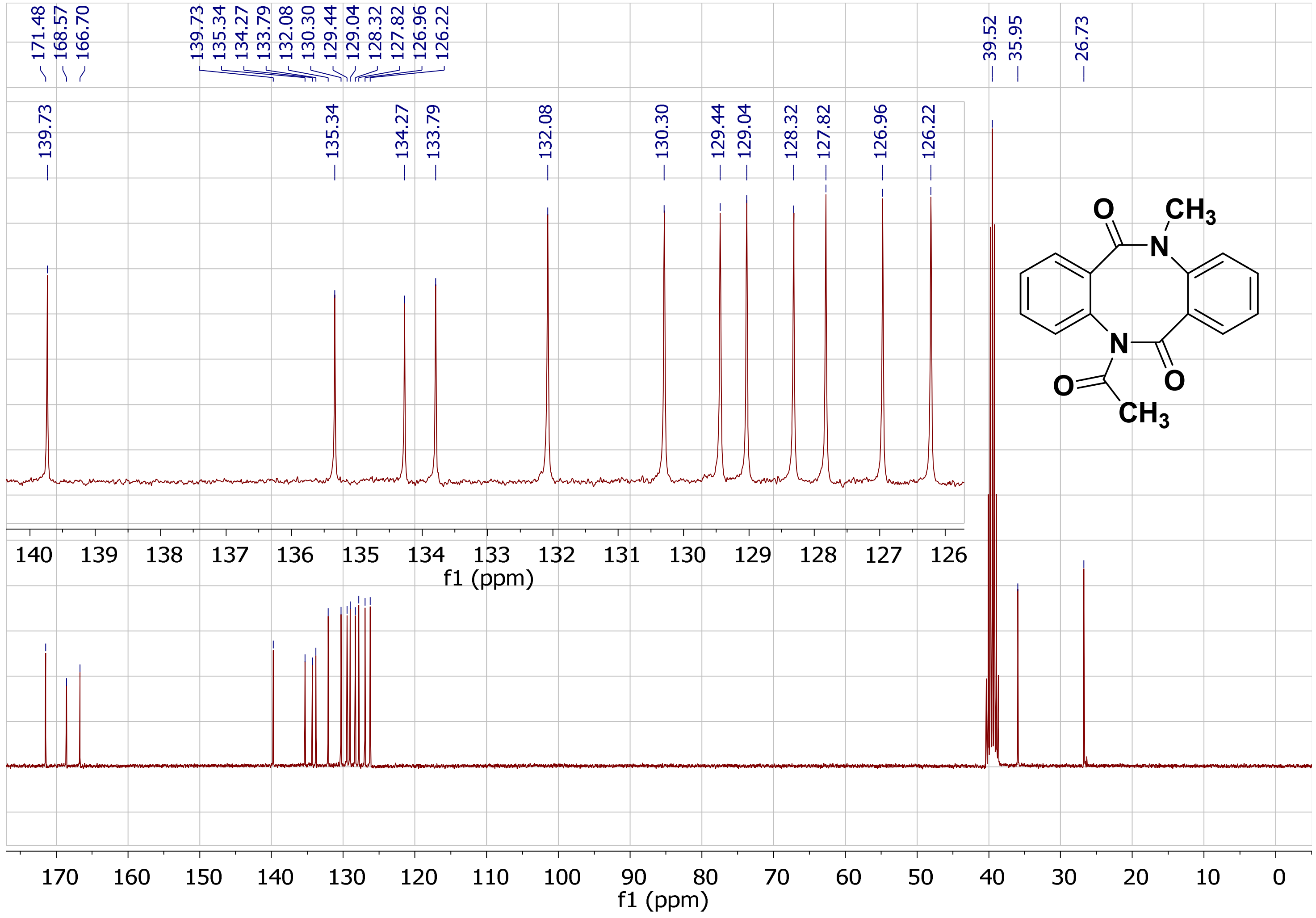

Figure 47S. 13C NMR spectrum of 5-acetyl-11-methyldibenzo[*b*,*f*][1,5]diazocine-6,12(5*H*,11*H*)-dione (**10o**)

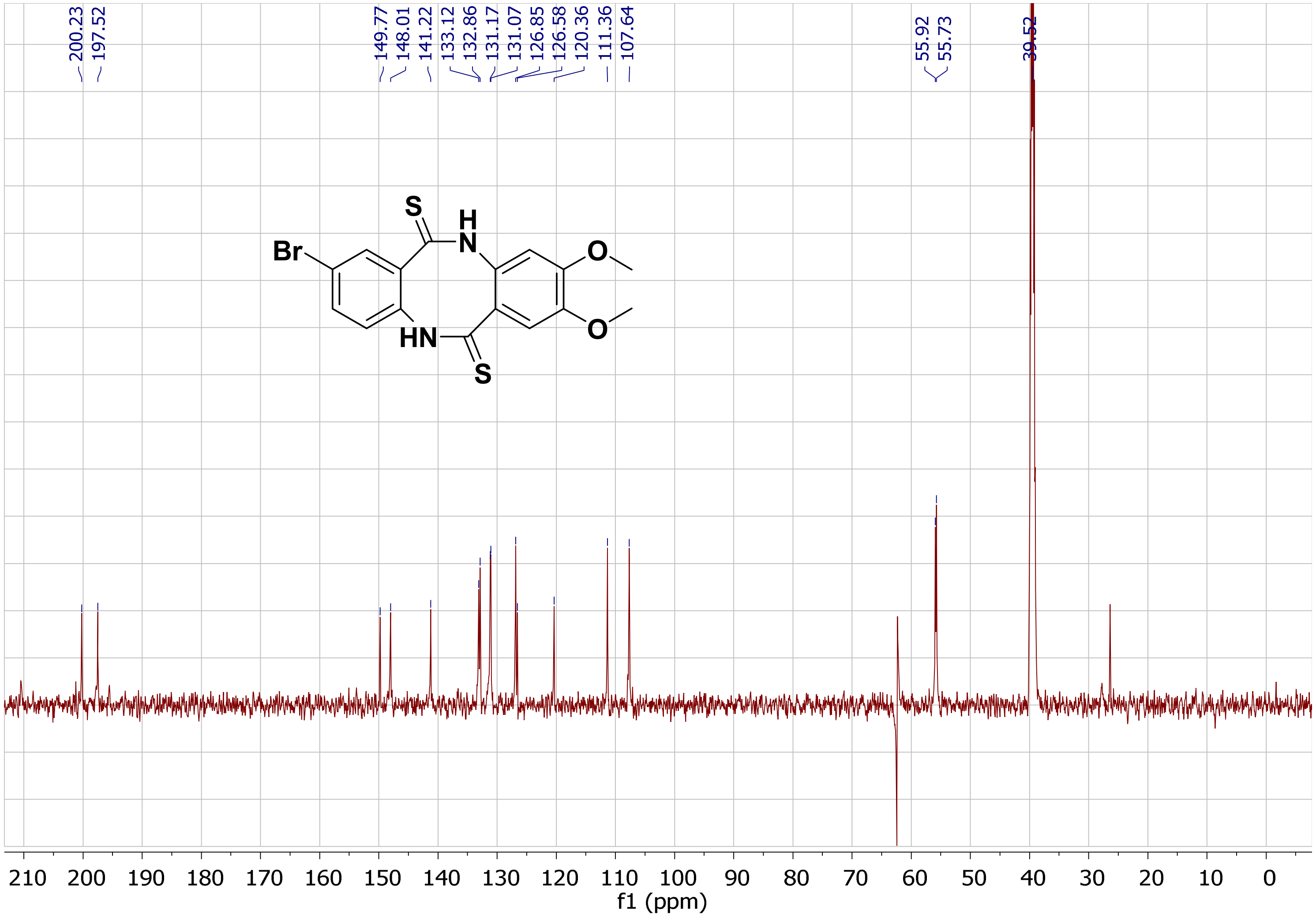

Figure 48S. 13C NMR spectrum of 8-bromo-2,3-dimethoxydibenzo[*b*,*f*][1,5]diazocine-6,12(5*H*,11*H*)-dithione (**10p**)

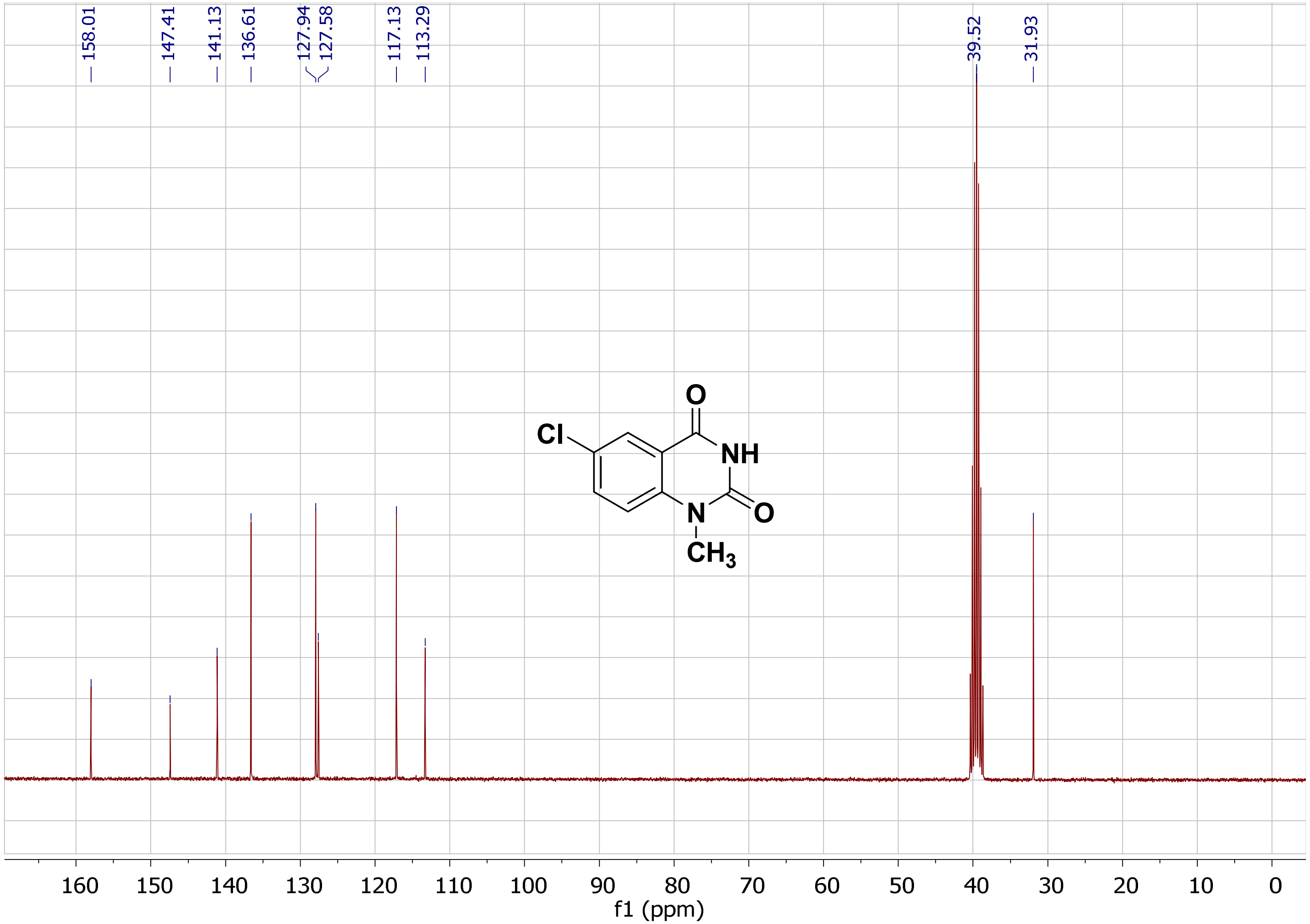

Figure 49S. 13C NMR spectrum of 6-chloro-1-methyl-1*H*-benzo[*d*][1,3]oxazine-2,4-dione (**13f**)

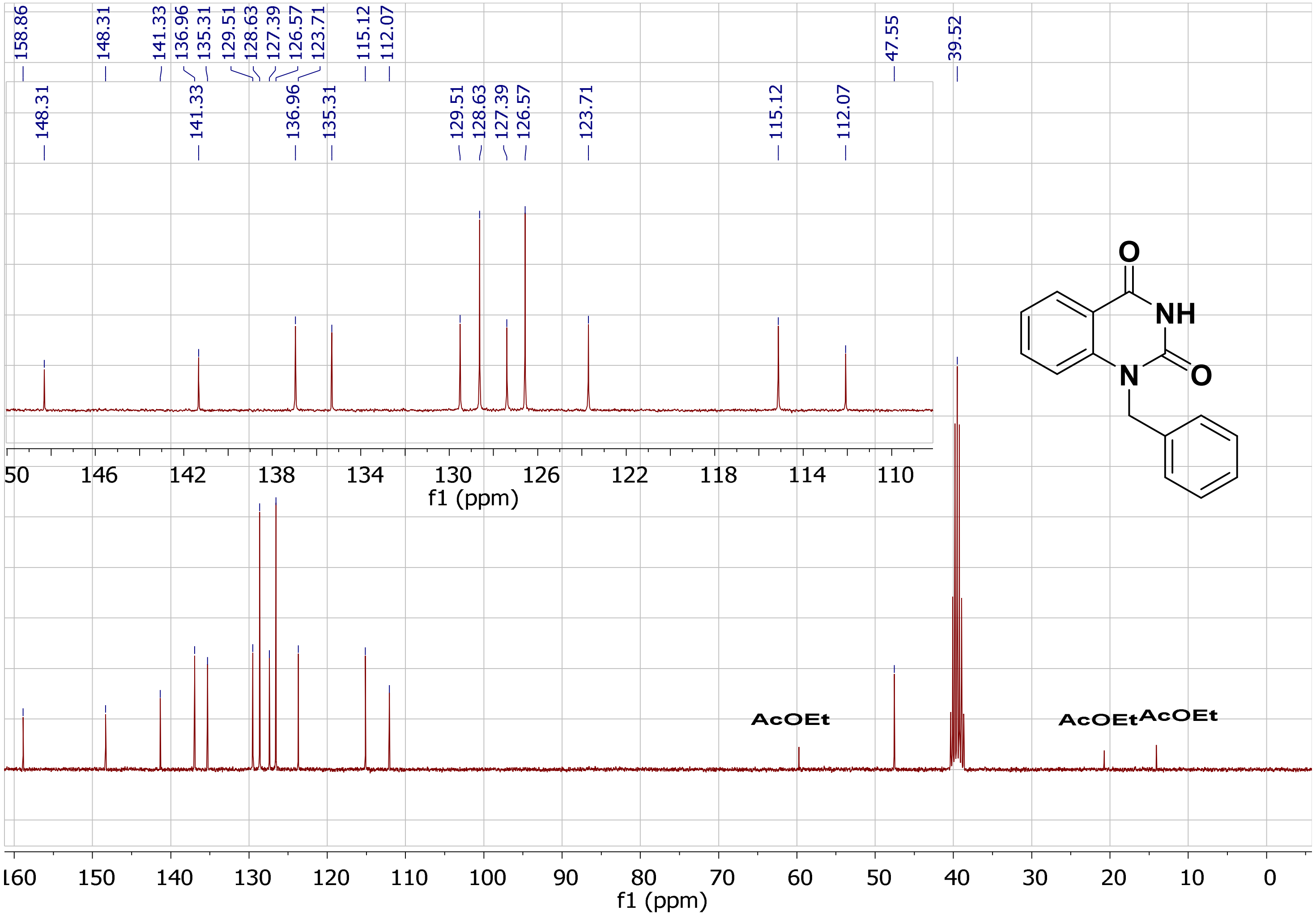

Figure 50S. 13C NMR spectrum of 1-benzyl-1*H*-benzo[*d*][1,3]oxazine-2,4-dione (**13g**)

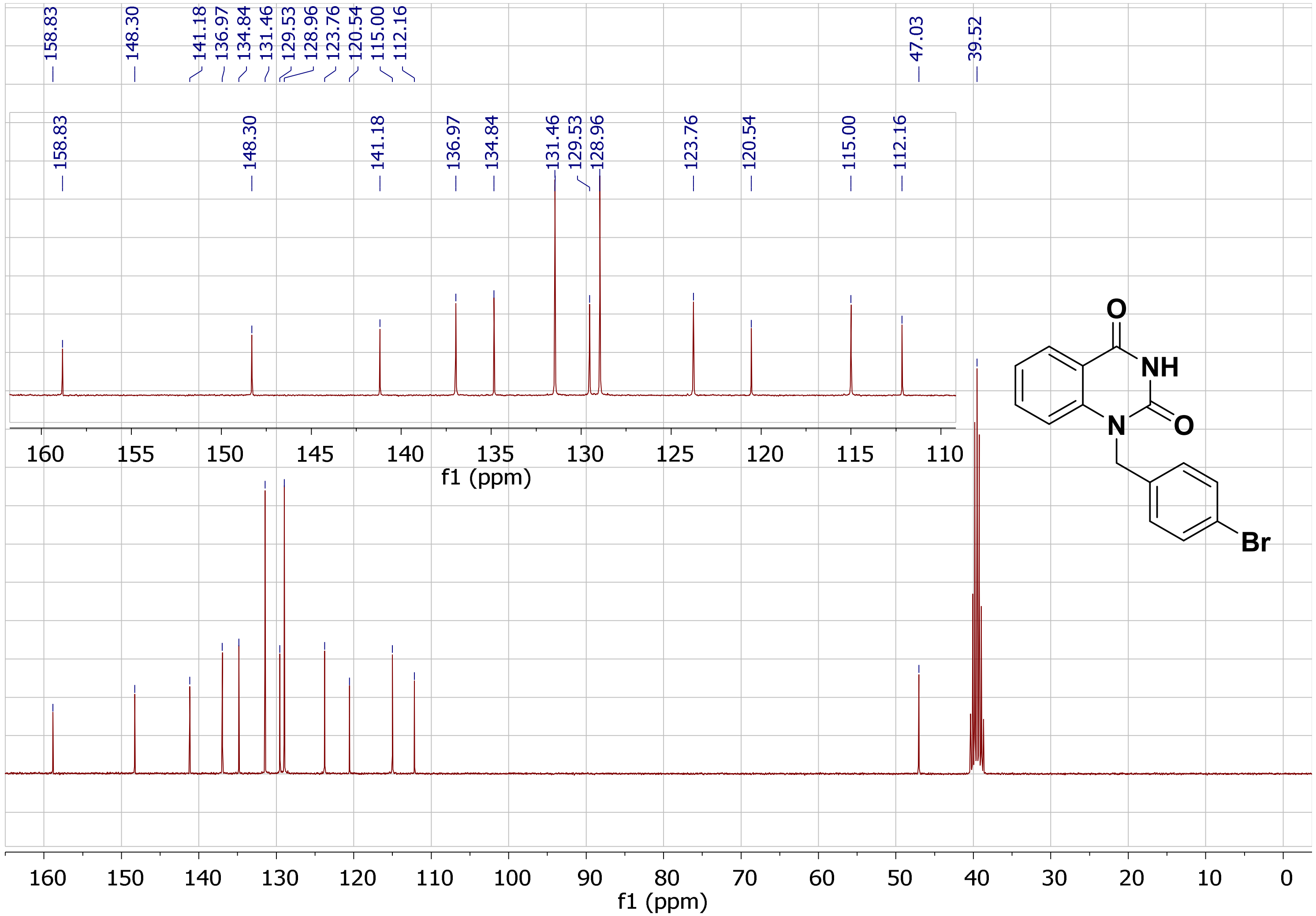

Figure 51S. 13C NMR spectrum of 1-(4-bromobenzyl)-1*H*-benzo[*d*][1,3]oxazine-2,4-dione (**13h**)

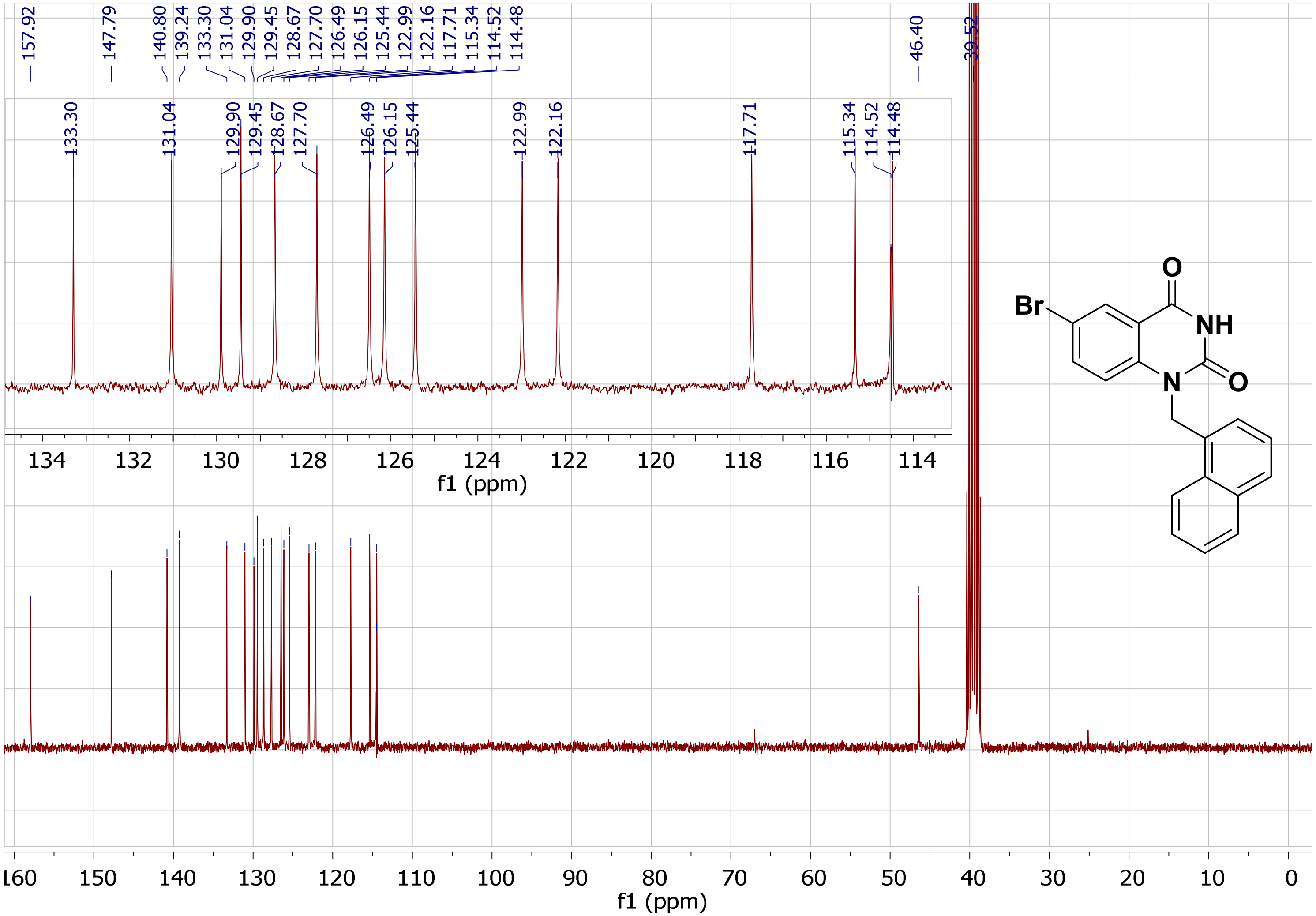

Figure 52S. 13C NMR spectrum of 6-bromo-1-(naphthalen-1-ylmethyl)-1*H*-benzo[*d*][1,3]oxazine-2,4-dione (**13i**)

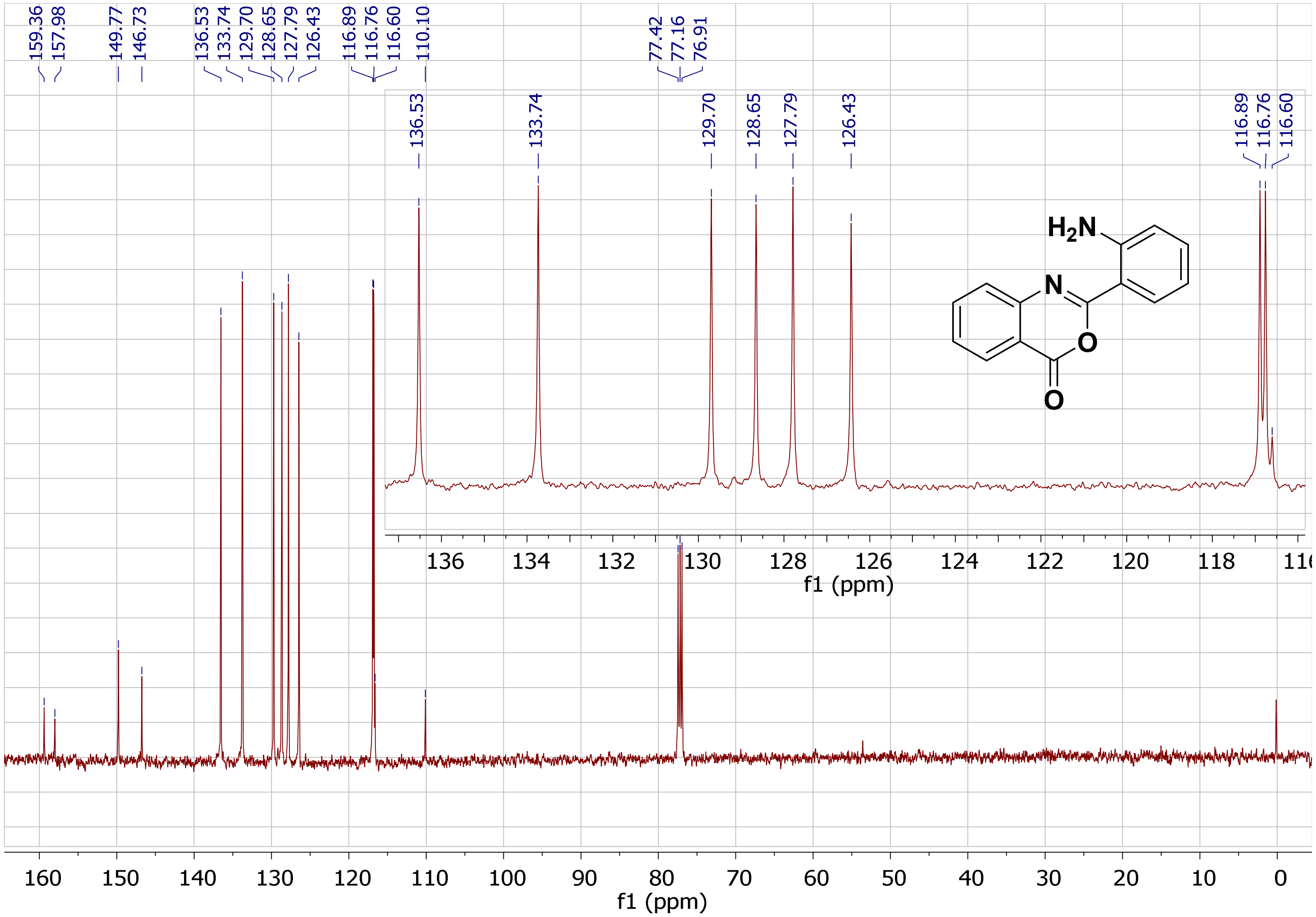

Figure 53S. 13C NMR spectrum of 2-(2-aminophenyl)-4*H*-benzo[*d*][1,3]oxazin-4-one (**12**)

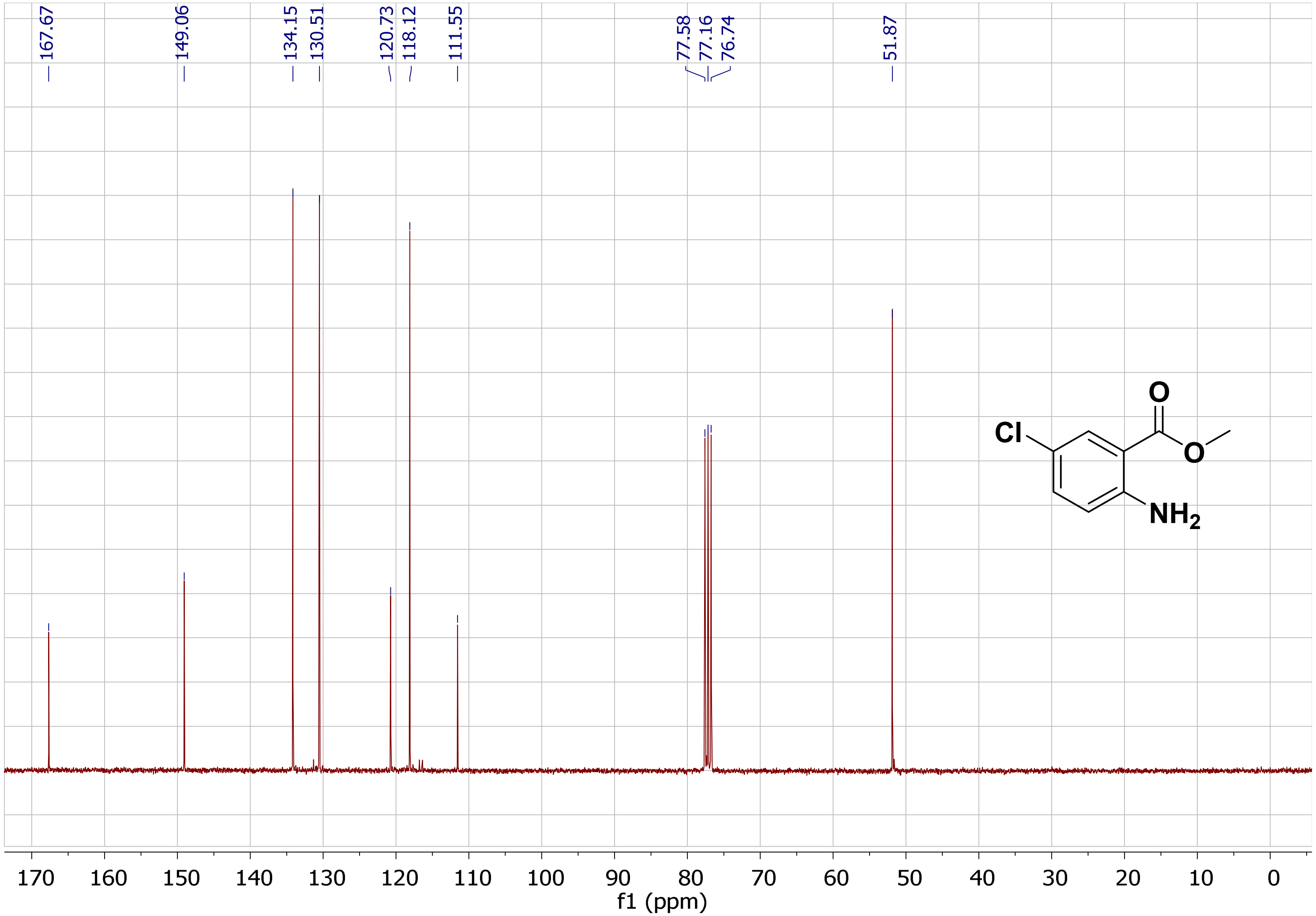

Figure 54S. 13C NMR spectrum of methyl 2-amino-5-chlorobenzoate (**20**)

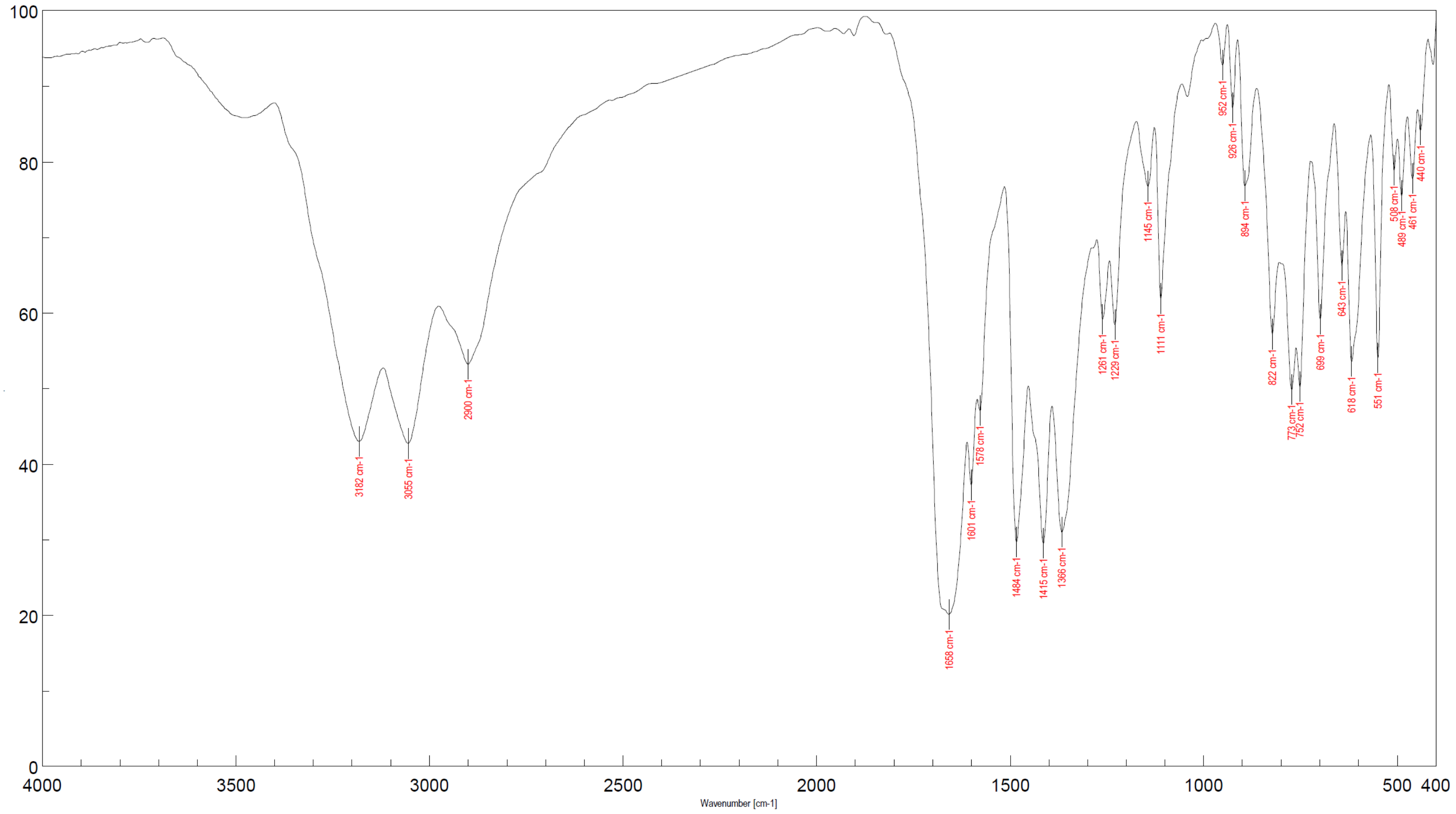

Figure 55S. IR spectrum of 2-chlorodibenzo[*b*,*f*][1,5]diazocine-6,12(5*H*,11*H*)-dione (**10a**)

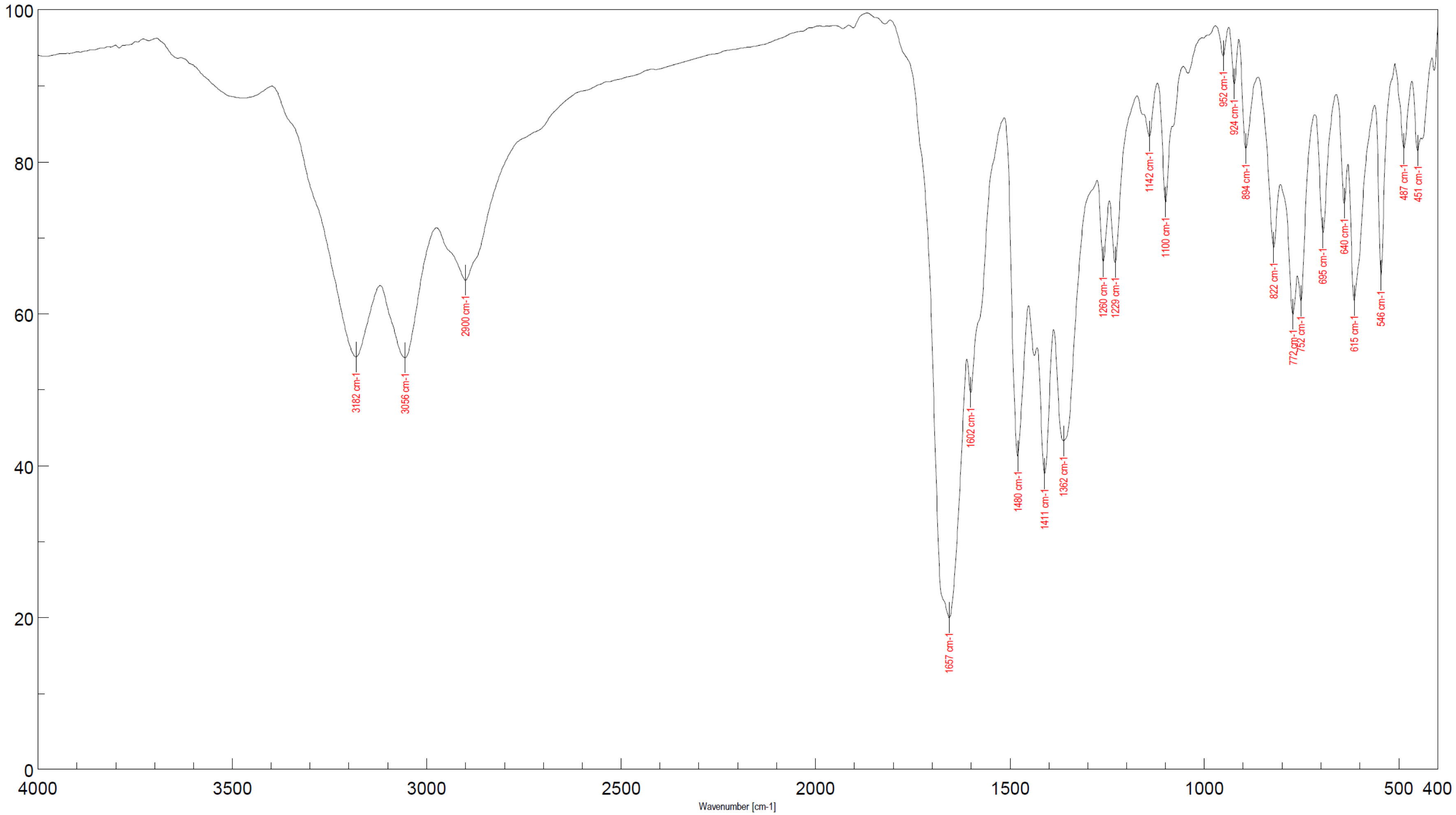

Figure 56S. IR spectrum of 2-bromodibenzo[*b*,*f*][1,5]diazocine-6,12(5*H*,11*H*)-dione (**10b**)

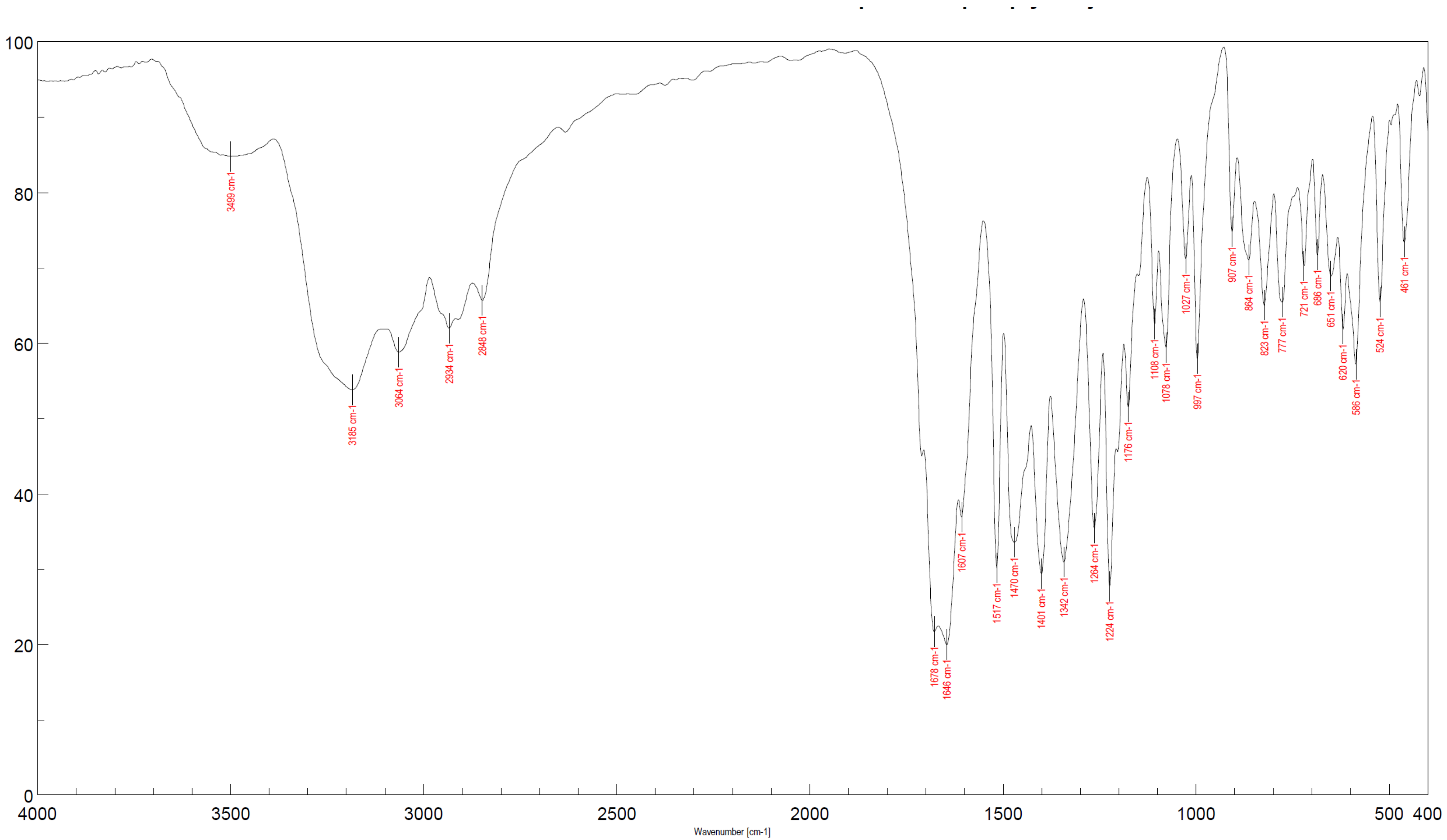

Figure 57S. IR spectrum of 8-bromo-2,3-dimethoxydibenzo[*b*,*f*][1,5]diazocine-6,12(5*H*,11*H*)-dione (**10c**)
